# Herceptin® (trastuzumab) in HER2-positive early breast cancer: a systematic review and cumulative network meta-analysis

**DOI:** 10.1186/s13643-018-0854-y

**Published:** 2018-11-14

**Authors:** Florence R. Wilson, Megan E. Coombes, Christine Brezden-Masley, Mariya Yurchenko, Quinlan Wylie, Reuben Douma, Abhishek Varu, Brian Hutton, Becky Skidmore, Chris Cameron

**Affiliations:** 1 Cornerstone Research Group Inc., Suite 204, 3228 South Service Road, Burlington, ON L7N 3H8 Canada; 2 Hoffmann-La Roche Limited, Mississauga, ON Canada; 3 St. Michael’s Hospital, Toronto, ON Canada; 4 Ottawa Hospital Research Institute, Ottawa, ON Canada; 5 Public Health and Preventative Medicine, University of Ottawa School of Epidemiology, Ottawa, ON Canada; 6 Independent Information Specialist, Ottawa, ON Canada

**Keywords:** Early breast cancer, HER2-positive breast cancer, Network meta-analysis, Survival, Systematic review, Trastuzumab

## Abstract

**Background:**

Originator trastuzumab (Herceptin®; H) is an antibody-targeted therapy to treat patients with human epidermal growth factor receptor 2-positive (HER2+) early breast cancer (EBC). We investigated the overall survival (OS) advantage conferred by the addition of H to chemotherapy for HER2+ EBC patients and how the OS advantage changed over time.

**Methods:**

A systematic literature review (SLR) identified randomized controlled trials (RCTs) and non-randomized studies (NRSs) published from January 1, 1990 to January 19, 2017, comparing systemic therapies used in the neoadjuvant/adjuvant settings to treat HER2+ EBC patients. Bayesian cumulative network meta-analyses (cNMAs) of OS were conducted to assess the published literature over time. Heterogeneity was assessed through sensitivity and subgroup analyses.

**Results:**

The SLR identified 31 unique studies (28 RCTs, 3 NRSs) included in the OS analyses from 2008 to 2016. In the reference case cNMA (RCTs alone), initial evidence demonstrated an OS advantage for H/chemotherapy compared with chemotherapy alone in HER2+ EBC patients. As additional OS data were published, the precision around this survival benefit strengthened over time. Both H/anthracycline-containing chemotherapy and H/non-anthracycline-containing chemotherapy regimens provided similar OS advantages for HER2+ EBC patients.

**Conclusion:**

This analysis represents the most comprehensive SLR/cNMA to date of published OS data in HER2+ EBC studies. These findings demonstrate why H/chemotherapy is now the established standard of care in HER2+ EBC. In the case of H, the benefits of early patient access far outweighed the risk of waiting for more precise information.

**Systematic review registration:**

PROSPERO CRD42017055763

**Electronic supplementary material:**

The online version of this article (10.1186/s13643-018-0854-y) contains supplementary material, which is available to authorized users.

## Background

Human epidermal growth factor receptor 2-positive (HER2+) breast cancer (BC) is an aggressive disease that makes up approximately 20% of all invasive BC [1, 2]. Early stage BC (EBC) describes disease that is detected in the breast and nearby lymph nodes but has not spread to distant areas of the body [3]. Recommended treatment of EBC is a multi-step approach that often includes neoadjuvant therapy, surgery, and adjuvant therapy [4–6]. Therapy for HER2+ EBC should be based on a patient’s predicted sensitivity to treatments, underlying comorbidities, likelihood of benefit, and risk of relapse [4, 6]. The development of HER2-targeted therapies has revolutionized the treatment of HER2+ BC that was previously associated with high relapse and mortality rates. Current guidelines suggest that HER2+ EBC patients should receive chemotherapy and anti-HER2 agents, such as originator trastuzumab (Herceptin®; H) [4, 6, 7].

As an antibody-targeted therapy, H binds to the extracellular domain IV of HER2, thereby inhibiting downstream cell signaling implicated in cell proliferation, motility, adhesion, and survival [8]. Initially approved by all major regulatory bodies for the treatment of HER2+ metastatic breast cancer (MBC) [9–12], approved use of H was expanded to HER2+ EBC in 2006 [13–15]. Clinical trials in HER2+ EBC and MBC have established that treatment with H/chemotherapy increases disease-free and overall survival (OS) compared with chemotherapy alone [16–20]. Concomitant administration of anthracyclines with H can produce cardiotoxic effects, so non-anthracycline-containing regimens may be beneficial for some patients [5, 21, 22]. A sequential regimen of anthracyclines and H/taxanes is appropriate for most patients [4, 5]. H for 1 year administered with an acceptable chemotherapy regimen is the recommended standard of care for EBC [4–6].

The efficacy of H for the treatment of HER2+ EBC has been demonstrated in phase II and phase III clinical trials; however, an assessment of the accumulation of publicly available evidence over time has not been made. Here, we report the results of a systematic literature review (SLR) and cumulative network meta-analysis (cNMA) that evaluates the survival advantage conferred by the addition of H to chemotherapy regimens for the treatment of HER2+ EBC, and how the certainty of this survival advantage has changed over time.

## Methods

### Search strategy and selection criteria

The SLR/NMA protocol is registered on PROSPERO (CRD42017055763), https://www.crd.york.ac.uk/PROSPERO, and has been described previously [23]. Our SLR was conducted in accordance with PRISMA guidelines [24]. A completed checklist is provided in the Additional file 1.

Briefly, an SLR was conducted to identify randomized controlled trials (RCTs) and non-randomized studies (NRS) of systemic therapies used in the neoadjuvant/adjuvant settings to treat adults with HER2+ EBC, locally advanced BC, or inflammatory BC. Database searches were performed using a predefined, peer-reviewed search strategy, spanning from January 1, 1990 to January 19, 2017 (Additional file 1) [23]. Studies were reviewed in duplicate based on prespecified eligibility criteria (Additional file 1) [23]. Data were extracted from included studies based on prespecified categories. Hazard ratios (HRs) with 95% confidence intervals (CIs) were extracted for the outcomes of interest. OS was the primary outcome of interest and is the focus of our analyses [23]. No adverse events or safety information were extracted as per protocol, other than the number of deaths. A risk of bias assessment of included studies was completed as described [23].

### Data analysis

Network meta-analysis is an approach that allows the simultaneous comparison of multiple treatments that may not have been compared directly in the same study [25–27]. A traditional NMA provides an assessment of all available evidence at a particular time point. To investigate how the evidence for H has changed over time, we performed a Bayesian cNMA based on well-established methods by the National Institute for Health and Care Excellence (NICE) [28, 29]. A cNMA is a series of NMAs sequenced chronologically based on the publication dates of studies, wherein each NMA incorporates additional studies over time. We evaluated the available evidence for the survival advantage conferred by H/chemotherapy regimens of interest over 2-year intervals from 2008 to 2016. We performed separate NMAs for each time interval so that each NMA included all publicly available evidence published from 1990 until that time point. The publication of newer trial results replaced the corresponding older results for the same trial. Evidence networks show each treatment as a node and comparisons between treatments are shown as lines linking the nodes. Node size reflects sample size and line width reflects the number of studies included in the connection. The networks expand as new evidence is added over time, and nodes and connections increase accordingly.

We categorized studies based on the proportion of HER2+ patients, and only 100% HER2+ EBC patients were included in analyses. For studies that included < 100% HER2+ EBC patients, we extracted OS data from HER2+ subgroups when possible. Our reference case included all RCTs (100% HER2+ patients and HER2+ subgroups), and we conducted sensitivity analyses for (1) RCTs with 100% HER2+ patients, and (2) RCTs with 100% HER2+ patients, RCTs with HER2+ subgroups, and NRS. We focused on pairwise comparisons between the two most widely used H regimens and a reference treatment. The recommended treatment duration for H is 52 weeks [5, 6, 30], and the two most widely used and recommended regimens at this duration are AC-TH_52 weeks_ (anthracycline/taxane-containing chemotherapy with H intravenous [IV]) and TCH_52 weeks_ (non-anthracycline-containing chemotherapy with H IV) [4–7, 13, 15]. A standard chemotherapy regimen consisting of anthracycline/taxane-containing chemotherapy (AC-T) was selected as the reference treatment.

Random effects (RE) models were performed as primary analyses with vague priors assigned to basic parameters throughout. For vague priors, we assumed a uniform distribution (i.e., uniform [0, 5]) for between-study variance, as recommended by NICE [28]. Fixed-effect (FE) models were reported as sensitivity analyses. In accordance with NICE Technical Support Document methods, the log HR was treated as a continuous outcome and the final results were subsequently exponentiated [28, 29]. As a measure of the association between each treatment and its efficacy, Markov chain Monte Carlo methods were used to model HR point estimates and 95% credible intervals (CrIs) for each pairwise comparison. Estimates with 95% CrIs that excluded the null value of 1 were considered to reflect statistically significant differences between interventions. HRs < 1 corresponded to beneficial treatment effects of the first treatment compared with the second treatment. We generated values to show the probability of the first treatment being better than the second treatment within each pairwise comparison (p[better]) [31]. To assess model fit, the posterior residual deviance from each NMA was compared to the corresponding number of unconstrained data points.

Analyses were conducted using WinBUGS (version 1.4.3, MRC Biostatistics Unit, Cambridge, UK) and R (version 3.2.2, R Core Team, Vienna, Austria). Three chains were fitted in WinBUGS for each analysis, with a burn-in of at least 40,000 iterations and subsequent iterations of at least 40,000 (WinBUGS code is available upon request). Model convergence was assessed using trace plots, the Brooks-Gelman-Rubin statistic, and inspection of Monte Carlo errors [28].

### Assessment of heterogeneity and inconsistency

We attempted to conduct sensitivity analyses to combine RCTs and NRS. Including high-quality NRS can allow larger, diverse populations to be captured, and can allow the consideration of treatments that may not have been studied in RCTs; however, including low-quality NRS can introduce confounding bias if the baseline characteristics and risk factors in the treatment groups are substantially different [25, 32, 33]. We assessed the statistical methods of eligible NRS and only included the highest quality studies with appropriately adjusted effect estimates. We also assessed study and patient characteristics in all studies to ensure similarity and to investigate the impact of heterogeneity. A Bayesian hierarchical model that includes a study-design level is generally considered the most flexible for combining RCTs and NRS [34–37], but the structure of our evidence networks did not permit this analysis (Additional file 1). Instead, we performed a sensitivity analysis that naïvely combined RCTs and NRS. To assess the robustness of the reference case analysis, we also conducted a sensitivity analysis using whole survival curves rather than hazard ratios.

We considered the following subgroup analyses: neoadjuvant versus adjuvant therapy, node-positive BC (N1–N3), node-negative BC (N0), hormone receptor-positive (HR+) BC, hormone receptor-negative (HR−) BC, large tumors (≥ 2 cm), and small tumors (< 2 cm). We investigated anthracycline-containing versus non-anthracycline-containing chemotherapy by focusing on the pairwise comparison of AC-TH_52 weeks_ versus TCH_52 weeks_.

Inconsistency in the evidence networks was assessed by comparing the posterior residual deviance and deviance information criterion (DIC) statistics in fitted consistency and inconsistency models [38]. The posterior mean deviance of the individual data points in the inconsistency model was plotted against the corresponding posterior mean deviance in the consistency model to identify potential studies contributing to inconsistency (Additional file 1).

### Role of the funding source

Hoffmann-La Roche Ltd. funded this study. All authors had access to and the opportunity to review final study data, and are responsible for data interpretation and preparation of the report. All authors attest to study completeness, data accuracy, and data analysis, and all were responsible for the final decision to submit for publication.

## Results

**Fig. 1:**
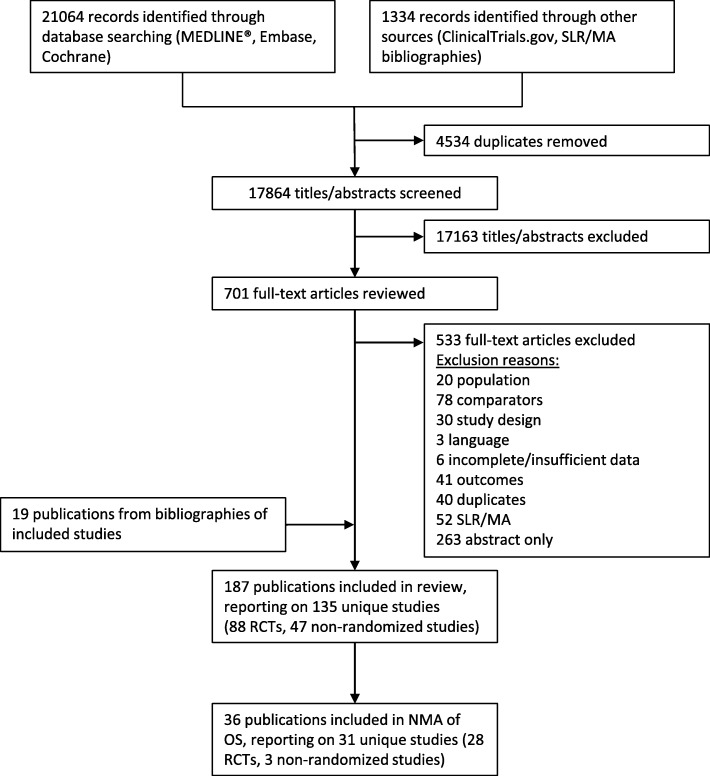
PRISMA flow diagram. *MA* meta-analysis, *NMA* network meta-analysis, *OS* overall survival, *PRISMA* Preferred Reporting Items for Systematic Reviews and Meta-Analyses, *RCT* randomized controlled trial, *SLR* systematic literature review

Figure 1 outlines the PRISMA diagram for the literature review. In total, 187 publications reporting on 135 unique studies met our inclusion criteria; however, only 36 publications reporting on 31 unique studies (28 RCTs and 3 NRS) reported OS and were included in our analyses (Table 1). Overall, the included studies had a low risk of bias (Additional file 1). Several RCTs allowed for crossover, but a lack of information prevented us from performing a sensitivity analysis (Additional file 1). Table 2 and the Additional file 1 summarize the 28 publications with OS results. Studies were conducted internationally, and median patient age ranged from 48 to 56 years. Approximately 15–100% of study patients were node-positive, 19–81% had HR+ tumors, and 45–100% had tumors ≥ 2 cm. Details of evidence networks and HRs used in analyses are available in the Additional file 1.

**Table 1:** Studies included in the cumulative NMA for overall survival

	2008	2010	2012	2014	2016
RCTs: 100% HER2+	–	–	–	–	ALTTO (Piccart-Gebhart 2016) [66]
	–	–	BCIRG 006 (Slamon 2011) [44]	BCIRG 006 (Slamon 2011) [44]	BCIRG 006 (Slamon 2015) [21]
	–	FNCLCC-PACS 04 (Spielmann 2009) [67]	FNCLCC-PACS 04 (Spielmann 2009) [67]	FNCLCC-PACS 04 (Spielmann 2009) [67]	FNCLCC-PACS 04 (Spielmann 2009) [67]
	–	–	–	–	HannaH (Jackisch 2016) [68]
	HERA (Smith 2007) [69]	HERA (Smith 2007) [69]	HERA (Gianni 2011) [19]	HERA (Goldhirsch 2013) [64]	HERA (Goldhirsch 2013) [64]
	–	–	–	–	HORG (Mavroudis 2015) [70]
	NCCTG N9831 and NSABP B-31 (Romond 2005) [18]	NCCTG N9831 and NSABP B-31 (Romond 2005) [18]	NCCTG N9831 andNSABP B-31 (Perez 2011) [71]	NCCTG N9831 and NSABP B-31 (Perez 2014) [51]	NCCTG N9831 and NSABP B-31 (Perez 2014) [51]
	–	–	–	NeoALTTO (de Azambuja 2014) [72]	NeoALTTO (de Azambuja 2014) [72]
	–	NOAH (Gianni 2010) [73]	NOAH (Gianni 2010) [73]	NOAH (Gianni 2014) [20]	NOAH (Gianni 2014) [20]
	–	–	–	–	NSABP B-41 (Robidoux 2016) [74]
	–	–	–	PHARE (Pivot 2013) [75]	PHARE (Pivot 2013) [75]
RCTs: HER2+ subgroups	–	–	–	BCIRG 001 (Mackey 2013) [76]	BCIRG 001 (Mackey 2013) [76]
	–	Boccardo 2010 [77]	Boccardo 2010 [77]	Boccardo 2010 [77]	Boccardo 2010 [77]
	–	–	BR9601 and NEAT (Earl 2012) [78]	BR9601 and NEAT (Earl 2012) [78]	BR9601 and NEAT (Earl 2012) [78]
	Colozza 2005 [79]	Colozza 2005 [79]	Colozza 2005 [79]	Colozza 2005 [79]	Colozza 2005 [79]
	–	–	–	–	Del Mastro 2015 [80]
	E1199 (Sparano 2008) [81]	E1199 (Sparano 2008) [81]	E1199 (Sparano 2008) [81]	E1199 (Sparano 2008) [81]	E1199 (Sparano 2008) [81]
	–	–	–	–	E2198 (Schneider 2015) [82]
	–	FinHer (Joensuu 2009) [83]	FinHer (Joensuu 2009) [83]	FinHer (Joensuu 2009) [83]	FinHer (Joensuu 2009) [83]
	–	–	–	FinXX (Joensuu 2014) [84]	FinXX (Joensuu 2014) [84]
	–	–	–	GeparTrio (von Minckwitz 2013) [85]	GeparTrio (von Minckwitz 2013) [85]
	GONO-MIG-1 (Del Mastro 2005) [86]	GONO-MIG-1 (Del Mastro 2005) [86]	GONO-MIG-1 (Del Mastro 2005) [86]	GONO-MIG-1 (Del Mastro 2005) [86]	GONO-MIG-1 (Del Mastro 2005) [86]
	Miles 1999 [87]	Miles 1999 [87]	Miles 1999 [87]	Miles 1999 [87]	Miles 1999 [87]
	–	–	–	Rocca 2014 [88]	Rocca 2014 [88]
	–	–	–	TEACH (Goss 2013) [89]	TEACH (Goss 2013) [89]
	–	–	UNICANCER-PACS-01 (Coudert 2012) [90]	UNICANCER-PACS-01 (Coudert 2012) [90]	UNICANCER-PACS-01 (Coudert 2012) [90]
Observational	–	–	Bayraktar 2012 [91]	Bayraktar 2012 [91]	Bayraktar 2012 [91]
	–	–	–	–	Gonzalez-Angulo 2015 [92]
	–	–	–	–	Seferina 2015 [93]

*HER2+* human epidermal growth factor receptor 2-positive, *NMA* network meta-analysis, *OS* overall survival, *RCT* randomized controlled trial

**Table 2:** Summary of study and patient characteristics from RCTs with overall survival results

Study (primary publication)	Treatments	Node name in network	Median follow-up (months)	*N* total	Median age (years)	Tumor Size > 2 cm (%)	Node + (%)	HR+ (%)	HER2+ (%)
RCTs with 100% HER2+ early breast cancer patients									
ALTTO (Piccart-Gebhart 2016) [66]	Anthracycline and/or taxane → trastuzumab IV (52 weeks)	AC-TH_52 weeks_	54	2097	51	49	51	57	100
	Anthracycline and/or taxane → lapatinib (52 weeks)	AC-TL_52 weeks_	54	2100	51	51	52	57	100
	Anthracycline and/or taxane → trastuzumab IV (12 weeks) → lapatinib (34 weeks)	AC-TL_34 weeks_-H_12 weeks_	54	2091	51	50	52	58	100
	Anthracycline and/or taxane → trastuzumab IV (52 weeks) + lapatinib (52 weeks)	AC-TL_52 weeks_-H_52 weeks_	54	2093	51	50	51	57	100
BCIRG 006 (Slamon 2011) [44]	Doxorubicin + cyclophosphamide → docetaxel	AC-T	65	1073	NR	59	71	54	100
	Doxorubicin + cyclophosphamide → docetaxel + trastuzumab IV (52 weeks)	AC-TH_52 weeks_	65	1074	NR	62	71	54	100
	Docetaxel + carboplatin + trastuzumab IV (52 weeks)	TCH_52 weeks_	65	1075	NR	59	72	54	100
FNCLCC-PACS 04 (Spielmann 2009) [67]	FEC or ED (epirubicin + docetaxel) → trastuzumab IV (52 weeks)	AC-TH_52 weeks_	47	260	48	59·1	100	58	100
	FEC or ED (epirubicin + docetaxel)	AC-T	47	268	49	50·6	100	61	100
HannaH (Jackisch 2016) [68]	Docetaxel → FEC + trastuzumab IV → trastuzumab IV (52 weeks)	AC-TH_52 weeks_	40·6	297	50	NR	79·1	49·8	100
	Docetaxel → FEC + trastuzumab SC → trastuzumab SC (52 weeks)	AC-TH_SC,52 weeks_	40·3	294	50	NR	75·8	52·4	100
HERA (Goldhirsch 2013) [64]	Anthracycline or taxane → trastuzumab IV (104 weeks)	AC-TH_104 weeks_	96	1700	NR	49·5	56·5	51·4	100
	Anthracycline or taxane → trastuzumab IV (52 weeks)	AC-TH_52 weeks_	96	1702	49	48·4	56·4	50·9	100
	Anthracycline or taxane	AC-T	96	1697	49	NR	NR	NR	100
HORG (Mavroudis 2015) [70]	FEC → docetaxel + trastuzumab IV (52 weeks)	AC-TH_52 weeks_	47	241	54	NR	74·7	64·7	100
	FEC → docetaxel + trastuzumab IV (26 weeks)	AC-TH_26 weeks_	51	240	56	NR	83·3	68·8	100
NCCTG N9831 and NSABP B-31 (Perez 2014) [51]	Doxorubicin + cyclophosphamide → paclitaxel	AC-T	99·6	2018	~ 50	59·2	92·6	54·8	100
	Doxorubicin + cyclophosphamide → paclitaxel + trastuzumab IV (52 weeks)	AC-TH_52 weeks_	100·8	2028	~ 50	61.8	93·4	54·7	100
NeoALTTO (de Azambuja 2014) [72]	Paclitaxel → FEC → lapatinib (52 weeks)	AC-TL_52 weeks_	45	154	50	100	> 16·2	51·9	100
	Paclitaxel → FEC → trastuzumab IV (52 weeks)	AC-TH_52 weeks_	45	149	49	100	> 15·4	50·3	100
	Paclitaxel + → FEC → lapatinib (52 weeks) + trastuzumab IV (52 weeks)	AC-TL_52 weeks_-H_52 weeks_	45	152	50	100	> 15·8	50·7	100
NOAH (Gianni 2014) [20]	Paclitaxel + doxorubicin → paclitaxel → CMF	AC-T	64·8	118	NR	NR	84	36	100
	[Paclitaxel + doxorubicin → paclitaxel → CMF → trastuzumab IV (52 weeks)	AC-TH_52 weeks_	64·8	117	NR	NR	86	36	100
NSABP B-41 (Robidoux 2013^b^) [94]	Doxorubicin + cyclophosphamide → paclitaxel → trastuzumab IV (52 weeks)	AC-TH_52 weeks_	22·8	181	NR	100	51	67	100
	Doxorubicin + cyclophosphamide → paclitaxel + lapatinib (12 weeks) → trastuzumab IV (34 weeks)	AC → T-L_12 weeks_-H_34 weeks_	22·8	174	NR	100	52	58	100
	Doxorubicin + cyclophosphamide → paclitaxel + lapatinib (12 weeks) + trastuzumab IV → trastuzumab IV (52 weeks)	AC → T-L_12 weeks_-H_52 weeks_	22·8	174	NR	100	49	62	100
PHARE (Pivot 2013) [75]	Anthracycline + taxane + trastuzumab IV → trastuzumab IV (52 weeks)	AC-TH_52 weeks_	42·5	1690	54	45.3	44·6	60·4	100
	Anthracycline + taxane + trastuzumab IV → trastuzumab IV (26 weeks)	AC-TH_26 weeks_	42·5	1690	55	47·6	45.3	61·5	100
RCTs with subgroup data of HER2+ early breast cancer patients									
BCIRG 001 (Mackey 2013) [76]	Docetaxel + doxorubicin + cyclophosphamide	AC-T	124	745	49	61	100	76·1	21
	Fluorouracil + doxorubicin + cyclophosphamide	AC	123	746	49	57	100	75·7	22
Boccardo 2010 [77]	Epirubicin → cyclophosphamide + methotrexate + fluorouracil	AC	102	122	53·0	47·5	100	79·5	31·1
	Paclitaxel → epirubicin + vinorelbine	T → AV	102	122	54·5	64.8	100	79·5	28·7
BR9601 and NEAT (Earl 2012) [78]	Epirubicin → CMF	AC	88·8	1189	NR	56	72	≥59	21
	Cyclophosphamide + methotrexate + fluorouracil	CMF	88·8	1202	NR	56	72	≥59	20
Colozza 2005 [79]	Cyclophosphamide + methotrexate + fluorouracil	CMF	96	133	NR	51	80	≥63	69
	Epirubicin	E	96	133	NR	48	78	≥63	77
Del Mastro 2015 [80]	Epirubicin + cyclophosphamide → paclitaxel (q3w)	AC-T	84	545	51	48	100	77	23
	FEC → paclitaxel (q3w)	AC-T	84	544	53	52	100	81	24
	Epirubicin + cyclophosphamide → paclitaxel (q2w)	Dose dense	84	502	53	48	100	81	21
	FEC → paclitaxel (q2w)	Dose dense	84	500	51	49	100	80	24
E1199 (Sparano 2015^c^) [95]	Doxorubicin + cyclophosphamide → paclitaxel (weekly)	Dose dense	145·2	1232	51	63.8	87.8	70·2	19·2
	Doxorubicin + cyclophosphamide → paclitaxel (q3w)	AC-T	145·2	1253	51	60.7	87.8	71·0	20·6
E2198 (Schneider 2015) [82]	Paclitaxel + trastuzumab IV (10 weeks) → doxorubicin + cyclophosphamide	AC-TH_9–10 weeks_	77	115	49	NR	100	60	53
	Paclitaxel + trastuzumab IV (10 weeks) → doxorubicin + cyclophosphamide + trastuzumab IV (52 weeks total)	AC-TH_52 weeks_	77	112	48	NR	100	63	
FinHer (Joensuu 2009) [83]	Docetaxel → FEC	AC-T	62	58	49·9	70	78	≥44	100^a^
	Docetaxel + trastuzumab IV (9 weeks) → FEC	AC-TH_9–10 weeks_	62	54	51·4	59	90	≥50	100^a^
	Vinorelbine → FEC	AC-V	62	58	49·9	70	78	≥44	100^a^
	Vinorelbine + trastuzumab IV (9 weeks) → FEC	AC-VH_9 weeks_	62	62	51·4	59	90	≥50	100^a^
FinXX (Joensuu 2014) [84]	Capecitabine + docetaxel → cyclophosphamide + epirubicin + capecitabine → trastuzumab IV (52 weeks); or docetaxel → FEC → trastuzumab IV (52 weeks)	AC-TH_52 weeks_	80·4	176	52·2	NR	84·6	60·2	100^a^
	Capecitabine + docetaxel → cyclophosphamide + epirubicin + capecitabine; or docetaxel → FEC	AC-T	80·4	108	50·5	NR	81·4	51·9	100^a^
GeparTrio (von Minckwitz 2013) [85]	Docetaxel + doxorubicin + cyclophosphamide → vinorelbine + capecitabine (in non-responders)	AC-T → VX	62	987	NR	NR	54·7	65·6	29·1
	Docetaxel + doxorubicin + cyclophosphamide	AC-T	62	1025	NR	NR	55·3	63·2	30·5
GONO-MIG-1 (Del Mastro 2005) [86]	FEC (q3w)	AC	80·4	53	54	NR	61·2	≥27·2	100^a^
	FEC (q2w)	Dose dense	80·4	50	54	NR	61·2	≥27·2	100^a^
Miles 1999 [87]	Cyclophosphamide + methotrexate + fluorouracil	CMF	159·6	129	NR	78	100	≥77·5	30
	No treatment	No Tx	159.6	145	NR	78	100	≥77·5	30
Rocca 2014 [88]	Epirubicin → CMF, or CMF → epirubicin	AC	69	545	53	NR	48·3	≥64·0	34·1
	Cyclophosphamide + methotrexate + fluorouracil	CMF	69	160	51	NR	47·5	≥56·9	30·0
TEACH (Goss 2013) [89]	Lapatinib (52 weeks) + anthracycline/taxane	AC-TL_52 weeks_	47.4	1571	51	NR	54	59	78
	Placebo (52 weeks) + anthracycline/taxane	AC-T	48·3	1576	52	NR	54	59	80
UNICANCER-PACS01 (Coudert 2012) [90]	FEC	AC	92·8	996	NR	57·8	100	78	9·4
	FEC → docetaxel	AC-T	92·8	1003	NR				
Non-randomized studies with 100% HER2+ early breast cancer patients									
Bayraktar 2012 [91]	Paclitaxel + trastuzumab → FEC + trastuzumab (52 weeks)	AC-TH_52 weeks_	29	235	49	NR	81·5	≥53·8	100
	Docetaxel + carboplatin + trastuzumab (52 weeks)	TCH_52 weeks_	18	65	53	NR	80.0	≥55·6	100
Gonzalez-Angulo 2015 [92]	Adjuvant trastuzumab: paclitaxel + trastuzumab → FEC + trastuzumab (52 weeks)	AC-TH_52 weeks_	45	480	~ 50	NR	19·4	61·1	100
	No adjuvant trastuzumab: paclitaxel + trastuzumab (26 weeks) → FEC	AC-TH_26 weeks_	45	109	~ 50	NR	15·6	53·2	100
Seferina 2015 [93]	Anthracycline/taxane-containing chemotherapy + trastuzumab (52 weeks)	AC-TH_52 weeks_	60	230	51	> 56	56·0	62	100
	Endocrine therapy and radiotherapy, no chemotherapy	No chemo	60	246	65	NR	NR	NR	100

Data were extracted from the most recent full-text publications, when available

^a^Patient characteristics were available for the HER2+ subgroup population. Therefore, the percentage of HER2+ patients in the HER2+ subgroup is 100%, even though it is a non-randomized subset of the RCT

^b^Patient characteristics for NSABP B-41 were extracted from Robidoux 2013; [94] OS results were extracted from Robidoux 2016. [74]

^c^Patient characteristics for E1199 were extracted from Sparano 2015; [95] OS results were extracted from Sparano 2008. [81]

*AC* anthracycline (doxorubicin, epirubicin) + cyclophosphamide, *AV* anthracycline + vinorelbine, *CMF* cyclophosphamide + methotrexate + fluorouracil, *Dose dense* AC → T, or AC, either weekly or biweekly, *E* epirubicin, *ED* epirubicin + docetaxel, *ER+* estrogen receptor-positive, *FEC* fluorouracil + epirubicin + cyclophosphamide, *H* Herceptin® intravenous (IV), *HER2+* human epidermal growth factor receptor 2-positive, *HR+* hormone receptor-positive, *H*_*SC*_ Herceptin® subcutaneous (SC), *IV* intravenous, *L* lapatinib, *No Chemo* no chemotherapy (includes endocrine therapy and radiotherapy), *No Tx* no treatment, *NR* not reported, *OS* overall survival, *PBO* placebo, *PgR+* progesterone receptor-positive, *q2w* once every 2 weeks, *q3w* once every 3 weeks, *RCT* randomized controlled trial, *T* taxane (docetaxel, paclitaxel), *TCH* docetaxel + carboplatin + Herceptin® IV, *V* vinorelbine, *X* capecitabine

**Fig. 2:**
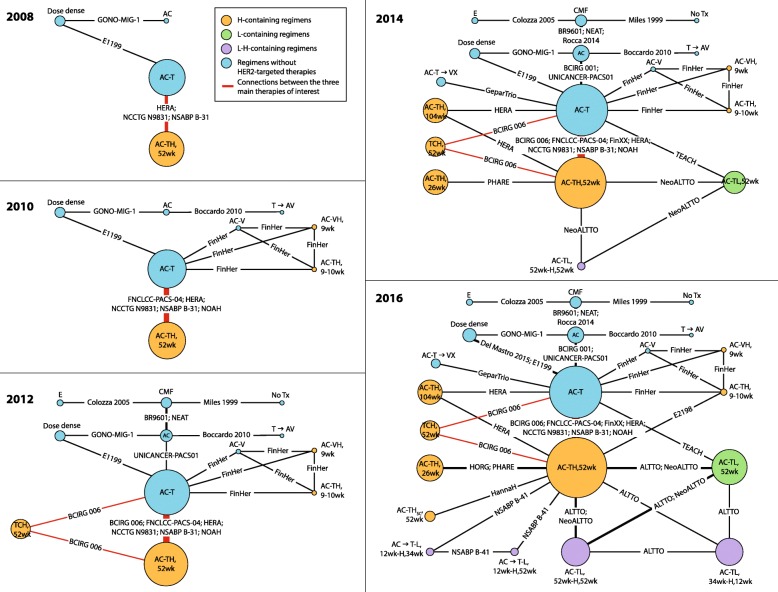
Cumulative NMA evidence networks for overall survival: reference case including RCTs with 100% HER2+ patients and HER2+ subgroups. *AC* anthracycline (doxorubicin, epirubicin) + cyclophosphamide, *Dose dense* AC → T, or AC, either weekly or biweekly, *E* epirubicin, *H* Herceptin® intravenous (IV), *HSC* Herceptin® subcutaneous (SC), *L* lapatinib, *NMA* network meta-analysis, *No Tx* no treatment, *T* taxane (docetaxel, paclitaxel), *TCH* docetaxel + carboplatin + Herceptin® IV, *V* vinorelbine, *X* capecitabine

**Fig. 3:**
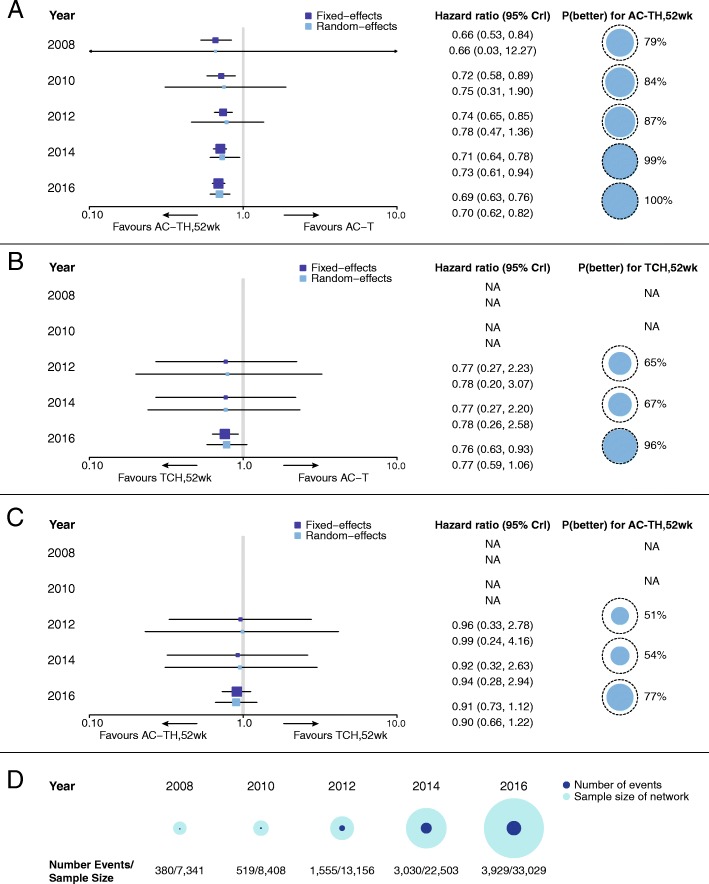
Cumulative NMA results for overall survival: reference case including RCTs with 100% HER2+ patients and HER2+ subgroups. **a** AC-TH_52 weeks_ vs. AC-T, **b** TCH_52 weeks_ vs. AC-T, **c** AC-TH_52 weeks_ vs. TCH_52 weeks_, and **d** corresponding sample sizes and number of events. Boxes on the forest plots represent the hazard ratios, with 95% CrIs shown by the horizontal lines. The size of each box is based on the precision of each effect estimate, calculated as the inverse of the variance (precision = 1/SE^2^, where SE is a standard error). The *x* axis is presented in log-format. Probability better values are based on the random effects model. The dashed circle represents the maximum p(better) value that is possible: 100%. *AC* anthracycline (doxorubicin, epirubicin) + cyclophosphamide, *CrI* credible interval, *H* Herceptin® intravenous (IV), *NA* not available, *OS* overall survival, *P(better)* probability better, *T* taxane (docetaxel, paclitaxel), *TCH* docetaxel + carboplatin + Herceptin® IV

Figure 2 shows the reference case cNMA evidence networks (interactive figure available online: https://goo.gl/ppkLrG). The final evidence network in 2016 includes 21 nodes connected by 28 RCTs (26 publications). Data from head-to-head trials were available for 31 pairwise comparisons in the network with single studies informing 24 comparisons. In total, 7341 patients (380 deaths) were included in the 2008 evidence network. By 2016, 33,029 patients (3929 deaths) were included. Based on results from the reference case cNMA RE model (Fig. 3), for the pairwise comparison of AC-TH_52 weeks_ vs. AC-T, evidence in 2008 demonstrated an OS advantage for H/chemotherapy compared with chemotherapy alone (HR 0.66, 95% CrI 0.03–12.27). The corresponding probability of AC-TH_52 weeks_ being better than AC-T in 2008 was 79% (standard deviation [SD] 41%). The certainty of this survival benefit strengthened over time, with an OS advantage for AC-TH_52 weeks_ relative to AC-T in 2016 (HR 0.70, 0.62–0.82), and a p(better) value of 100% (SD 2%).

For the pairwise comparison of TCH_52 weeks_ vs. AC-T, no evidence was available for 2008 or 2010. Initial published data in 2012 demonstrated no significant difference in OS for H/chemotherapy compared with chemotherapy alone (HR 0.78, 95% CrI 0.20–3.07). Over time, the precision around the OS estimate improved, showing an OS advantage for H/chemotherapy compared with chemotherapy alone in 2016 (HR 0.77, 0.59–1.06). The probability of TCH_52 weeks_ being better than AC-T in 2012 was 65% (SD 48%), and this increased to 96% (SD 21%) in 2016, due to the addition of studies to indirect comparisons.

For the pairwise comparison of AC-TH_52 weeks_ vs. TCH_52 weeks_, both H/chemotherapy regimens showed a similar OS advantage, and the precision around these effect estimates improved over time. The effect estimate in 2016 showed a slight advantage for AC-TH_52 weeks_ compared with TCH_52 weeks_, (HR 0.90, 95% CrI 0.66–1.22; p[better] 77%, SD 42%).

Model fit statistics were favorable for both the FE and RE models (Table 3). The FE model was preferred for earlier time points when the evidence networks were largely composed of single-study connections. The RE model was preferred for later time points when the network incorporated more multi-study connections, and the RE model heterogeneity was lowest in 2016. An assessment of inconsistency for the 2016 reference case analysis did not identify any concerns regarding inconsistency between direct and indirect evidence (Additional file 1).

**Table 3:** Model fit statistics

Year	Fixed effects	Random effects
Reference case: RCTs with 100% HER2+ patients and HER2+ subgroups		
2008	DIC = 1.69 TotResDev = 2.99 vs. 4	DIC = 3.19 TotResDev = 3.74 vs. 4 Heterogeneity SD (95% CI) = 0.68 (0.01 to 4.55)
2010	DIC = 17 TotResDev = 10.63 vs. 10	DIC = 17.96 TotResDev = 9.66 vs. 10 Heterogeneity SD (95% CI) = 0.37 (0.02 to 2.41)
2012	DIC = 28.67 TotResDev = 19.63 vs. 16	DIC = 26.29 TotResDev = 14.64 vs. 16 Heterogeneity SD (95% CI) = 0.31 (0.05 to 1.16)
2014	DIC = 25.24 TotResDev = 25.67 vs. 25	DIC = 25.76 TotResDev = 23.27 vs. 25 Heterogeneity SD (95% CI) = 0.12 (0.01 to 0.43)
2016	DIC = 28.17 TotResDev = 33.94 vs. 34	DIC = 28.17 TotResDev = 32.07 vs. 34 Heterogeneity SD (95% CI) = 0.08 (0.01 to 0.27)

*CI* confidence interval, *DIC* deviance information criterion, *HER2+* human epidermal growth factor receptor 2-positive, *RCT* randomized controlled trial, *SD* standard deviation, *TotResDev* total residual deviance

Cumulative NMA results from sensitivity analyses are provided in the Additional file 1. In sensitivity analysis #1 (only RCTs with 100% HER2+ patients), cNMA results showed an OS advantage for patients who received H/chemotherapy compared with chemotherapy alone, and the presence of anthracycline in the chemotherapy regimen did not significantly affect OS results. In sensitivity analysis #2 (naïve pooling of RCTs and NRS), cNMA results for the pairwise comparison of AC-TH_52 weeks_ vs. AC-T aligned with the reference case results. For the pairwise comparison of TCH_52 weeks_ vs. AC-T, initial evidence in 2012 demonstrated no significant difference in OS for H/chemotherapy compared with chemotherapy alone, but by 2016, there was a shift to an OS advantage for patients who received TCH_52 weeks_, and the precision around the estimate improved. For the pairwise comparison of AC-TH_52 weeks_ vs. TCH_52 weeks_, initial evidence in 2012 suggested an OS advantage for AC-TH_52 weeks_ (although not significant). The precision around the effect estimates for this comparison improved over time, with a shift towards both H/chemotherapy regimens showing a similar OS advantage in 2016. Results from the reference case cNMA were also supported by a sensitivity analysis using whole survival curves.

**Fig. 4:**
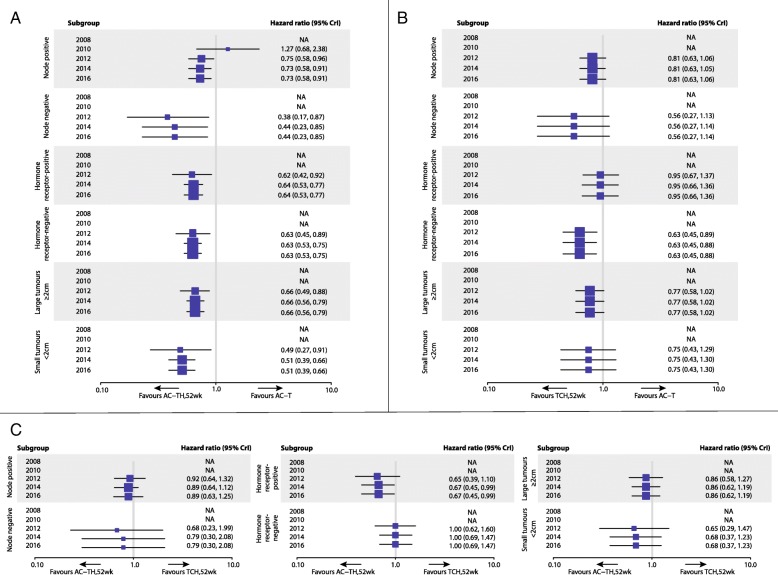
Cumulative NMA overall survival results of subgroup analyses for the pairwise comparisons **a** AC-TH_52 weeks_ vs. AC-T, **b** TCH_52 weeks_ vs. AC-T, and **c** AC-TH_52 weeks_ vs. TCH_52 weeks_. Boxes on the forest plots represent the hazard ratios, with 95% CrIs shown by the horizontal lines. The size of each box is based on the precision of each effect estimate. The *x* axis is presented in log format. *AC* anthracycline (doxorubicin, epirubicin) + cyclophosphamide, *CrI* credible interval, *H* Herceptin® intravenous (IV), *NA* not available, *T* taxane (docetaxel, paclitaxel), *TCH* docetaxel + carboplatin + Herceptin® IV

We conducted subgroup analyses based on nodal status, HR status, and tumor size; a subgroup analysis of neoadjuvant versus adjuvant therapies was not feasible because of insufficient information (Additional file 1). Due to many single-study connections in the subgroup analyses, we have only presented results from the FE model (Fig. 4). Our results show that the addition of H to chemotherapy provides an OS advantage for all analyzed subgroups. For the node-negative and small tumors (< 2 cm) subgroups, a slightly greater OS advantage was provided by H/anthracycline-containing chemotherapy compared with H/non-anthracycline-containing chemotherapy (2016 HR 0.79, 95% CrI 0.30–2.08; 2016 HR 0.68, 0.37–1.23, respectively), although there was reduced precision in the effect estimates compared with the node-positive (2016 HR 0.89, 0.63–1.25) and large tumors (≥ 2 cm) (2016 HR 0.86, 0.62–1.19) subgroups, respectively. HR− patients showed similar OS effects from either an anthracycline- or non-anthracycline-containing regimen with H (2016 HR 1.00, 0.69–1.47), whereas HR+ patients received greater OS benefit from H/anthracycline-containing chemotherapy (2016 HR 0.67, 0.45–0.99).

## Discussion

Our cNMA evaluated the OS advantage conferred by the addition of H to standard chemotherapy in HER2+ EBC, compared the two most widely used H/chemotherapy regimens, and assessed how the evidence evolved over time. Initial evidence from the reference case analysis consistently demonstrated an OS advantage for patients who received standard of care treatment with 52 weeks of H/chemotherapy compared with chemotherapy alone. Over time, the precision around the OS estimates improved and the certainty in the survival benefit strengthened. A comparison of H/anthracycline versus H/non-anthracycline-containing chemotherapy regimens showed that both regimens provided a similar OS advantage for HER2+ EBC patients, and the precision around the effect estimates for this comparison improved over time. Over 2.2 million HER2+ BC patients have been treated with H, including nearly 17,000 in the context of a clinical trial [39]. This, combined with data presented herein, clearly support originator trastuzumab as the established standard of care in the HER2+ EBC setting. In addition, a survival benefit was seen for all analyzed patient subgroups who received 52 weeks of H/chemotherapy.

Although H/chemotherapy demonstrated a survival benefit in all node-positive and node-negative subgroup analyses, the limited availability of published data for the node negative subgroup resulted in reduced precision of effect estimates. Node-positive disease is associated with higher risk of relapse [39], so more intensive regimens are commonly used to treat these patients [4, 40, 41], which may show a greater magnitude of effect. Few node-negative patients were included in the RCTs evaluated. Although the recent adjuvant paclitaxel and trastuzumab (APT) trial showed that an adjuvant H/paclitaxel regimen is beneficial to node-negative patients, and it is less toxic than a traditional adjuvant regimen, this trial was not included in our analyses due to its single-arm design and lack of OS data [42].

Results from the HR subgroups showed that HR− patients received an OS benefit from H regardless of whether an anthracycline- or non-anthracycline-containing regimen was administered, whereas HR+ patients received OS benefit from an anthracycline-containing regimen with H. This difference may be due to coamplification of topoisomerase II alpha (TOP2A), which occurs in about a third of HER2+ cancers, and results in increased anthracycline sensitivity, longer progression-free survival, and improved OS [43–45]. HR− patients may experience more relapse events and deaths in the first 5 to 8 years of follow-up compared with HR+ patients [4, 46–49]. By 8 years of follow-up, the incidence of relapse is approximately equal in both subgroups. After 8 years, more relapse events were observed in the HR+ subgroup, which could be due to the overexpression of hormone receptors and activation of additional cell signaling pathways [4, 48, 49]. Increased anthracycline sensitivity in the HR+ subgroup could be contributing to the beneficial OS effect by targeting estrogen signaling pathways. HR+ patients are likely also receiving endocrine therapy, which may contribute to the lower relapse incidence observed at earlier stages in this subgroup.

The broader use of mammographic screening and earlier diagnosis of EBC has resulted in an increased incidence of small tumors [50]. Our cNMA subgroup results for patients with small tumors (< 2 cm) show a slightly greater OS advantage was provided by H/anthracycline-containing chemotherapy compared with H/non-anthracycline-containing chemotherapy. Precision improvement was only seen with H/anthracycline-containing chemotherapy, due to the addition of clinical evidence from the North Central Cancer Treatment Group (NCCTG) N9831 Intergroup trial and National Surgical Adjuvant Breast and Bowel Project (NSABP) B-31 joint analysis [51]. Published data were only available for a tumor size threshold of 2 cm, but future work could investigate tumors of < 1 cm to confirm if H could also benefit these patients. The APT trial investigated patients with tumors ≤ 3 cm and suggested that an adjuvant H/paclitaxel regimen may be preferred for these patients; however, an H/anthracycline-containing comparator was not included [42]. Conversely, results from our cNMA suggest that patients with tumors < 2 cm may benefit more from H/anthracycline-containing chemotherapy; however, only three trials were included in this analysis, so results should be interpreted with caution. In line with the APT trial, current guidelines recommend an adjuvant therapy of H/paclitaxel for small, HER2+, and node-negative tumors [7, 52]. In comparison, results from our cNMA subgroup analyses suggest that patients with small tumors (< 2 cm) and patients with node-negative tumors may benefit from an H/anthracycline-containing chemotherapy regimen compared with an H/non-anthracycline-containing chemotherapy regimen. This discrepancy shows the need for additional trials to investigate H/anthracycline-containing versus H/non-anthracycline-containing adjuvant therapies in this subpopulation.

The cNMA subgroup results provide evidence for the differential use of anthracycline- versus non-anthracycline-containing regimens with H that may preferentially benefit certain subgroups. Although the HR+ subgroup appeared to show greater relative improvements in OS with anthracycline-containing regimens, the HR− subgroup showed a similar benefit with either chemotherapy regimen. Therefore, HR− patients could avoid the cardiotoxic effects caused by anthracyclines by choosing a non-anthracycline-containing regimen. Similarly, node-negative patients could avoid anthracycline cardiotoxicity, as H/non-anthracycline-containing chemotherapy appeared to be effective for this subgroup, although with reduced precision.

These subgroup analyses align with a recent meta-analysis that demonstrated an OS benefit for HER2+ EBC patients with small (≤ 2 cm), HR+ or HR− tumors who received H with their treatment regimens [53]. A potential limitation of our subgroup analyses is the heterogeneity caused by limited available data and small sample sizes. An imbalance in the weight of our subgroup results is due to an imbalance in the distribution of subgroups in studies. For instance, most RCTs in EBC were not designed solely for patients with node negative disease or small tumors [5]. Therefore, although our results support current clinical practice, subgroup results should be interpreted with caution [5]. Future studies should further assess these subgroups to help direct the neoadjuvant/adjuvant treatment approach.

The presence of heterogeneity in the studies and the structure of the evidence networks limited our ability to adjust for various patient and study characteristics. However, we have accounted for heterogeneity and inconsistency using best practices that are consistent with those employed by HTA bodies such as NICE and CADTH [54–57]. A limited number of high-quality NRS were identified, which restricted the methods used to combine evidence from RCTs and NRS. However, this lack of NRS is not expected to alter findings; NRS are often associated with more favorable estimates than RCTs, so our estimates are likely conservative. The lack of NRS also likely improves the validity of our findings, because inclusion of such studies in an NMA often introduces bias [25, 32, 33].

Despite these limitations, the SLR underpinning the cNMA is the most up-to-date and comprehensive review currently available for the treatment of women with HER2+ EBC [58–62]. The SLR search spanned 26 years and identified over 17,800 unique records, demonstrating the vast amount of evidence available in this area of oncology. This study adheres to best practices for the conduct of NMA [54, 55] and to PRISMA reporting guidelines (Additional file 1) [54]. Thorough sensitivity and subgroup analyses were conducted, adding strength and validity to the findings. Specifically, the inclusion of RCTs and NRS in a sensitivity analysis provided additional evidence to strengthen the comparisons. The results from the reference case cNMA were also supported by a sensitivity analysis using whole survival curves rather than HRs.

The 11-year follow-up results from the HERA trial were published after the SLR end date (January 19, 2017) [63], and therefore were not identified by our search. Our current analyses include OS results from the 8-year median follow-up [64]. The 11-year OS results show a similar, yet slightly stronger advantage for treatment with 52 weeks of H compared with observation, which is in alignment with our findings. The OS results for HR+ and HR− subgroups at 11 years [63] also align with our subgroup results. Results from the phase III APHINITY trial were also recently published [65]. This trial investigates whether the addition of pertuzumab (Perjeta®) to adjuvant H/chemotherapy improves patient outcomes compared with H/chemotherapy alone. At a 45·4-month median follow-up, HER2+ EBC patients receiving dual HER2-targeted therapy showed a reduced risk of BC recurrence or death compared with patients receiving H/chemotherapy alone, and this effect was most detectable among higher-risk patients with node-positive or HR− disease [65]. It would be worthwhile to incorporate data from these recent trials into a future cNMA. The improved precision in OS estimates that we see for regimens including H may be due, in part, to the establishment of H as standard of care therapy for HER2+ EBC patients, thus reinforcing the probability of H/chemotherapy being better than chemotherapy alone. Advances in earlier diagnosis and better disease management are likely also contributing to improved efficacy.

## Conclusions

The current SLR/cNMA represents the most comprehensive study to date of treatments for HER2+ EBC. It is uncommon to review the totality of a product’s clinical evidence at various time points and particularly over a prolonged timeframe. Initial evidence demonstrated an OS advantage for H/chemotherapy compared with chemotherapy alone in HER2+ EBC patients. The certainty of this survival benefit strengthened over time, as evidenced by the cNMA results. These findings demonstrate why H/chemotherapy is the established standard of care in HER2+ EBC, and support the decision to allow early patient access to H, as the benefits of treatment far outweigh the risk of waiting for more precise information to be published. Building on this legacy, H, as a subcutaneous injection, continues to provide strong benefits to these patients.

## Additional file

Additional file 1: Supplementary Methods and Results. (DOCX 3954 kb)

## Abbreviations

BC: Breast cancer
cNMA: Cumulative network meta-analysis
EBC: Early breast cancer
H: Herceptin®
HER2+: Human epidermal growth factor receptor 2-positive.
MBC: Metastatic breast cancer
NRS: Non-randomized study
OS: Overall survival
RCT: Randomized controlled trial
SLR: Systematic literature review
