# Supplementary material for: Herceptin® (trastuzumab) in HER2-positive early breast cancer: a systematic review and cumulative network meta-analysis
Source: Syst Rev. 2018 Nov 14;7:191. doi: 10.1186/s13643-018-0854-y (PMC6237027; doi:10.1186/s13643-018-0854-y)
Supplement: Supplementary file 1 — Supplementary Methods and Results. (DOCX 3954 kb) [file 13643_2018_854_MOESM1_ESM.docx]

# Additional file

**Herceptin® (trastuzumab) in HER2-positive Early Breast Cancer: A Systematic Review and Cumulative Network Meta-Analysis**

Wilson, F.R., Coombes, M.E., Brezden-Masley, C., Yurchenko, M., Wylie, Q., Douma, R., Varu, A., Hutton, B., Skidmore, B., Cameron, C.

Search Strategy

HER2 Breast Cancer

Final Strategies – OVID, Cochrane

2016 Oct 19

OVID Multifile

Database: Embase <1988 to 2016 Week 42>, Epub Ahead of Print, In-Process & Other Non-Indexed Citations, Ovid MEDLINE(R) Daily and Ovid MEDLINE(R) <1946 to Present>

Search Strategy:

--------------------------------------------------------------------------------

1 exp Breast Neoplasms/ (647870)

2 ((breast$1 or mamma or mammary) adj3 (adenocarcinoma* or cancer* or carcinoma* or neoplasm* or tumour* or tumor*)).tw,kw. (624530)

3 1 or 2 [BREAST CANCER] (774147)

4 Receptor, ErbB-2/ (51944)

5 ERBB2 protein, human.nm. (6540)

6 (ErbB2 or "ErbB 2" or HER2* or "HER 2*" or "c-ErbB2" or "C-ErbB 2").tw,kw. (84076)

7 ((oncoprotein* or onco-protein* or protein* or receptor*) adj1 (neu or neuregulin)).tw,kw. (2743)

8 (CD340 adj1 antigen?).tw,kw. (0)

9 ("p185(c-neu)" or p185erbB).tw,kw. (95)

10 neu proto-oncogene protein*.tw,kw. (4)

11 metastatic lymph node gene 19 protein*.tw,kw. (0)

12 "luminal b".tw,kw. (3500)

13 luminal subtype*.tw,kw. (711)

14 (human adj1 "epidermal growth factor receptor 2").tw,kw. (10097)

15 Receptor, Epidermal Growth Factor/ (82682)

16 limit 15 to yr="2006-2007" (7236)

17 or/4-14,16 (106066)

18 3 and 17 [HER2 BREAST CANCER] (70383)

19 Combined Modality Therapy/ (202482)

20 limit 19 to yr="1984-1991" (40042)

21 Chemotherapy, Adjuvant/ (74848)

22 ((chemotherap* or chemo-therap*) adj5 (adjuvant* or adjuvent* or neoadjuvant* or neo-adjuvant* or neoadjuvent* or neo-adjuvent* or adjunct* or neoadjunct* or neo-adjunct*)).tw,kw. (90712)

23 ((drug therap* or pharmacothera* or pharmaco-therap*) adj5 (adjuvant* or adjuvent* or neoadjuvant* or neo-adjuvant* or neoadjuvent* or neo-adjuvent* or adjunct* or neoadjunct* or neo-adjunct*)).tw,kw. (1733)

24 exp Breast Neoplasms/dt (126897)

25 exp Antineoplastic Agents/ (2507071)

26 ("anti-HER2" or "anti-HER-2").tw,kw. (5057)

27 ((HER2 or "HER 2") adj1 block*).tw,kw. (471)

28 ((HER2 or "HER 2") adj1 antagoni*).tw,kw. (65)

29 Receptor, ErbB-2/ai [Antagonists & Inhibitors] (1848)

30 Antineoplastic Combined Chemotherapy Protocols/ (313774)

31 Trastuzumab/ (34858)

32 (herceptin* or trastuzumab* or TZM).tw,kw. (27821)

33 (ado-trastuzumab* or kadcyla or pro 132365 or pro132365 or "t dm 1" or "t dm1" or tmab mcc dm1).tw,kw. (1200)

34 trastuzumab.rn. (28831)

35 exp Cyclophosphamide/ (205396)

36 (alkyroxan or b 518 or b518 or carloxan or ciclofosfamida or ciclolen or cicloxal or clafen or cyclo-cell or cycloblastin or cycloblastine or cyclofosamide or cyclofosfamid or cyclofosfamide or cyclophar or cyclophosphamide or cyclophosphamides or cyclophosphan or cyclophosphane or cyclostin or cycloxan or cyphos or cytophosphan or cytophosphane or cytoxan or endocyclophosphate or endoxan or endoxana or enduxan or genoxal or ledoxan or ledoxina or mitoxan or neosan or neosar or noristan or nsc 26271 or nsc26271 or nsc 2671 or nsc2671 or procytox or procytoxide or semdoxan or sendoxan or syklofosfamid).tw,kw. (97880)

37 cyclophosphamide.rn. (187679)

38 Doxorubicin/ (182708)

39 (adriablastin? or adriacin or adriamicin? or adriamycin? or adriblastin? or adrim or adrimedac or adrubicin or amminac or caelix or caelyx or carcinocin or "dox sl" or doxil or DOXO-cell or doxolem or "doxor lyo" or doxorubicin? or doxotec or evacet or farmiblastina or "fi 106" or fi106 or ifadox or lipodox or "mcc 465" or mcc465 or myocet? or nsc 123127 or nsc123127 or onkodox or rastocin? or resmycin or ribodoxo or rubex or rubidox or sarcodoxome or "tlc d 99").tw,kw. (109652)

40 doxorubicin.rn. (171278)

41 Epirubicin/ (29051)

42 (4'-Epi-Adriamycin or 4'-Epi-Doxorubicin or 4'-Epi-DXR or 4'-Epiadriamycin or 4'-Epidoxorubicin or binarin or ellence or EPI-cell or epiadriamycin or epidoxo or epidoxorubicin or epidx or epifil or epilem or epirubicin or farmorrubicina or farmorubicin? or IMI-28 or NSC-256942 or NSC256942 or pharmorubicin? or pidorubicin).tw,kw. (13010)

43 epirubicin.rn. (27584)

44 exp Fluorouracil/ (145927)

45 (5-FU or 5FU or 5-fluorouracil or 5fluorouracil or 5-fluoruracil or 5fluoruracil or accusite or actino-hermal or adrucil or carac or effluderm or efudex or efudix or efurix or f6627 or fivoflu or fluoro-uracile or fluoroblastin or fluoroplex or fluorouracil or fluoruracil or fluouracil or fluracedyl or flurodex or fluracil or fluracilium or fluril or fluroblastin or flurouracil or fluoxan or haemato-fu or ifacil or neofluor or nsc 18913 or nsc18913 or nsc 19893 or nsc19893 or oncofu or onkofluor or ribofluor or uflahex or utoral or verrumal).tw,kw. (85471)

46 Fluorouracil.rn. (132472)

47 Capecitabine/ (25508)

48 (apecitab or ecansya or capecitabine or xeloda).tw,kw. (15308)

49 capecitabine.rn. (21653)

50 methotrexate/ (162708)

51 (amethopterine or abitrexate or amethopterin or amethopterine or ametopterine or antifolan or biotrexate or canceren or cl 14377 or cl4377 or emtexate or emthexat or emthexate or emtrexate or enthexate or farmitrexat or farmitrexate or farmotrex or folex or ifamet or imeth or intradose MTX or lantarel or ledertrexate or maxtrex or metex or methoblastin or methohexate or methotrate or methotrexat or methotrexate or methotrexato or methoxtrexate or methrotrexate or methylaminopterin or methylaminopterine or meticil or metoject or metothrexate or metotrexat or metotrexate or metotrexin or metrex or mexate or mpi 5004 or mpi5004 or neotrexate or novatrex or nsc 740 or nsc740 or otrexup or rasuvo or reumatrex or rheumatrex or texate or texate-t or texorate or trexall or xaken or zexate).tw,kw. (82437)

52 methotrexate.rn. (146922)

53 gemcitabine.tw,kw. (32864)

54 (gemcite or gemzar or ly 188011 or ly188011).tw,kw. (2207)

55 Carboplatin/ (64243)

56 (blastocarb or boplatex or carboplat or carboplatin or carboplatino or carbosin or carbotec or carplan or CBDCA or cycloplatin or erbakar or ercar or ifacap or kemocarb or nsc 241240 or nsc241240 or oncocarbin or paraplatin or paraplatine).tw,kw. (33222)

57 carboplatin.rn. (58198)

58 Cisplatin/ (185363)

59 (abiplatin or biocisplatinum or biocysplatinum or blastolem or briplatin or cis ddp or cis diamine dichloroplatinum or cis diaminechloroplatinum or cis diaminedichloroplatinum or cis diammine dichloroplatinum or cis diamminedichloroplatinum or cis dichloridiammineplatinum or cis dichloroadiamine platinum or cis dichlorodiamine platinum or cis dichlorodiamineplatinum or cis dichlorodiammine platinum or cis dichlorodiammineplatinum or cis platinous diamino dichloride or cis platinum or cisplatin or cisplatine or cisplatino or cisplatinum or cisplatyl or citoplatino or cytoplatin or cytosplat).tw,kw. (124715)

60 (diamine dichloroplatinum or diaminodichloroplatinum or diamminedichloroplatinum or dichlorodiamine platinum or dichlorodiammineplatinum or docistin or elvecis or kemoplat or lederplatin or lipoplatin or mpi 5010 or mpi5010 or neoplatin or niyaplat or nk 801 or nk801 or noveldexis or nsc 119875 or nsc119875 or platamine or platiblastin or platidiam or platimine or platinex or platinil or platinol or platinoxan or platiran or platistil or platistin or platosin or randa or romcis or sicatem or "spi 077" or tecnoplatin).tw,kw. (6592)

61 (platinum* adj1 (diaminodichloride or diamino dichloride or diamine dichloride or diaminedichloride or diaminodichloride or diamminedichloride)).tw,kw. (31)

62 cisplatin.rn. (169431)

63 docetaxel.tw,kw. (32095)

64 (daxotel or dexotel or docefrez or lit 976 or lit976 or nsc 628503 or nsc628503 or oncodocel or taxoter or taxotere or texot).tw,kw. (5319)

65 docetaxel.rn. (46280)

66 paclitaxel/ (105768)

67 ("abi 007" or abi007 or abraxane or anzatax or asotax or biotax or bms 181339 or bms181339 or bristaxol or britaxol or coroxane or formoxol or genexol or hunxol or ifaxol or infinnium or intaxel or "mbt 0206" or mbt0206 or medixel or mitotax or nsc 125973 or nsc125973 or oncogel or onxol or pacitaxel or pacxel or padexol or parexel or paxceed or paxene or paxus or praxel or taxocris or taxol or taxus or taycovit or yewtaxan).tw,kw. (25296)

68 paclitaxel.rn. (94408)

69 vinorelbine.tw,kw. (8319)

70 (anx 530 or anx530 or eunades or exelbine or kw 2307 or kw2307 or navelbin or navelbine or navirel or vinbine or vinelbine).tw,kw. (1467)

71 vinorelbine.rn. (2588)

72 Bevacizumab/ (51774)

73 (altuzan or avastin or bevacizumab or nsc 704865 or nsc704865).tw,kw. (38470)

74 bevacizumab.rn. (43545)

75 lapatinib.tw,kw. (5770)

76 (gw 2016 or gw2016 or gw 572016 or gw572016 or gw 572016f or gw572016f or tykerb or tyver).tw,kw. (1782)

77 pertuzumab.tw,kw. (1594)

78 (monoclonal antibody 2C4 or omnitarg or perjeta or rhumab 2C4).tw,kw. (513)

79 neratinib.tw,kw. (408)

80 (HKI 272 or HKI272 or way 177820 or way177820).tw,kw. (467)

81 anastrozole.tw,kw. (4015)

82 (arimidex or ici d1033 or icid1033 or trozolet or ZD-1033 or ZD1033).tw,kw. (1935)

83 exemestane.tw,kw. (3002)

84 (aromasil or aromasin or aromasine or FCE 24304 or nikidess or pnu 155971 or pnu155971).tw,kw. (617)

85 fulvestrant.tw,kw. (2709)

86 (faslodex or ICI 182,780 or ICI 182780 or zd 182780 or zd182780 or zd 9238 or zd9238 or zm 182780 or zm182780).tw,kw. (6794)

87 letrozole.tw,kw. (5868)

88 (CGS 20267 or CGS20267 or femar or femara).tw,kw. (1330)

89 exp Tamoxifen/ (70239)

90 (ICI-46,474 or ICI-46474 or ICI-47699 or kessar or nolvadex or novaldex or nsc 180973 or nsc180973 or soltamox or tamoplac or tamoxasta or tamoxifene or tomaxithen or zitazonium).tw,kw. (1611)

91 tamoxifen.rn. (61828)

92 or/20-91 [ADJUVANT/NEOADJUVANT CHEMOTHERAPY/THERAPIES, DRUGS OF INTEREST] (2702685)

93 18 and 92 [HER2 BREAST CANCER - ADJUVANT/NEOADJUVANT CHEMOTHERAPY/THERAPIES, DRUGS OF INTEREST] (38706)

94 exp Animals/ not (exp Animals/ and Humans/) (13839772)

95 93 not 94 [ANIMAL-ONLY REMOVED] (21398)

96 (comment or editorial or interview or news or newspaper article).pt. (1675306)

97 (letter not (letter and randomized controlled trial)).pt. (1767472)

98 95 not (96 or 97) [OPINION PIECES REMOVED] (20328)

99 limit 98 to yr="1990-current" [DATE LIMITS APPLIED] (20317)

100 limit 99 to english language [LIMITED TO ENGLISH LANGUAGE] (18949)

101 limit 100 to systematic reviews [Limit not valid in Embase; records were retained] (9238)

102 meta analysis.pt. (74283)

103 exp meta-analysis as topic/ (50700)

104 (meta-analy* or metanaly* or metaanaly* or met analy* or integrative research or integrative review* or integrative overview* or research integration or research overview* or collaborative review*).tw,kw. (244505)

105 (systematic review* or systematic overview* or evidence-based review* or evidence-based overview* or (evidence adj3 (review* or overview*)) or meta-review* or meta-overview* or meta-synthes* or rapid review* or "review of reviews" or technology assessment* or HTA or HTAs).tw,kw. (284824)

106 exp Technology assessment, biomedical/ (20916)

107 (cochrane or health technology assessment or evidence report).jw. (34688)

108 ((indirect* or mixed or multi-treatment*) adj2 compar*).tw,kw. (8737)

109 ((network* or network-based) adj (MA or MAs)).kw,tw. (11)

110 or/102-109 (523989)

111 100 and 110 (450)

112 101 or 111 [REVIEWS / META-ANALYSES] (9333)

113 (controlled clinical trial or randomized controlled trial).pt. (519865)

114 "Clinical Trials as Topic".sh. (180217)

115 Randomized Controlled Trials as Topic/ (166815)

116 (randomi#ed or randomly or RCT$1 or placebo*).tw,kw. (1750023)

117 ((singl* or doubl* or trebl* or tripl*) adj (mask* or blind* or dumm*)).tw,kw. (318239)

118 trial.ti. (356638)

119 or/113-118 (2235549)

120 100 and 119 [RCTS] (3224)

121 controlled clinical trial.pt. (91818)

122 Controlled Clinical Trial/ or Controlled Clinical Trials as Topic/ (545895)

123 (control* adj2 trial*).tw,kw. (439962)

124 Non-Randomized Controlled Trials as Topic/ (10328)

125 (nonrandom* or non-random* or quasi-random* or quasi-experiment*).tw,kw. (91512)

126 (nRCT or nRCTs or non-RCT$1).tw,kw. (1197)

127 Controlled Before-After Studies/ (168702)

128 (control* adj3 ("before and after" or "before after")).tw,kw. (7254)

129 Interrupted Time Series Analysis/ (152748)

130 (time series adj3 interrupt*).tw,kw. (3573)

131 (pre- adj3 post-).tw,kw. (148690)

132 (pretest adj3 posttest).tw,kw. (7872)

133 Historically Controlled Study/ (187915)

134 (control* adj2 stud$3).tw,kw. (417335)

135 Control Groups/ (258423)

136 (control$ adj2 group$1).tw,kw. (900863)

137 trial.ti. (356638)

138 or/121-137 (2546593)

139 100 and 138 [CCTS/NON-RCTS] (1953)

140 exp Cohort Studies/ (1894487)

141 cohort?.tw,kw. (996615)

142 Retrospective Studies/ (842418)

143 (longitudinal or prospective or retrospective).tw,kw. (2225517)

144 ((followup or follow-up) adj (study or studies)).tw,kw. (91372)

145 Observational study.pt. (26943)

146 (observation$2 adj (study or studies)).tw,kw. (169328)

147 ((population or population-based) adj (study or studies or analys#s)).tw,kw. (38443)

148 ((multidimensional or multi-dimensional) adj (study or studies)).tw,kw. (185)

149 Comparative Study.pt. (1771349)

150 ((comparative or comparison) adj (study or studies)).tw,kw. (179471)

151 exp Case-Control Studies/ (955328)

152 ((case-control* or case-based or case-comparison) adj (study or studies)).tw,kw. (186400)

153 Cross-Sectional Studies/ (285889)

154 ((crosssection* or cross-section*) adj (study or studies or survey?)).tw,kw. (288696)

155 or/140-154 (6162140)

156 100 and 155 [OBSERVATIONAL STUDIES] (4229)

157 112 or 120 or 139 or 156 [ALL STUDY DESIGNS] (13191)

158 157 use ppez (4307) [MEDLINE RECORDS]

159 exp breast cancer/ (603759)

160 ((breast$1 or mamma or mammary) adj3 (adenocarcinoma* or cancer* or carcinoma* or neoplasm* or tumour* or tumor*)).tw,kw. (624530)

161 159 or 160 [BREAST CANCER] (759845)

162 epidermal growth factor receptor 2/ (35048)

163 (ErbB2 or "ErbB 2" or HER2* or "HER 2*" or "c-ErbB2" or "C-ErbB 2").tw,kw. (84076)

164 ((oncoprotein* or onco-protein* or protein* or receptor*) adj1 (neu or neuregulin)).tw,kw. (2743)

165 (CD340 adj1 antigen?).tw,kw. (0)

166 ("p185(c-neu)" or p185erbB).tw,kw. (95)

167 neu proto-oncogene protein*.tw,kw. (4)

168 metastatic lymph node gene 19 protein*.tw,kw. (0)

169 "luminal b".tw,kw. (3500)

170 luminal subtype*.tw,kw. (711)

171 (human adj1 "epidermal growth factor receptor 2").tw,kw. (10097)

172 or/162-171 (97938)

173 161 and 172 [HER2 BREAST CANCER] (68384)

174 adjuvant chemotherapy/ (79476)

175 ((chemotherap* or chemo-therap*) adj5 (adjuvant* or adjuvent* or neoadjuvant* or neo-adjuvant* or neoadjuvent* or neo-adjuvent* or adjunct* or neoadjunct* or neo-adjunct*)).tw,kw. (90712)

176 ((drug therap* or pharmacothera* or pharmaco-therap*) adj5 (adjuvant* or adjuvent* or neoadjuvant* or neo-adjuvant* or neoadjuvent* or neo-adjuvent* or adjunct* or neoadjunct* or neo-adjunct*)).tw,kw. (1733)

177 exp breast cancer/dt (120515)

178 exp antineoplastic agent/ (1572365)

179 ("anti-HER2" or "anti-HER-2").tw,kw. (5057)

180 ((HER2 or "HER 2") adj1 block*).tw,kw. (471)

181 ((HER2 or "HER 2") adj1 antagoni*).tw,kw. (65)

182 exp cancer chemotherapy/ (269320)

183 trastuzumab/ (34858)

184 trastuzumab emtansine/ (1403)

185 (herceptin* or trastuzumab* or TZM).tw,kw. (27821)

186 (ado-trastuzumab* or kadcyla or pro 132365 or pro132365 or "t dm 1" or "t dm1" or tmab mcc dm1).tw,kw. (1200)

187 trastuzumab.rn. (28831)

188 trastuzumab emtansine.rn. (1059)

189 exp cyclophosphamide/ (205396)

190 (alkyroxan or b 518 or b518 or carloxan or ciclofosfamida or ciclolen or cicloxal or clafen or cyclo-cell or cycloblastin or cycloblastine or cyclofosamide or cyclofosfamid or cyclofosfamide or cyclophar or cyclophosphamide or cyclophosphamides or cyclophosphan or cyclophosphane or cyclostin or cycloxan or cyphos or cytophosphan or cytophosphane or cytoxan or endocyclophosphate or endoxan or endoxana or enduxan or genoxal or ledoxan or ledoxina or mitoxan or neosan or neosar or noristan or nsc 26271 or nsc26271 or nsc 2671 or nsc2671 or procytox or procytoxide or semdoxan or sendoxan or syklofosfamid).tw,kw. (97880)

191 cyclophosphamide.rn. (187679)

192 doxorubicin/ (182708)

193 (adriablastin? or adriacin or adriamicin? or adriamycin? or adriblastin? or adrim or adrimedac or adrubicin or amminac or caelix or caelyx or carcinocin or "dox sl" or doxil or DOXO-cell or doxolem or "doxor lyo" or doxorubicin? or doxotec or evacet or farmiblastina or "fi 106" or fi106 or ifadox or lipodox or "mcc 465" or mcc465 or myocet* or nsc 123127 or nsc123127 or onkodox or rastocin? or resmycin or ribodoxo or rubex or rubidox or sarcodoxome or "tlc d 99").tw,kw. (109657)

194 doxorubicin.rn. (171278)

195 epirubicin/ (29051)

196 (4'-Epi-Adriamycin or 4'-Epi-Doxorubicin or 4'-Epi-DXR or 4'-Epiadriamycin or 4'-Epidoxorubicin or binarin or ellence or EPI-cell or epiadriamycin or epidoxo or epidoxorubicin or epidx or epifil or epilem or epirubicin or farmorrubicina or farmorubicin? or IMI-28 or NSC-256942 or NSC256942 or pharmorubicin? or pidorubicin).tw,kw. (13010)

197 epirubicin.rn. (27584)

198 exp fluorouracil/ (145927)

199 (5-FU or 5FU or 5-fluorouracil or 5fluorouracil or 5-fluoruracil or 5fluoruracil or accusite or actino-hermal or adrucil or carac or effluderm or efudex or efudix or efurix or f6627 or fivoflu or fluoro-uracile or fluoroblastin or fluoroplex or fluorouracil or fluoruracil or fluouracil or fluracedyl or flurodex or fluracil or fluracilium or fluril or fluroblastin or flurouracil or fluoxan or haemato-fu or ifacil or neofluor or nsc 18913 or nsc18913 or nsc 19893 or nsc19893 or oncofu or onkofluor or ribofluor or uflahex or utoral or verrumal).tw,kw. (85471)

200 fluorouracil.rn. (132472)

201 capecitabine/ (25508)

202 (apecitab or ecansya or capecitabine or xeloda).tw,kw. (15308)

203 capecitabine.rn. (21653)

204 methotrexate/ (162708)

205 (amethopterine or abitrexate or amethopterin or amethopterine or ametopterine or antifolan or biotrexate or canceren or cl 14377 or cl4377 or emtexate or emthexat or emthexate or emtrexate or enthexate or farmitrexat or farmitrexate or farmotrex or folex or ifamet or imeth or intradose MTX or lantarel or ledertrexate or maxtrex or metex or methoblastin or methohexate or methotrate or methotrexat or methotrexate or methotrexato or methoxtrexate or methrotrexate or methylaminopterin or methylaminopterine or meticil or metoject or metothrexate or metotrexat or metotrexate or metotrexin or metrex or mexate or mpi 5004 or mpi5004 or neotrexate or novatrex or nsc 740 or nsc740 or otrexup or rasuvo or reumatrex or rheumatrex or texate or texate-t or texorate or trexall or xaken or zexate).tw,kw. (82437)

206 methotrexate.rn. (146922)

207 gemcitabine/ (43132)

208 (gemcitabine or gemcite or gemzar or ly 188011 or ly188011).tw,kw. (33793)

209 gemcitabine.rn. (44783)

210 carboplatin/ (64243)

211 (blastocarb or boplatex or carboplat or carboplatin or carboplatino or carbosin or carbotec or carplan or CBDCA or cycloplatin or erbakar or ercar or ifacap or kemocarb or nsc 241240 or nsc241240 or oncocarbin or paraplatin or paraplatine).tw,kw. (33222)

212 carboplatin.rn. (58198)

213 cisplatin/ (185363)

214 (abiplatin or biocisplatinum or biocysplatinum or blastolem or briplatin or cis ddp or cis diamine dichloroplatinum or cis diaminechloroplatinum or cis diaminedichloroplatinum or cis diammine dichloroplatinum or cis diamminedichloroplatinum or cis dichloridiammineplatinum or cis dichloroadiamine platinum or cis dichlorodiamine platinum or cis dichlorodiamineplatinum or cis dichlorodiammine platinum or cis dichlorodiammineplatinum or cis platinous diamino dichloride or cis platinum or cisplatin or cisplatine or cisplatino or cisplatinum or cisplatyl or citoplatino or cytoplatin or cytosplat).tw,kw. (124715)

215 (diamine dichloroplatinum or diaminodichloroplatinum or diamminedichloroplatinum or dichlorodiamine platinum or dichlorodiammineplatinum or docistin or elvecis or kemoplat or lederplatin or lipoplatin or mpi 5010 or mpi5010 or neoplatin or niyaplat or nk 801 or nk801 or noveldexis or nsc 119875 or nsc119875 or platamine or platiblastin or platidiam or platimine or platinex or platinil or platinol or platinoxan or platiran or platistil or platistin or platosin or randa or romcis or sicatem or "spi 077" or tecnoplatin).tw,kw. (6592)

216 (platinum* adj1 (diaminodichloride or diamino dichloride or diamine dichloride or diaminedichloride or diaminodichloride or diamminedichloride)).tw,kw. (31)

217 cisplatin.rn. (169431)

218 docetaxel/ (45177)

219 (daxotel or dexotel or docefrez or docetaxel or lit 976 or lit976 or nsc 628503 or nsc628503 or oncodocel or taxoter or taxotere or texot).tw,kw. (34659)

220 docetaxel.rn. (46280)

221 paclitaxel/ (105768)

222 ("abi 007" or abi007 or abraxane or anzatax or asotax or biotax or bms 181339 or bms181339 or bristaxol or britaxol or coroxane or formoxol or genexol or hunxol or ifaxol or infinnium or intaxel or "mbt 0206" or mbt0206 or medixel or mitotax or nsc 125973 or nsc125973 or oncogel or onxol or pacitaxel or pacxel or padexol or parexel or paxceed or paxene or paxus or praxel or taxocris or taxol or taxus or taycovit or yewtaxan).tw,kw. (25296)

223 paclitaxel.rn. (94408)

224 navelbine/ (15358)

225 (anx 530 or anx530 or eunades or exelbine or kw 2307 or kw2307 or navelbin or navelbine or navirel or vinbine or vinelbine or vinorelbine).tw,kw. (9106)

226 vinorelbine.rn. (2588)

227 bevacizumab/ (51774)

228 (altuzan or avastin or bevacizumab or nsc 704865 or nsc704865).tw,kw. (38470)

229 bevacizumab.rn. (43545)

230 lapatinib/ (9527)

231 (lapatinib or gw 2016 or gw2016 or gw 572016 or gw572016 or gw 572016f or gw572016f or tykerb or tyver).tw,kw. (7022)

232 lapatinib.rn. (9077)

233 pertuzumab/ (2858)

234 (monoclonal antibody 2C4 or omnitarg or perjeta or pertuzumab or rhumab 2C4).tw,kw. (1937)

235 pertuzumab.rn. (312)

236 neratinib/ (850)

237 (HKI 272 or HKI272 or neratinib or way 177820 or way177820).tw,kw. (793)

238 neratinib.rn. (719)

239 anastrozole/ (8072)

240 (anastrozole or arimidex or ici d1033 or icid1033 or trozolet or ZD-1033 or ZD1033).tw,kw. (5187)

241 anastrozole.rn. (8477)

242 exemestane/ (5039)

243 (aromasil or aromasin or aromasine or exemestane or FCE 24304 or nikidess or pnu 155971 or pnu155971).tw,kw. (3406)

244 exemestane.rn. (5007)

245 fulvestrant/ (6707)

246 (faslodex or fulvestrant or ICI 182,780 or ICI 182780 or zd 182780 or zd182780 or zd 9238 or zd9238 or zm 182780 or zm182780).tw,kw. (8906)

247 fulvestrant.rn. (7881)

248 letrozole/ (9068)

249 (CGS 20267 or CGS20267 or femar or femara or letrozole).tw,kw. (6627)

250 letrozole.rn. (9393)

251 exp Tamoxifen/ (70239)

252 (ICI-46,474 or ICI-46474 or ICI-47699 or kessar or nolvadex or novaldex or nsc 180973 or nsc180973 or soltamox or tamoplac or tamoxasta or tamoxifene or tomaxithen or zitazonium).tw,kw. (1611)

253 tamoxifen.rn. (61828)

254 or/174-253 [ADJUVANT/NEOADJUVANT CHEMOTHERAPY/THERAPIES, DRUGS OF INTEREST] (2029198)

255 173 and 254 [HER2 BREAST CANCER - ADJUVANT/NEOADJUVANT CHEMOTHERAPY/THERAPIES, DRUGS OF INTEREST] (37541)

256 exp animal experimentation/ or exp models animal/ or exp animal experiment/ or nonhuman/ or exp vertebrate/ (39205011)

257 exp human/ or exp human experimentation/ or exp human experiment/ (31545349)

258 256 not 257 (7660817)

259 255 not 258 [ANIMAL-ONLY REMOVED] (36735)

260 editorial.pt. (909450)

261 letter.pt. not (letter.pt. and randomized controlled trial/) (1762470)

262 259 not (260 or 261) [OPINION PIECES REMOVED] (35567)

263 limit 262 to yr="1990-current" [DATE LIMITS APPLIED] (35528)

264 limit 263 to english language [LIMITED TO ENGLISH LANGUAGE] (33570)

265 meta-analysis/ (223804)

266 "systematic review"/ (141277)

267 "meta analysis (topic)"/ (35236)

268 (meta-analy* or metanaly* or metaanaly* or met analy* or integrative research or integrative review* or integrative overview* or research integration or research overview* or collaborative review*).tw,kw. (244505)

269 (systematic review* or systematic overview* or evidence-based review* or evidence-based overview* or (evidence adj3 (review* or overview*)) or meta-review* or meta-overview* or meta-synthes* or rapid review* or "review of reviews" or technology assessment* or HTA or HTAs).tw,kw. (284824)

270 biomedical technology assessment/ (19807)

271 (cochrane or health technology assessment or evidence report).jw. (34688)

272 ((indirect* or mixed or multi-treatment*) adj2 compar*).tw,kw. (8737)

273 ((network* or network-based) adj (MA or MAs)).kw,tw. (11)

274 or/265-273 (566686)

275 264 and 274 [REVIEWS / META-ANALYSES] (1057)

276 randomized controlled trial/ or controlled clinical trial/ (1116359)

277 exp "clinical trial (topic)"/ (260844)

278 (randomi#ed or randomly or RCT$1 or placebo*).tw,kw. (1750023)

279 ((singl* or doubl* or trebl* or tripl*) adj (mask* or blind* or dumm*)).tw,kw. (318239)

280 trial.ti. (356638)

281 or/276-280 (2419104)

282 264 and 281 [RCTS] (7298)

283 exp controlled clinical trial/ (1116483)

284 exp "controlled clinical trial (topic)"/ (127869)

285 (control* adj2 trial*).tw,kw. (439962)

286 (nonrandom* or non-random* or quasi-random* or quasi-experiment*).tw,kw. (91512)

287 (nRCT or nRCTs or non-RCT$1).tw,kw. (1197)

288 (control* adj3 ("before and after" or "before after")).tw,kw. (7254)

289 time series analysis/ (23327)

290 (time series adj3 interrupt*).tw,kw. (3573)

291 pretest posttest control group design/ (329)

292 (pre- adj3 post-).tw,kw. (148690)

293 (pretest adj3 posttest).tw,kw. (7872)

294 controlled study/ (5179828)

295 (control* adj2 stud$3).tw,kw. (417335)

296 Control Groups/ (258423)

297 control group/ (258423)

298 trial.ti. (356638)

299 or/283-298 (6664778)

300 264 and 299 [CCTS/NON-RCTS] (9289)

301 cohort analysis/ (499956)

302 cohort?.tw,kw. (996615)

303 retrospective study/ (1102394)

304 longitudinal study/ (206733)

305 prospective study/ (808493)

306 (longitudinal or prospective or retrospective).tw,kw. (2225517)

307 follow up/ (1170232)

308 ((followup or follow-up) adj (study or studies)).tw,kw. (91372)

309 observational study/ (147400)

310 (observation$2 adj (study or studies)).tw,kw. (169328)

311 population research/ (87628)

312 ((population or population-based) adj (study or studies or analys#s)).tw,kw. (38443)

313 ((multidimensional or multi-dimensional) adj (study or studies)).tw,kw. (185)

314 exp comparative study/ (2690197)

315 ((comparative or comparison) adj (study or studies)).tw,kw. (179471)

316 exp case control study/ (955328)

317 ((case-control* or case-based or case-comparison) adj (study or studies)).tw,kw. (186400)

318 cross sectional study/ (428966)

319 ((crosssection* or cross-section*) adj (study or studies or survey?)).tw,kw. (288696)

320 or/301-319 (7532633)

321 264 and 320 [OBSERVATIONAL STUDIES] (10720)

322 275 or 282 or 300 or 321 [ALL STUDY DESIGNS] (18628)

323 322 use emed (15245) [EMBASE RECORDS]

324 158 or 323 (19552) [BOTH DATABASES]

325 112 use ppez (449)

326 275 use emed (793)

327 325 or 326 (1242)

328 remove duplicates from 327 (986) [UNIQUE REVIEWS]

329 120 use ppez (1979)

330 282 use emed (5921)

331 329 or 330 (7900)

332 331 not 327 (7233)

333 limit 332 to yr="2010-CURRENT" (5618)

334 remove duplicates from 333 (4580)

335 332 not 333 (1615)

336 remove duplicates from 335 (1203)

337 334 or 336 [UNIQUE RCTS] (5783)

338 139 use ppez (871)

339 300 use emed (8229)

340 338 or 339 (9100)

341 340 not (331 or 327) (4857)

342 remove duplicates from 341 [UNIQUE NON-RCTS/CCTS] (4554)

343 156 use ppez (2751)

344 321 use emed (8356)

345 343 or 344 (11107)

346 345 not (327 or 331 or 340) (6220)

347 limit 346 to yr="2010-current" (4884)

348 remove duplicates from 347 (4260)

349 346 not 347 (1336)

350 remove duplicates from 349 (1146)

351 348 or 350 [UNIQUE OBSERVATIONAL STUDIES] (5406)

352 328 or 337 or 342 or 351 (16729)

353 328 use ppez (427) [UNIQUE MEDLINE REVIEWS]

354 328 use emed (559) [UNIQUE EMBASE REVIEWS]

355 337 use ppez (1673) [UNIQUE MEDLINE RCTS]

356 337 use emed (4110) [UNIQUE EMBASE RCTS]

357 342 use ppez (175) [UNIQUE MEDLINE NON-RCTS]

358 342 use emed (4379) [UNIQUE EMBASE NON-RCTS]

359 351 use ppez (1813) [UNIQUE MEDLINE OBSERVATIONAL STUDIES]

360 351 use emed (3593) [UNIQUE EMBASE OBSERVATIONAL STUDIES]

361 352 use ppez (4088) [UNIQUE MEDLINE RECORDS – ALL STUDY DESIGNS]

362 352 use emed (12641) [UNIQUE EMBASE RECORDS – ALL STUDY DESIGNS]

***************************

Cochrane Library

Search Name: HER2 Breast Cancer - Herceptin - Final

Date Run: 19/10/16 23:49:07.686

Description: Cornerstone Research - 2016 Sep 9

ID Search Hits

#1 [mh "Breast Neoplasms"] 9857

#2 ((breast* or mamma or mammary) near/3 (adenocarcinoma* or cancer* or carcinoma* or neoplasm* or tumour* or tumor*)):ti,ab,kw 22260

#3 #1 or #2 22260

#4 [mh "Receptor, ErbB-2"] 596

#5 (ErbB2 or "ErbB 2" or HER2* or (HER next 2*) or "c-ErbB2" or "C-ErbB 2"):ti,ab,kw 4206

#6 ((oncoprotein* or onco-protein* or protein* or receptor*) near/1 (neu or neuregulin)):ti,ab,kw 23

#7 (CD340 near/1 antigen*):ti,ab,kw 0

#8 ("p185(c-neu)" or p185erbB):ti,ab,kw 1

#9 ("neu proto-oncogene" next protein*):ti,ab,kw 0

#10 ("metastatic lymph node gene 19" next protein*):ti,ab,kw 0

#11 "luminal b":ti,ab,kw 69

#12 (luminal next subtype*):ti,ab,kw 17

#13 (human near/1 "epidermal growth factor receptor 2"):ti,ab,kw 298

#14 [mh "Receptor, Epidermal Growth Factor"] Publication Year from 2006 to 2007 42

#15 {or #4-#14} 4289

#16 #3 and #15 2064

#17 [mh ^"Combined Modality Therapy"] Publication Year from 1984 to 1991 1959

#18 [mh "Chemotherapy, Adjuvant"] 3724

#19 ((chemotherap* or chemo-therap*) near/5 (adjuvant* or adjuvent* or neoadjuvant* or neo-adjuvant* or neoadjuvent* or neo-adjuvent* or adjunct* or neoadjunct* or neo-adjunct*)):ti,ab,kw 8121

#20 (((drug next therap*) or pharmacothera* or pharmaco-therap*) near/5 (adjuvant* or adjuvent* or neoadjuvant* or neo-adjuvant* or neoadjuvent* or neo-adjuvent* or adjunct* or neoadjunct* or neo-adjunct*)):ti,ab,kw 667

#21 [mh "Breast Neoplasms"/DT] 4385

#22 [mh "Antineoplastic Agents"] 11658

#23 ("anti-HER2" or "anti-HER 2"):ti,ab,kw 111

#24 ((HER2 or "HER 2") near/1 block*):ti,ab,kw 20

#25 ((HER2 or "HER 2") near/1 antagoni*):ti,ab,kw 0

#26 [mh "Receptor, ErbB-2"/AI] 56

#27 [mh "Antineoplastic Combined Chemotherapy Protocols"] 12366

#28 [mh Trastuzumab] 212

#29 (herceptin* or trastuzumab* or TZM):ti,ab,kw 1092

#30 ((ado next trastuzumab*) or kadcyla or "pro 132365" or pro132365 or "t dm 1" or "t dm1" or "tmab mcc dm1"):ti,ab,kw 35

#31 [mh Cyclophosphamide] 4096

#32 (alkyroxan or "b 518" or b518 or carloxan or ciclofosfamida or ciclolen or cicloxal or clafen or "cyclo-cell" or cycloblastin or cycloblastine or cyclofosamide or cyclofosfamid or cyclofosfamide or cyclophar or cyclophosphamide or cyclophosphamides or cyclophosphan or cyclophosphane or cyclostin or cycloxan or cyphos or cytophosphan or cytophosphane or cytoxan or endocyclophosphate or endoxan or endoxana or enduxan or genoxal or ledoxan or ledoxina or mitoxan or neosan or neosar or noristan or "nsc 26271" or nsc26271 or "nsc 2671" or nsc2671 or procytox or procytoxide or semdoxan or sendoxan or syklofosfamid):ti,ab,kw 8235

#33 [mh Doxorubicin] 3585

#34 (adriablastin* or adriacin or adriamicin* or adriamycin* or adriblastin* or adrim or adrimedac or adrubicin or amminac or caelix or caelyx or carcinocin or "dox sl" or doxil or "DOXO-cell" or doxolem or "doxor lyo" or doxorubicin* or doxotec or evacet or farmiblastina or "fi 106" or fi106 or ifadox or lipodox or "mcc 465" or mcc465 or myocet* or "nsc 123127" or nsc123127 or onkodox or rastocin* or resmycin or ribodoxo or rubex or rubidox or sarcodoxome or "tlc d 99"):ti,ab,kw 6112

#35 [mh Epirubicin] 838

#36 ("4'-Epi-Adriamycin" or "4'-Epi-Doxorubicin" or "4'-Epi-DXR" or "4'-Epiadriamycin" or "4'-Epidoxorubicin" or binarin or ellence or "EPI-cell" or epiadriamycin or epidoxo or epidoxorubicin or epidx or epifil or epilem or epirubicin or farmorrubicina or farmorubicin* or "IMI-28" or "NSC-256942" or NSC256942 or pharmorubicin* or pidorubicin):ti,ab,kw 2231

#37 [mh Fluorouracil] 4282

#38 ("5-FU" or 5FU or "5-fluorouracil" or 5fluorouracil or "5-fluoruracil" or 5fluoruracil or accusite or actino-hermal or adrucil or carac or effluderm or efudex or efudix or efurix or f6627 or fivoflu or "fluoro-uracile" or fluoroblastin or fluoroplex or fluorouracil or fluoruracil or fluouracil or fluracedyl or flurodex or fluracil or fluracilium or fluril or fluroblastin or flurouracil or fluoxan or "haemato-fu" or ifacil or neofluor or "nsc 18913" or nsc18913 or "nsc 19893" or nsc19893 or oncofu or onkofluor or ribofluor or uflahex or utoral or verrumal):ti,ab,kw 8513

#39 [mh Capecitabine] 379

#40 (apecitab or ecansya or capecitabine or xeloda):ti,ab,kw 1525

#41 [mh Methotrexate] 3093

#42 (amethopterine or abitrexate or amethopterin or amethopterine or ametopterine or antifolan or biotrexate or canceren or "cl 14377" or cl4377 or emtexate or emthexat or emthexate or emtrexate or enthexate or farmitrexat or farmitrexate or farmotrex or folex or ifamet or imeth or "intradose MTX" or lantarel or ledertrexate or maxtrex or metex or methoblastin or methohexate or methotrate or methotrexat or methotrexate or methotrexato or methoxtrexate or methrotrexate or methylaminopterin or methylaminopterine or meticil or metoject or metothrexate or metotrexat or metotrexate or metotrexin or metrex or mexate or "mpi 5004" or mpi5004 or neotrexate or novatrex or "nsc 740" or nsc740 or otrexup or rasuvo or reumatrex or rheumatrex or texate or texate-t or texorate or trexall or xaken or zexate):ti,ab,kw 6870

#43 gemcitabine:ti,ab,kw 2399

#44 (gemcite or gemzar or "ly 188011" or ly188011):ti,ab,kw 29

#45 [mh Carboplatin] 1134

#46 (blastocarb or boplatex or carboplat or carboplatin or carboplatino or carbosin or carbotec or carplan or CBDCA or cycloplatin or erbakar or ercar or ifacap or kemocarb or "nsc 241240" or nsc241240 or oncocarbin or paraplatin or paraplatine):ti,ab,kw 3262

#47 [mh Cisplatin] 3586

#48 (abiplatin or biocisplatinum or biocysplatinum or blastolem or briplatin or "cis ddp" or cis diamine dichloroplatinum or cis diaminechloroplatinum or cis diaminedichloroplatinum or cis diammine dichloroplatinum or cis diamminedichloroplatinum or cis dichloridiammineplatinum or cis dichloroadiamine platinum or cis dichlorodiamine platinum or cis dichlorodiamineplatinum or cis dichlorodiammine platinum or cis dichlorodiammineplatinum or cis platinous diamino dichloride or cis platinum or cisplatin or cisplatine or cisplatino or cisplatinum or cisplatyl or citoplatino or cytoplatin or cytosplat):ti,ab,kw 8741

#49 (diamine dichloroplatinum or diaminodichloroplatinum or diamminedichloroplatinum or dichlorodiamine platinum or dichlorodiammineplatinum or docistin or elvecis or kemoplat or lederplatin or lipoplatin or "mpi 5010" or mpi5010 or neoplatin or niyaplat or "nk 801" or nk801 or noveldexis or "nsc 119875" or nsc119875 or platamine or platiblastin or platidiam or platimine or platinex or platinil or platinol or platinoxan or platiran or platistil or platistin or platosin or randa or romcis or sicatem or "spi 077" or tecnoplatin):ti,ab,kw 104

#50 (platinum* near/1 (diaminodichloride or "diamino dichloride" or "diamine dichloride" or diaminedichloride or diaminodichloride or diamminedichloride)):ti,ab,kw 2

#51 docetaxel:ti,ab,kw 3142

#52 (daxotel or dexotel or docefrez or "lit 976" or lit976 or "nsc 628503" or nsc628503 or oncodocel or taxoter or taxotere or texot):ti,ab,kw 189

#53 [mh Paclitaxel] 1747

#54 ("abi 007" or abi007 or abraxane or anzatax or asotax or biotax or "bms 181339" or bms181339 or bristaxol or britaxol or coroxane or formoxol or genexol or hunxol or ifaxol or infinnium or intaxel or "mbt 0206" or mbt0206 or medixel or mitotax or "nsc 125973" or nsc125973 or oncogel or onxol or pacitaxel or pacxel or padexol or parexel or paxceed or paxene or paxus or praxel or taxocris or taxol or taxus or taycovit or yewtaxan):ti,ab,kw 638

#55 vinorelbine:ti,ab,kw 811

#56 ("anx 530" or anx530 or eunades or exelbine or "kw 2307" or kw2307 or navelbin or navelbine or navirel or vinbine or vinelbine):ti,ab,kw 478

#57 [mh Bevacizumab] 624

#58 (altuzan or avastin or bevacizumab or "nsc 704865" or "nsc704865"):ti,ab,kw 2173

#59 lapatinib:ti,ab,kw 368

#60 ("gw 2016" or gw2016 or "gw 572016" or gw572016 or "gw 572016f" or gw572016f or tykerb or tyver):ti,ab,kw 25

#61 pertuzumab:ti,ab,kw 114

#62 ("monoclonal antibody 2C4" or omnitarg or perjeta or "rhumab 2C4"):ti,ab,kw 6

#63 neratinib:ti,ab,kw 19

#64 ("HKI 272" or HKI272 or "way 177820" or way177820):ti,ab,kw 3

#65 anastrozole:ti,ab,kw 687

#66 (arimidex or "ici d1033" or icid1033 or trozolet or "ZD-1033" or ZD1033):ti,ab,kw 171

#67 exemestane:ti,ab,kw 477

#68 (aromasil or aromasin or aromasine or "FCE 24304" or nikidess or "pnu 155971" or pnu155971):ti,ab,kw 22

#69 fulvestrant:ti,ab,kw 190

#70 (faslodex or "ICI 182,780" or "ICI 182780" or "zd 182780" or zd182780 or "zd 9238" or zd9238 or "zm 182780" or zm182780):ti,ab,kw 63

#71 letrozole:ti,ab,kw 867

#72 ("CGS 20267" or CGS20267 or femar or femara):ti,ab,kw 35

#73 [mh Tamoxifen] 1995

#74 ("ICI-46,474" or "ICI-46474" or "ICI-47699" or kessar or nolvadex or novaldex or "nsc 180973" or nsc180973 or soltamox or tamoplac or tamoxasta or tamoxifene or tomaxithen or zitazonium):ti,ab,kw 101

#75 {or #17-#74} 51148

#76 #16 and #75 Publication Year from 1990 to 2016 1709

DSR - 7

DARE - 40

CENTRAL – 1584

Methods – 5 [*did not download*]

HTA - 41

NHS EED – 32 [*did not download*]

Eligibility Criteria

Table 1 Summary of eligibility criteria for the systematic review and cumulative network meta-analysis

| **Item** | **Description** |
| --- | --- |
| **Population** | Adult patients (≥18 years) with human epidermal growth factor receptor 2-positive (HER2+) early breast cancer (EBC) (stages 0 to IIIC), locally advanced, or inflammatory breast cancer, receiving neoadjuvant and/or adjuvant therapies. |
| **Intervention/**  **Index Node** | Trastuzumab (Herceptin®; H) administered intravenously (IV) for the treatment of HER2+ EBC. All trastuzumab doses, formulations, treatment schedules, and durations were eligible. Trastuzumab may be combined with any other drug regimen. |
| **Comparators** | All doses, formulations, and treatment durations of the following drug therapies were eligible:   - **Chemotherapy agents**: capecitabine, carboplatin, cisplatin, cyclophosphamide, docetaxel, doxorubicin, epirubicin, 5-fluorouracil, gemcitabine, methotrexate, paclitaxel, paclitaxel-liposomal, and vinorelbine - **Hormonal therapies**: anastrozole, exemestane, fulvestrant, letrozole, and tamoxifen - **Targeted therapies**: bevacizumab, lapatinib, neratinib, pertuzumab, trastuzumab-subcutaneous (SC), and trastuzumab emtansine - Any combination of the above therapies |
| **Outcomes** | Overall survival (OS) was the primary outcome of interest. We also included studies that evaluated pathologic complete response (pCR) or a measure of x-free survival (xFS), where x stands for measures such as disease (DFS), invasive disease (iDFS), event (EFS), and recurrence (RFS). |
| **Study design** | - Randomized, controlled, prospective clinical trials (Phase II and III) - Non-randomized studies (e.g., case-control, cross-sectional, longitudinal, and cohort studies) - Conference abstracts, posters, and presentations - Meta-analyses and systematic reviews were also included as secondary sources to ensure that no primary studies were missed |
| **Language** | English only |
| **Publication Date** | January 1, 1990, to January 19, 2017 |

Risk of Bias Assessment

A risk of bias assessment of the included RCTs was completed in duplicate by two experienced reviewers (Table 2). Overall, there was a low risk of bias across most studies for the categories “random sequence generation”, “allocation concealment”, “blinding of outcome assessment”, and “selective reporting”. The category “incomplete outcome data” showed a low or unclear risk of bias for most studies, and the category “blinding of participants and personnel” showed a high risk of bias for most studies due to open-label design. An unclear risk of bias was most often due to a lack of information available.

Several RCTs allowed for crossover, but a lack of information prevented us from performing a sensitivity analysis to account for crossover. The cumulative NMA required that consistent values were used for all analyses, but adjusted OS values were not available for all publications from trials that used a crossover design. Therefore, a sensitivity analysis was not feasible based on the lack of information related to crossover and the incorporation of this information into the cumulative NMA. In addition, although some crossover results reduced the apparent benefit of H, our analyses still showed a robust and persistent improvement in OS. The three main trials where crossover had an effect are described below.

In the HERA trial, 52.1% of patients in the observation group crossed over to receive H before a disease-free survival event.^2^ Based on the intention-to-treat analysis, this crossover biased the results against H and reduced the apparent benefit of 1 year of H. Despite this, H showed a persistent improvement in OS.^2^ Although unadjusted and censored hazard ratios are available from the 2007 and 2011 publications,^3,4^ no adjusted OS result is reported in the 2013 publication.^2^

In the ALTTO trial, the lapatinib arm was closed four years into the study and patients free of disease were offered adjuvant H; 52% of patients initially assigned to lapatinib received at least one dose of H before a disease-free survival event.^5^ Again, this biased the results against H, yet the final OS results showed a benefit for H.^5^

In the NOAH trial, 16% of patients initially randomised to receive chemotherapy alone received H after surgery within 8 months of randomisation.^6^ Although an unadjusted and inverse probability of censoring weighted analysis of OS is reported in the 2014 publication,^6^ no adjusted OS result is reported in the 2010 publication.^7^

Table 2 Risk of bias assessment of included randomized controlled trials

| **Trial** | **Random sequence generation** | **Allocation concealment** | **Blinding of participants and personnel** | **Blinding of outcome assessment** | **Incomplete outcome data** | **Selective reporting** |
| --- | --- | --- | --- | --- | --- | --- |
| **RCTs with 100% HER2+ patients** | | | | | | |
| ALTTO (Piccart-Gebhart 2016)^5^ | + | ? | - | + | ? | ? |
| BCIRG 006 (Slamon 2011)^8^ | ? | ? | - | + | + | + |
| FNCLCC-PACS 04 (Spielmann 2009)^9^ | ? | ? | - | + | ? | - |
| HannaH (Jackisch 2016)^10^ | + | + | - | + | ? | + |
| HERA (Goldhirsch 2013)^2^ | + | + | - | + | ? | + |
| HORG (Mavroudis 2015)^11^ | + | + | ? | + | ? | + |
| NCCTG N9831 & NSABP B-31  (Perez 2014)^12^ | + | ? | - | + | ? | + |
| NeoALTTO (de Azambuja 2014)^13^ | + | + | - | + | - | + |
| NOAH (Gianni 2014)^6^ | + | + | - | + | + | + |
| NSABP B-41 (Robidoux 2013)^14^ | ? | + | - | + | ? | ? |
| PHARE (Pivot 2013)^15^ | + | + | - | + | + | + |
| **RCTs with HER2+ Subgroups** | | | | | | |
| BCIRG 001 (Mackey 2013)^16^ | + | + | - | + | ? | + |
| BR9601 & NEAT (Earl 2012)^17^ | + | + | ? | + | + | + |
| E1199 (Sparano 2015)^18^ | ? | ? | ? | + | - | + |
| E2198 (Schneider 2015)^19^ | + | ? | ? | + | + | + |
| FinHer (Joensuu 2009)^20^ | ? | + | - | + | + | + |
| FinXX (Joensuu 2014)^21^ | + | + | - | + | - | + |
| GeparTrio (von Minckwitz 2013)^22^ | ? | ? | - | + | + | + |
| GONO-MIG-1 (Del Mastro 2005)^23^ | ? | ? | ? | + | ? | + |
| TEACH (Goss 2013)^24^ | + | ? | + | + | + | + |
| UNICANCER-PACS01 (Coudert 2012)^25^ | + | ? | ? | + | ? | + |
| Del Mastro 2015^26^ | + | + | - | + | + | + |
| Rocca 2014^27^ | + | - | - | + | ? | + |
| Boccardo 2010^28^ | + | + | - | + | + | + |
| Miles 1999^29^ | ? | ? | ? | + | ? | + |
| Colozza 2005^30^ | + | + | ? | + | ? | + |

+ low risk of bias, - high risk of bias, ? unclear risk of bias, *HER2+* human epidermal growth factor receptor 2-positive, *RCT* randomized controlled trial.

A risk of bias assessment of the three non-randomized studies that were included in the OS NMA was performed using the ROBINS-I tool (Table 3).^31,32^ We used a systematic process to investigate the statistical methods of all potential non-randomized studies, and only high-quality studies that appropriately adjusted for covariates were included. Overall, there was a low risk of bias across the three included studies.

Table 3 Visual representation of the risk of bias assessment of non-randomized studies based on the ROBINS-I tool

| **Trial** | **Confounding** | **Selection bias** | **Classification of interventions** | **Crossover** | **Missing data** | **Assessment of outcomes** | **Selective reporting** |
| --- | --- | --- | --- | --- | --- | --- | --- |
| Bayraktar 2012^33^ | ? | + | + | ? | + | + | + |
| Gonzalez-Angulo 2015^34^ | - | - | + | + | + | + | + |
| Seferina 2015^35^ | + | ? | + | ? | + | + | + |

+ low risk of bias, - high risk of bias, ? unclear risk of bias.

Study and Patient Characteristics

Table 4 Study and patient characteristics from randomized controlled trials with overall survival results

| Study (Primary publication) | Treatments | Node Name in Network | Blinding | Analysis Population | Location | Median Follow-up (months) | N total | Median age (yrs) | Tumour Size >2cm (%) | Node + (%) | ER+ (%) | PgR+ (%) | HR+ (%) | HER2+ (%) |
| --- | --- | --- | --- | --- | --- | --- | --- | --- | --- | --- | --- | --- | --- | --- |
| RCTs with 100% HER2+ early breast cancer patients | | | | | | | | | | | | | | |
| ALTTO (Piccart-Gebhart 2016)^5^ | Anthracycline and/or taxane → trastuzumab IV (52 weeks) | AC-TH_52wk_ | OL | ITT | International | 54 | 2097 | 51 | 49 | 51 | NR | NR | 57 | 100 |
|  | Anthracycline and/or taxane → lapatinib (52 weeks) | AC-TL_52wk_ | OL | ITT | International | 54 | 2100 | 51 | 51 | 52 | NR | NR | 57 | 100 |
|  | Anthracycline and/or taxane → trastuzumab IV (12 weeks) → lapatinib (34 weeks) | AC-TL_34wk_-H_12wk_ | OL | ITT | International | 54 | 2091 | 51 | 50 | 52 | NR | NR | 58 | 100 |
|  | Anthracycline and/or taxane → trastuzumab IV (52 weeks) + lapatinib (52 weeks) | AC-TL_52wk_-H_52wk_ | OL | ITT | International | 54 | 2093 | 51 | 50 | 51 | NR | NR | 57 | 100 |
| BCIRG 006 (Slamon 2011)^8^ | Doxorubicin + cyclophosphamide → docetaxel | AC-T | OL | ITT | International | 65 | 1073 | NR | 59 | 71 | NR | NR | 54 | 100 |
|  | Doxorubicin + cyclophosphamide → docetaxel + trastuzumab IV (52 weeks) | AC-TH_52wk_ | OL | ITT | International | 65 | 1074 | NR | 62 | 71 | NR | NR | 54 | 100 |
|  | Docetaxel + carboplatin + trastuzumab IV (52 weeks) | TCH_52wk_ | OL | ITT | International | 65 | 1075 | NR | 59 | 72 | NR | NR | 54 | 100 |
| FNCLCC-PACS 04 (Spielmann 2009)^9^ | FEC or ED (epirubicin + docetaxel) → trastuzumab IV (52 weeks) | AC-TH_52wk_ | OL | ITT | France, Belgium | 47 | 260 | 48 | 59.1 | 100 | NR | NR | 58 | 100 |
|  | FEC or ED (epirubicin + docetaxel) | AC-T | OL | ITT | France, Belgium | 47 | 268 | 49 | 50.6 | 100 | NR | NR | 61 | 100 |
| HannaH (Jackisch 2016)^10^ | Docetaxel → FEC + trastuzumab IV → trastuzumab IV (52 weeks) | AC-TH_52wk_ | OL | ITT | International | 40.6 | 297 | 50 | NR | 79.1 | 49.8 | NR | NR | 100 |
|  | Docetaxel → FEC + trastuzumab SC → trastuzumab SC (52 weeks) | AC-TH_SC,52wk_ | OL | ITT | International | 40.3 | 294 | 50 | NR | 75.8 | 52.4 | NR | NR | 100 |
| HERA (Goldhirsch 2013)^2^ | Anthracycline or taxane → trastuzumab IV (104 weeks) | AC-TH_104wk_ | OL | ITT | International | 96 | 1700 | NR | 49.5 | 56.5 | NR | NR | 51.4 | 100 |
|  | Anthracycline or taxane → trastuzumab IV (52 weeks) | AC-TH_52wk_ | OL | ITT | International | 96 | 1702 | 49 | 48.4 | 56.4 | NR | NR | 50.9 | 100 |
|  | Anthracycline or taxane | AC-T | OL | ITT | International | 96 | 1697 | 49 | NR | NR | NR | NR | NR | 100 |
| HORG (Mavroudis 2015)^11^ | FEC → docetaxel + trastuzumab IV (52 weeks) | AC-TH_52wk_ | NR | NR | Greece | 47 | 241 | 54 | NR | 74.7 | NR | NR | 64.7 | 100 |
|  | FEC → docetaxel + trastuzumab IV (26 weeks) | AC-TH_26wk_ | NR | NR | Greece | 51 | 240 | 56 | NR | 83.3 | NR | NR | 68.8 | 100 |
| NCCTG N9831 & NSABP B-31 (Perez 2014)^12^ | Doxorubicin + cyclophosphamide → paclitaxel | AC-T | OL | ITT | USA | 99.6 | 2018 | ~50 | 59.2 | 92.6 | NR | NR | 54.8 | 100 |
|  | Doxorubicin + cyclophosphamide → paclitaxel + trastuzumab IV (52 weeks) | AC-TH_52wk_ | OL | ITT | USA | 100.8 | 2028 | ~50 | 61.8 | 93.4 | NR | NR | 54.7 | 100 |
| NeoALTTO (de Azambuja 2014)^13^ | Paclitaxel → FEC → lapatinib (52 weeks) | AC-TL_52wk_ | OL | ITT | International | 45 | 154 | 50 | 100 | >16.2 | NR | NR | 51.9 | 100 |
|  | Paclitaxel → FEC → trastuzumab IV (52 weeks) | AC-TH_52wk_ | OL | ITT | International | 45 | 149 | 49 | 100 | >15.4 | NR | NR | 50.3 | 100 |
|  | Paclitaxel + → FEC → lapatinib (52 weeks) + trastuzumab IV (52 weeks) | AC-TL_52wk_-H_52wk_ | OL | ITT | International | 45 | 152 | 50 | 100 | >15.8 | NR | NR | 50.7 | 100 |
| NOAH (Gianni 2014)^6^ | Paclitaxel + doxorubicin → paclitaxel → CMF | AC-T | OL | ITT | Italy | 64.8 | 118 | NR | NR | 84 | NR | NR | 36 | 100 |
|  | [Paclitaxel + doxorubicin → paclitaxel → CMF → trastuzumab IV (52 weeks) | AC-TH_52wk_ | OL | ITT | Italy | 64.8 | 117 | NR | NR | 86 | NR | NR | 36 | 100 |
| NSABP B-41 (Robidoux 2013†)^14^ | Doxorubicin + cyclophosphamide → paclitaxel → trastuzumab IV (52 weeks) | AC-TH_52wk_ | OL | ITT | USA, Canada | 22.8 | 181 | NR | 100 | 51 | NR | NR | 67 | 100 |
|  | Doxorubicin + cyclophosphamide → paclitaxel + lapatinib (12 weeks) → trastuzumab IV (34 weeks) | AC → T-L_12wk_-H_34wk_ | OL | ITT | USA, Canada | 22.8 | 174 | NR | 100 | 52 | NR | NR | 58 | 100 |
|  | Doxorubicin + cyclophosphamide → paclitaxel + lapatinib (12 weeks) + trastuzumab IV → trastuzumab IV (52 weeks) | AC → T-L_12wk_-H_52wk_ | OL | ITT | USA, Canada | 22.8 | 174 | NR | 100 | 49 | NR | NR | 62 | 100 |
| PHARE (Pivot 2013)^15^ | Anthracycline + taxane + trastuzumab IV→ trastuzumab IV (52 weeks) | AC-TH_52wk_ | OL | ITT | France | 42.5 | 1690 | 54 | 45.3 | 44.6 | 57.6 | 42.4 | 60.4 | 100 |
|  | Anthracycline + taxane + trastuzumab IV → trastuzumab IV (26 weeks) | AC-TH_26wk_ | OL | ITT | France | 42.5 | 1690 | 55 | 47.6 | 45.3 | 58.8 | 41.6 | 61.5 | 100 |
| RCTs with subgroup data of HER2+ early breast cancer patients | | | | | | | | | | | | | | |
| BCIRG 001 (Mackey 2013)^16^ | Docetaxel + doxorubicin + cyclophosphamide | AC-T | OL | ITT | International | 124 | 745 | 49 | 61 | 100 | NR | NR | 76.1 | 21 |
|  | Fluorouracil + doxorubicin + cyclophosphamide | AC | OL | ITT | International | 123 | 746 | 49 | 57 | 100 | NR | NR | 75.7 | 22 |
| Boccardo 2010^28^ | Epirubicin → cyclophosphamide + methotrexate + fluorouracil | AC | OL | As-Treated | Italy | 102 | 122 | 53.0 | 47.5 | 100 | NR | NR | 79.5 | 31.1 |
|  | Paclitaxel → epirubicin + vinorelbine | T → AV | OL | As-Treated | Italy | 102 | 122 | 54.5 | 64.8 | 100 | NR | NR | 79.5 | 28.7 |
| BR9601 & NEAT (Earl 2012)^17^ | Epirubicin → CMF | AC | NR | ITT | Scotland | 88.8 | 1189 | NR | 56 | 72 | 59 | 56 | NR | 21 |
|  | Cyclophosphamide + methotrexate + fluorouracil | CMF | NR | ITT | Scotland | 88.8 | 1202 | NR | 56 | 72 | 59 | 55 | NR | 20 |
| Colozza 2005^30^ | Cyclophosphamide + methotrexate + fluorouracil | CMF | NR | NR | Italy | 96 | 133 | NR | 51 | 80 | 56 | 63 | NR | 69 |
|  | Epirubicin | E | NR | NR | Italy | 96 | 133 | NR | 48 | 78 | 55 | 63 | NR | 77 |
| Del Mastro 2015^26^ | Epirubicin + cyclophosphamide → paclitaxel (q3w) | AC-T | OL | ITT | Italy | 84 | 545 | 51 | 48 | 100 | NR | NR | 77 | 23 |
|  | FEC → paclitaxel (q3w) | AC-T | OL | ITT | Italy | 84 | 544 | 53 | 52 | 100 | NR | NR | 81 | 24 |
|  | Epirubicin + cyclophosphamide → paclitaxel (q2w) | Dose dense | OL | ITT | Italy | 84 | 502 | 53 | 48 | 100 | NR | NR | 81 | 21 |
|  | FEC → paclitaxel (q2w) | Dose dense | OL | ITT | Italy | 84 | 500 | 51 | 49 | 100 | NR | NR | 80 | 24 |
| E1199 (Sparano 2015‡)^18^ | Doxorubicin + cyclophosphamide → paclitaxel (weekly) | Dose dense | NR | ITT | USA | 145.2 | 1232 | 51 | 63.8 | 87.8 | NR | NR | 70.2 | 19.2 |
|  | Doxorubicin + cyclophosphamide → paclitaxel (q3w) | AC-T | NR | ITT | USA | 145.2 | 1253 | 51 | 60.7 | 87.8 | NR | NR | 71.0 | 20.6 |
| E2198 (Schneider 2015)^19^ | Paclitaxel + trastuzumab IV (10 weeks) → doxorubicin + cyclophosphamide | AC-TH_9-10wk_ | NR | Per-Protocol | NR | 77 | 115 | 49 | NR | 100 | 55 | 52 | 60 | 53 |
|  | Paclitaxel + trastuzumab IV (10 weeks) → doxorubicin + cyclophosphamide + trastuzumab IV (52 weeks total) | AC-TH_52wk_ | NR | Per-Protocol | NR | 77 | 112 | 48 | NR | 100 | 59 | 49 | 63 |  |
| FinHer (Joensuu 2009)^20^ | Docetaxel → FEC | AC-T | OL | ITT | Finland | 62 | 58 | 49.9 | 70 | 78 | 44 | 29 | NR | 100* |
|  | Docetaxel + trastuzumab IV (9 weeks) → FEC | AC-TH_9-10wk_ | OL | ITT | Finland | 62 | 54 | 51.4 | 59 | 90 | 50 | 39 | NR | 100* |
|  | Vinorelbine → FEC | AC-V | OL | ITT | Finland | 62 | 58 | 49.9 | 70 | 78 | 44 | 29 | NR | 100* |
|  | Vinorelbine + trastuzumab IV (9 weeks) → FEC | AC-VH_9wk_ | OL | ITT | Finland | 62 | 62 | 51.4 | 59 | 90 | 50 | 39 | NR | 100* |
| FinXX (Joensuu 2014)^21^ | Capecitabine + docetaxel → cyclophosphamide + epirubicin + capecitabine → trastuzumab IV (52 weeks); or docetaxel → FEC → trastuzumab IV (52 weeks) | AC-TH_52wk_ | OL | ITT | Finland, Sweden | 80.4 | 176 | 52.2 | NR | 84.6 | NR | NR | 60.2 | 100* |
|  | Capecitabine + docetaxel → cyclophosphamide + epirubicin + capecitabine; or docetaxel → FEC | AC-T | OL | ITT | Finland, Sweden | 80.4 | 108 | 50.5 | NR | 81.4 | NR | NR | 51.9 | 100* |
| GeparTrio (von Minckwitz 2013)^22^ | Docetaxel + doxorubicin + cyclophosphamide → vinorelbine + capecitabine (in non-responders) | AC-T → VX | OL | ITT | Germany | 62 | 987 | NR | NR | 54.7 | NR | NR | 65.6 | 29.1 |
|  | Docetaxel + doxorubicin + cyclophosphamide | AC-T | OL | ITT | Germany | 62 | 1025 | NR | NR | 55.3 | NR | NR | 63.2 | 30.5 |
| GONO-MIG-1 (Del Mastro 2005)^23^ | FEC (q3w) | AC | NR | ITT | Italy | 80.4 | 53 | 54 | NR | 61.2 | 27.2 | 19.4 | NR | 100* |
|  | FEC (q2w) | Dose dense | NR | ITT | Italy | 80.4 | 50 | 54 | NR | 61.2 | 27.2 | 19.4 | NR | 100* |
| Miles 1999^29^ | Cyclophosphamide + methotrexate + fluorouracil | CMF | NR | NR | UK | 159.6 | 129 | NR | 78 | 100 | 77.5 | NR | NR | 30 |
|  | No treatment | No Tx | NR | NR | UK | 159.6 | 145 | NR | 78 | 100 | 77.5 | NR | NR | 30 |
| Rocca 2014^27^ | Epirubicin → CMF, or CMF → epirubicin | AC | OL | As-Treated | Italy | 69 | 545 | 53 | NR | 48.3 | 64.0 | 51.4 | NR | 34.1 |
|  | Cyclophosphamide + methotrexate + fluorouracil | CMF | OL | As-Treated | Italy | 69 | 160 | 51 | NR | 47.5 | 56.9 | 46.2 | NR | 30.0 |
| TEACH (Goss 2013)^24^ | Lapatinib (52 weeks) + anthracycline/taxane | AC-TL_52wk_ | DB | ITT | International | 47.4 | 1571 | 51 | NR | 54 | NR | NR | 59 | 78 |
|  | Placebo (52 weeks) + anthracycline/taxane | AC-T | DB | ITT | International | 48.3 | 1576 | 52 | NR | 54 | NR | NR | 59 | 80 |
| UNICANCER-PACS01 (Coudert 2012)^25^ | FEC | AC | NR | ITT | France, Belgium | 92.8 | 996 | NR | 57.8 | 100 | 73 | 64 | 78 | 9.4 |
|  | FEC → docetaxel | AC-T | NR | ITT | France, Belgium | 92.8 | 1003 | NR |  |  |  |  |  |  |
| Non-randomized studies with 100% HER2+ early breast cancer patients | | | | | | | | | | | | | | |
| Bayraktar 2012^33^ | Paclitaxel + trastuzumab → FEC + trastuzumab (52 weeks) | AC-TH_52wk_ | NA | NA | USA | 29 | 235 | 49 | NR | 81.5 | 53.8 | NR | NR | 100 |
|  | Docetaxel + carboplatin + trastuzumab (52 weeks) | TCH_52wk_ | NA | NA | USA | 18 | 65 | 53 | NR | 80.0 | 55.6 | NR | NR | 100 |
| Gonzalez-Angulo 2015^34^ | Adjuvant trastuzumab: paclitaxel + trastuzumab → FEC + trastuzumab (52 weeks) | AC-TH_52wk_ | NA | NA | USA | 45 | 480 | ~50 | NR | 19.4 | NR | NR | 61.1 | 100 |
|  | No adjuvant trastuzumab: paclitaxel + trastuzumab (26 weeks) → FEC | AC-TH_26wk_ | NA | NA | USA | 45 | 109 | ~50 | NR | 15.6 | NR | NR | 53.2 | 100 |
| Seferina 2015^35^ | Anthracycline/taxane-based chemotherapy + trastuzumab (52 weeks) | AC-TH_52wk_ | NA | NA | The Netherlands | 60 | 230 | 51 | >56 | 56.0 | NR | NR | 62 | 100 |
|  | Endocrine therapy and radiotherapy, no chemotherapy | No chemo | NA | NA | The Netherlands | 60 | 246 | 65 | NR | NR | NR | NR | NR | 100 |

Data were extracted from the most recent full-text publications, when available.

*Patient characteristics were available for the HER2+ subgroup population. Therefore, the percentage of HER2+ patients in the HER2+ subgroup is 100%, even though it is a non-randomized subset of the RCT. †Patient characteristics for NSABP B-41 were taken from Robidoux 2013^14^ but OS results were not provided in this publication; OS results were taken from Robidoux 2016.^36^ ‡Patient characteristics for E1199 were taken from Sparano 2015^18^ but OS results for HER2+ patients were not provided in this publication; OS results were taken from Sparano 2008.^37^

*AC* anthracycline (doxorubicin, epirubicin) + cyclophosphamide, *AV* anthracycline + vinorelbine, *CMF* cyclophosphamide + methotrexate + fluorouracil, *DB* double blind, *Dose dense* AC → T, or AC, either weekly or biweekly, *E* epirubicin, *ED* epirubicin + docetaxel, *ER+* estrogen receptor-positive, *FEC* fluorouracil + epirubicin + cyclophosphamide, *H* Herceptin® intravenous (IV), *HER2+* human epidermal growth factor receptor 2-positive, *HR+* hormone receptor-positive, *H_SC_* Herceptin® subcutaneous (SC), *ITT* intention to treat, *IV* intravenous, *L* lapatinib, *NA* not applicable, *No Chemo* no chemotherapy (includes endocrine therapy and radiotherapy), *No Tx* no treatment, *NR* not reported, *OL* open label, *OS* overall survival, *PBO* placebo, *PgR+* progesterone receptor-positive, *q2w* once every 2 weeks, *q3w* once every 3 weeks, *RCT* randomized controlled trial, *T* taxane (docetaxel, paclitaxel), *TCH* docetaxel + carboplatin + Herceptin® IV, *V* vinorelbine, *wk* weeks, *X* capecitabine.

Evidence Network Nodes and Treatments

Table 5 Summary of nodes and treatments included in evidence networks

| **Node Name** | **Node Description** | **Treatments Included** | **Study** | **Comments/Rationale** |
| --- | --- | --- | --- | --- |
| **CMF** | Cyclophosphamide, methotrexate, fluorouracil | Cyclophosphamide, methotrexate, fluorouracil | BR9601 & NEAT (Earl 2012)^17^ |  |
|  |  | Cyclophosphamide, methotrexate, fluorouracil | Rocca 2014^27^ |  |
|  |  | Cyclophosphamide, methotrexate, fluorouracil | Miles 1999^29^ |  |
|  |  | Cyclophosphamide, methotrexate, fluorouracil | Colozza 2005^30^ |  |
| **E** | Epirubicin | Epirubicin monotherapy | Colozza 2005^30^ |  |
| **No Tx** | No treatment | No treatment (no radiotherapy or endocrine therapy) | Miles 1999^29^ |  |
| **T → AV** | Anthracycline + taxane-based chemo + vinorelbine | Paclitaxel → epirubicin + vinorelbine | Boccardo 2010^28^ |  |
| **Dose dense (AC → T, or AC, either weekly or biweekly)** | (Bi)weekly anthracycline + taxane-based chemo | Doxorubicin + cyclophosphamide → paclitaxel/docetaxel (qw) | E1199 (Sparano 2008)^37^ | Docetaxel excluded from analyses since HR only available for paclitaxel. |
|  |  | (Fluorouracil +) epirubicin + cyclophosphamide → paclitaxel (q2w) | Del Mastro 2015^26^ | Four-arm trial in which two arms contained fluorouracil, but results were pooled and compared as the dose dense vs. standard regimens. |
|  |  | Fluorouracil + epirubicin + cyclophosphamide (q2w) | GONO-MIG-1 (Del Mastro 2005)^23^ | This treatment does not include a taxane, but it has been grouped with this node because it is a dose dense treatment. |
| **AC** | Fluorouracil + anthracycline + cyclophosphamide | Epirubicin + CMF | BR9601 & NEAT (Earl 2012)^17^ |  |
|  |  | Epirubicin → CMF, or CMF → Epirubicin | Rocca 2014^27^ |  |
|  |  | Epirubicin → CMF | Boccardo 2010^28^ |  |
|  |  | Fluorouracil + epirubicin + cyclophosphamide (q3w) | GONO-MIG-1 (Del Mastro 2005)^23^ |  |
|  |  | Fluorouracil + doxorubicin + cyclophosphamide | BCIRG 001 (Mackey 2013)^16^ |  |
|  |  | Fluorouracil + epirubicin + cyclophosphamide | UNICANCER-PACS-01 (Coudert 2012)^25^ |  |
| **AC-V** | Anthracycline-based chemo + vinorelbine | Vinorelbine + fluorouracil + epirubicin + cyclophosphamide | FinHer (Joensuu 2009)^20^ |  |
| **AC-T → VX** | Anthracycline + taxane-based chemo → (in non-responders) vinorelbine + capecitabine | Docetaxel + doxorubicin + cyclophosphamide → (in non-responders) vinorelbine + capecitabine | GeparTrio (von Minckwitz 2013)^22^ |  |
| **AC-VH_9wk_** | Anthracycline-based chemo + vinorelbine+ trastuzumab IV (9 wks) | Vinorelbine + trastuzumab (qw for 9 wks) → fluorouracil + epirubicin + cyclophosphamide | FinHer (Joensuu 2009)^20^ |  |
| **AC-TH_104wk_** | Anthracycline + taxane-based chemo + trastuzumab IV (104 wks) | Anthracycline or taxane → trastuzumab IV (2 years) | HERA (Goldhirsch 2013)^2^ | 95% of patients received anthracycline and 25.5% received taxane. Based on this, the HERA trial would be more appropriately displayed as a connection to the AC node; however, the AC node does not exist in all networks. Therefore, the HERA trial was forced to connect to the AC-T vs. AC-TH,52wk main “highway” of the network. |
| **AC-T** | Anthracycline + taxane-based chemo (q3w) | Anthracycline or taxane | HERA (Goldhirsch 2013)^2^ | 95% of patients received anthracycline and 25.5% received taxane. Based on this, the HERA trial would be more appropriately displayed as a connection to the AC node; however, the AC node does not exist in all networks. Therefore, the HERA trial was forced to connect to the AC-T vs. AC-TH,52wk main “highway” of the network. |
|  |  | Docetaxel + doxorubicin + cyclophosphamide | GeparTrio (von Minckwitz 2013)^22^ |  |
|  |  | Doxorubicin + cyclophosphamide → paclitaxel (q3w) | E1199 (Sparano 2008)^37^ |  |
|  |  | (Fluorouracil +) epirubicin + cyclophosphamide → paclitaxel (q3w) | Del Mastro 2015^26^ | Four-arm trial in which two arms contained fluorouracil, but results were pooled and compared as the dose dense vs. standard regimens. |
|  |  | Docetaxel + doxorubicin + cyclophosphamide | BCIRG 001 (Mackey 2013)^16^ |  |
|  |  | Fluorouracil + epirubicin + cyclophosphamide → docetaxel | UNICANCER-PACS-01 (Coudert 2012)^25^ |  |
|  |  | Docetaxel → fluorouracil + epirubicin + cyclophosphamide | FinHer (Joensuu 2009)^20^ |  |
|  |  | Doxorubicin + cyclophosphamide → docetaxel | BCIRG 006 (Slamon 2015)^38^ |  |
|  |  | Doxorubicin + cyclophosphamide → paclitaxel | NCCTG N9831 & NSABP B-31 (Perez 2014)^12^ |  |
|  |  | FEC or epirubicin + docetaxel | FNCLCC-PACS 04 (Spielmann 2009)^9^ | ~50% of patients received FEC and ~50% received epirubicin + docetaxel. |
|  |  | Paclitaxel + doxorubicin → cyclophosphamide + methotrexate + fluorouracil | NOAH (Gianni 2014)^6^ |  |
|  |  | Docetaxel → cyclophosphamide + epirubicin + fluorouracil | FinXX (Joensuu 2014)^21^ |  |
|  | Anthracycline + taxane-based chemo + placebo | Placebo (1 year) | TEACH (Goss 2013)^24^ | All patients received chemo prior to study commencement, and 58% received anthracycline only. Since no other studies compare to placebo, this node was pooled with the AC-T node. |
| **AC-TH_9-10wk_** | Anthracycline + taxane-based chemo + trastuzumab IV (9-10 wks) | Docetaxel + trastuzumab (qw for 9 weeks) → fluorouracil + epirubicin + cyclophosphamide | FinHer (Joensuu 2009)^20^ | FinHer investigated trastuzumab at 9 weeks duration. This was pooled with 10 weeks’ duration from E2198 for simplicity. |
|  |  | Paclitaxel + trastuzumab (10 weeks) → doxorubicin + cyclophosphamide | E2198 (Schneider 2015)^19^ | E2198 investigated trastuzumab at 10 weeks duration. This was pooled with 9 weeks’ duration from FinHer for simplicity. |
| **TCH_52wk_** | Docetaxel + carboplatin + trastuzumab IV (52 wks) | Docetaxel + carboplatin + trastuzumab IV (52 wks) | BCIRG 006 (Slamon 2015)^38^ |  |
|  |  | Docetaxel + carboplatin + trastuzumab IV (1 year) | Bayraktar 2012^33^ |  |
| **AC-TH_26wk_** | Anthracycline + taxane-based chemo + trastuzumab IV (26 wks) | Fluorouracil + epirubicin + cyclophosphamide → docetaxel + trastuzumab IV (6 months) | HORG (Mavroudis 2015)^11^ |  |
|  |  | ‘Chemo’ + trastuzumab IV (6 months) | PHARE (Pivot 2013)^15^ | 73% of patients received an anthracycline/taxane-based regimen. |
|  |  | Paclitaxel + trastuzumab (6 months) → FEC | Gonzalez-Angulo 2015^34^ |  |
| **AC-TH_SC,52wk_** | Anthracycline + taxane-based chemo + trastuzumab SC (52 wks) | Docetaxel → fluorouracil + epirubicin + cyclophosphamide + trastuzumab SC (1 year) | HannaH (Jackisch 2016)^10^ |  |
| **AC-TH_52wk_** | Anthracycline + taxane-based chemo + trastuzumab IV (52 wks) | Docetaxel → fluorouracil + epirubicin + cyclophosphamide + trastuzumab IV (1 year) | HannaH (Jackisch 2016)^10^ |  |
|  |  | Fluorouracil + epirubicin + cyclophosphamide → docetaxel + trastuzumab IV (1 year) | HORG (Mavroudis 2015)^11^ |  |
|  |  | ‘Chemo’ + trastuzumab IV (12 months) | PHARE (Pivot 2013)^15^ | 73% of patients received an anthracycline/taxane-based regimen. |
|  |  | Anthracycline or taxane → trastuzumab (1 year) | HERA (Goldhirsch 2013)^2^ | 95% of patients received anthracycline and 25.5% received taxane. Based on this, the HERA trial would be more appropriately displayed as a connection to the AC node; however, the AC node does not exist in all networks. Therefore, the HERA trial was forced to connect to the AC-T vs. AC-TH,52wk main “highway” of the network. |
|  |  | Doxorubicin + cyclophosphamide → docetaxel + trastuzumab IV (1 year) | BCIRG 006 (Slamon 2015)^38^ |  |
|  |  | Doxorubicin + cyclophosphamide → paclitaxel + trastuzumab IV (1 year) | NCCTG N9831 & NSABP B-31 (Perez 2014)^12^ |  |
|  |  | FEC or epirubicin + docetaxel → trastuzumab IV (1 year) | FNCLCC-PACS 04 (Spielmann 2009)^9^ | ~50% of patients received FEC and ~50% received epirubicin + docetaxel. |
|  |  | Paclitaxel + doxorubicin → CMF → trastuzumab IV (1 year) | NOAH (Gianni 2014)^6^ |  |
|  |  | Docetaxel → cyclophosphamide + epirubicin + fluorouracil → trastuzumab IV (1 year) | FinXX (Joensuu 2014)^21^ |  |
|  |  | Paclitaxel + doxorubicin + cyclophosphamide + trastuzumab IV (1 year) | E2198 (Schneider 2015)^19^ |  |
|  |  | Paclitaxel → FEC → trastuzumab IV (1 year) | NeoALTTO (de Azambuja 2014)^13^ |  |
|  |  | ‘Chemo’ → trastuzumab IV (1 year) | ALTTO (Piccart-Gebhart 2016)^5^ | 5% of patients received only taxanes, 40% definitely received anthracyclines + taxanes, and 55% probably received anthracyclines + taxanes. |
|  |  | Doxorubicin + cyclophosphamide → paclitaxel → trastuzumab IV (1 year) | NSABP B-41 (Robidoux 2016)^36^ |  |
|  |  | Anthracycline + taxane chemotherapy + trastuzumab (1 year) | Seferina 2015^35^ |  |
|  |  | Paclitaxel + trastuzumab → FEC + trastuzumab (1 year) | Bayraktar 2012^33^ |  |
|  |  | Paclitaxel + trastuzumab → FEC + trastuzumab (1 year) | Gonzalez-Angulo 2015^34^ |  |
| **No chemo** | No chemotherapy | No chemotherapy (includes radiotherapy and endocrine therapy) | Seferina 2015^35^ | Most patients received no chemotherapy (16.3% received adjuvant chemotherapy, 58% received radiotherapy, 16.3% received (neo)adjuvant chemotherapy, and 43% received adjuvant endocrine therapy) |
| **AC-TL_52wk_** | Anthracycline + taxane-based chemo + lapatinib (52 wks) | Paclitaxel → FEC → lapatinib (1 year) | NeoALTTO (de Azambuja 2014)^13^ |  |
|  |  | ‘Chemo’ → lapatinib (1 year) | ALTTO (Piccart-Gebhart 2016)^5^ | 5% of patients received only taxanes, 40% definitely received anthracyclines + taxanes, and 55% probably received anthracyclines + taxanes. |
|  |  | Lapatinib (1 year) | TEACH (Goss 2013)^24^ | All patients received chemo prior to study commencement, and 58% received anthracycline only. |
| **AC → T-L_12wk_-H_52wk_** | Anthracycline + taxane-based chemo + lapatinib (12 wks) + trastuzumab IV (52 wks) | Doxorubicin + cyclophosphamide → paclitaxel + lapatinib (12 wks) + trastuzumab IV (52 wks) | NSABP B-41 (Robidoux 2016)^36^ |  |
| **AC → T-L_12wk_-H_34wk_** | Anthracycline + taxane-based chemo + lapatinib (12 wks) + trastuzumab IV (34 wks) | Doxorubicin + cyclophosphamide → paclitaxel + lapatinib (12 wks) + trastuzumab IV (34 wks) | NSABP B-41 (Robidoux 2016)^36^ |  |
| **AC-TL_52wk_-H_52wk_** | Anthracycline + taxane-based chemo + lapatinib (52 wks) + trastuzumab IV (52 wks) | Paclitaxel → FEC → Lapatinib (1 year) + trastuzumab IV (1 year) | NeoALTTO (de Azambuja 2014)^13^ |  |
|  |  | ‘Chemo’ → trastuzumab IV (1 year) + lapatinib (1 year) | ALTTO (Piccart-Gebhart 2016)^5^ | 5% of patients received only taxanes, 40% definitely received anthracyclines + taxanes, and 55% probably received anthracyclines + taxanes. |
| **AC-TL_34wk_-H_12wk_** | Anthracycline + taxane-based chemo + trastuzumab IV (12 wks) + lapatinib (34 wks) | ‘Chemo’ → trastuzumab IV (q1w for 12 wks) + lapatinib (34 wks) | ALTTO (Piccart-Gebhart 2016)^5^ | 5% of patients received only taxanes, 40% definitely received anthracyclines + taxanes, and 55% probably received anthracyclines + taxanes. |

*AC* anthracycline (doxorubicin, epirubicin) + cyclophosphamide, *AV* anthracycline + vinorelbine, *CMF* cyclophosphamide + methotrexate + fluorouracil, *Dose dense* AC → T, or AC, either weekly or biweekly, *E* epirubicin, *FEC* fluorouracil + epirubicin + cyclophosphamide, *H* Herceptin® intravenous (IV), *HSC* Herceptin® subcutaneous (SC), *L* lapatinib, *No Chemo* no chemotherapy (includes endocrine therapy and radiotherapy), *No Tx* no treatment, *q2w* once every 2 weeks, *q3w* once every 3 weeks, *qw* weekly, *T* taxane (docetaxel, paclitaxel), *TCH* docetaxel + carboplatin + Herceptin® IV, *V* vinorelbine, *wk* weeks, *X* capecitabine.

Summary of Hazard Ratios for Analyses

Table 6 Summary of hazard ratios for overall survival analyses

| Study | Publication | Treatment 1 | Treatment 2 | Treatment 3 | Treatment 4 | T1 vs. T2,  HR (95% CI) | T1 vs. T3,  HR (95% CI) | T1 vs. T4,  HR (95% CI) |
| --- | --- | --- | --- | --- | --- | --- | --- | --- |
| RCTs with 100% HER2+ patients | | | | | | | | |
| ALTTO | Piccart-Gebhart 2016^5^ | AC-TH,52wk | AC-TL,52wk | AC-TL,52wk-H,52wk | AC-TL,34wk-H,12wk | 0.74 (0.58 to 0.92) | 1.25 (0.97 to 1.61) | 1.10 (0.86 to 1.41) |
| BCIRG 006 | Slamon 2015^38^ | AC-T | AC-TH,52wk | TCH,52wk | NA | 1.59 (1.27 to 1.96) | 1.32 (1.08 to 1.61) | NA |
| BCIRG 006 | Slamon 2011^8^ | AC-T | AC-TH,52wk | TCH,52wk | NA | 1.59 (P < 0.001) | 1.30 (P = 0.04) | NA |
| FNCLCC-PACS 04 | Spielmann 2009^9^ | AC-TH,52wk | AC-T | NA | NA | 1.27 (0.68 to 2.38) | NA | NA |
| HannaH | Jackisch 2016^10^ | AC-THSC,52wk | AC-TH,52wk | NA | NA | 0.76 (0.44 to 1.32) | NA | NA |
| HERA | Goldhirsch 2013^2^ | AC-TH,52wk | AC-TH,104wk | AC-T | NA | 1.05 (0.86 to 1.28) | 0.76 (0.65 to 0.88) | NA |
| HERA | Gianni 2011^4^ | AC-TH,52wk | AC-T | NA | NA | 0.85 (0.70 to 1.04) | NA | NA |
| HERA | Smith 2007^3^ | AC-TH,52wk | AC-T | NA | NA | 0.66 (0.47 to 0.91) | NA | NA |
| HERA | Piccart-Gebhart 2005^39^ | AC-TH,52wk | AC-T | NA | NA | 0.76 (0.47 to 1.23) | NA | NA |
| HORG | Mavroudis 2015^11^ | AC-TH,26wk | AC-TH,52wk | NA | NA | 0.72 (0.30 to 1.73) | NA | NA |
| NCCTG N9831 & NSABP B-31 | Perez 2014^12^ | AC-TH,52wk | AC-T | NA | NA | 0.63 (0.54 to 0.73) | NA | NA |
| NCCTG N9831 & NSABP B-31 | Perez 2011^40^ | AC-TH,52wk | AC-T | NA | NA | 0.61 (0.50 to 0.75) | NA | NA |
| NCCTG N9831 & NSABP B-31 | Romond 2005^41^ | AC-TH,52wk | AC-T | NA | NA | 0.67 (0.48 to 0.93) | NA | NA |
| NeoALTTO | de Azambuja 2014^13^ | AC-TH,52wk | AC-TL,52wk | AC-TL,52wk-H,52wk | NA | 1.16 (0.61 to 2.22) | 1.61 (0.80 to 3.33) | NA |
| NOAH | Gianni 2014^6^ | AC-TH,52wk | AC-T | NA | NA | 0.66 (0.43 to 1.01) | NA | NA |
| NOAH | Gianni 2010^7^ | AC-TH,52wk | AC-T | NA | NA | 0.62 (P = 0.114) | NA | NA |
| NSABP B-41 | Robidoux 2016^36^ | AC-TH,52wk | AC → T-L,12wk-H,34wk | AC → T-L,12wk-H,52wk | NA | 0.66 (0.30 to 1.45) | 1.59 (0.60 to 4.17) | NA |
| PHARE | Pivot 2013^15^ | AC-TH,52wk | AC-TH,26wk | NA | NA | 0.68 (0.49 to 0.93) | NA | NA |
| RCTs with HER2+ subgroups | | | | | | | | |
| BCIRG 001 | Mackey 2013^16^ | AC-T | AC | NA | NA | 0.63 (0.43 to 0.93) | NA | NA |
| Boccardo 2010 | Boccardo 2010^28^ | AC | T → AV | NA | NA | 0.68 (0.32 to 1.45) | NA | NA |
| BR9601 & NEAT | Earl 2012^17^ | AC | CMF | NA | NA | 0.75 (0.49 to 1.17) | NA | NA |
| Colozza 2005 | Colozza 2005^30^ | CMF | E | NA | NA | 0.61 (0.22 to 2.71) | NA | NA |
| Del Mastro 2015 | Del Mastro 2015^26^ | Dose dense | AC-T | NA | NA | 0.70 (0.43 to 1.14) | NA | NA |
| E1199 | Sparano 2008 ^37^ | Dose dense | AC-T | NA | NA | 0.85 (0.46 to 3.13) | NA | NA |
| E2198 | Schneider 2015^19^ | AC-TH,52wk | AC-TH,9-10wk | NA | NA | 1.21 (0.46 to 3.13) | NA | NA |
| FinHer | Joensuu 2009^20^ | AC-TH,9-10wk | AC-T | AC-VH,9wk | AC-V | 0.42 (0.13 to 1.33) | 0.56 (0.17 to 1.85) | 0.37 (0.12 to 1.15) |
| FinXX | Joensuu 2014^21^ | AC-TH,52wk | AC-T | NA | NA | 0.70 (0.37 to 1.32) | NA | NA |
| GeparTrio | Von Minckwitz 2013^22^ | AC-T → VX | AC-T | NA | NA | 0.72 (0.51 to 1.04) | NA | NA |
| GONO-MIG-1 | Del Mastro 2005^23^ | Dose dense | AC | NA | NA | 0.59 (0.26 to 1.37) | NA | NA |
| Miles 1999 | Miles 1999^29^ | CMF | No Tx | NA | NA | 0.62 (0.38 to 1.02) | NA | NA |
| Rocca 2014 | Rocca 2014^27^ | AC | CMF | NA | NA | 0.53 (0.24 to 1.16) | NA | NA |
| TEACH | Goss 2013^24^ | AC-TL,52wk | AC-T | NA | NA | 1.02 (0.75 to 1.39) | NA | NA |
| UNICANCER-PACS-01 | Coudert 2012^25^ | AC-T | AC | NA | NA | 0.50 (0.27 to 0.90) | NA | NA |
| Non-randomized studies | | | | | | | | |
| Bayraktar 2012 | Bayraktar 2012^33^ | AC-TH,52wk | TCH,52wk | NA | NA | 0.37 (0.12 to 1.13) | NA | NA |
| Gonzalez-Angulo 2015 | Gonzalez-Angulo 2015^34^ | AC-TH,52wk | AC-TH,26wk | NA | NA | 0.75 (0.31 to 1.78) | NA | NA |
| Seferina 2015 | Seferina 2015^35^ | AC-TH,52wk | No chemo | NA | NA | 0.48 (0.23 to 1.01) | NA | NA |

*AC* anthracycline (doxorubicin, epirubicin) + cyclophosphamide, *AV* anthracycline + vinorelbine, *CI* confidence interval, *CMF* cyclophosphamide + methotrexate + fluorouracil, *Dose dense* AC → T, or AC, either weekly or biweekly, *E* epirubicin, *H* Herceptin® intravenous (IV), *HER2+* human epidermal growth factor receptor 2-positive, *HR* hazard ratio, *HSC* Herceptin® subcutaneous (SC), *L* lapatinib, *NA* not applicable, *No Chemo* no chemotherapy (includes endocrine therapy and radiotherapy), *No Tx* no treatment, *RCT* randomized controlled trial, *T* taxane (docetaxel, paclitaxel), *TCH* docetaxel + carboplatin + Herceptin® IV, *V* vinorelbine, *wk* weeks, *X* capecitabine.

Complete NMA results for 2016 reference case analysis

The NMA results from the fixed effects and random effects models of the 2016 reference case OS analyses are presented in Tables 7-10. Findings from all possible pairwise comparisons are shown in Table 7 (fixed effects) and Table 9 (random effects), and additional measures of effect for each treatment are shown in Table 8 (fixed effects) and Table 10 (random effects).

Table 7 NMA results of 2016 reference case OS analysis for fixed effects model

| Comparison (T1 vs. T2) | HR of T1 vs. T2 | 95% CrI of HR | Probability of T1 better than T2 | SD of p(better) |  |
| --- | --- | --- | --- | --- | --- |
| AC-TH,52wk vs. AC-T | 0.69 | 0.63 to 0.76 | 1.00 | 0.00 |  |
| TCH,52wk vs. AC-T | 0.76 | 0.63 to 0.93 | 1.00 | 0.06 |  |
| AC-TH,26wk vs. AC-T | 0.94 | 0.68 to 1.28 | 0.66 | 0.47 |  |
| AC-TH,104wk vs. AC-T | 0.67 | 0.54 to 0.83 | 1.00 | 0.01 |  |
| AC-THSC,52wk vs. AC-T | 0.53 | 0.30 to 0.92 | 0.99 | 0.11 |  |
| AC-TL,52wk vs. AC-T | 0.93 | 0.77 to 1.12 | 0.78 | 0.42 |  |
| AC-TL,52wk-H,52wk vs. AC-T | 0.54 | 0.42 to 0.70 | 1.00 | 0.00 |  |
| AC-TL,34wk-H,12wk vs. AC-T | 0.63 | 0.48 to 0.82 | 1.00 | 0.02 |  |
| AC → T-L,12wk-H,52wk vs. AC-T | 0.43 | 0.17 to 1.16 | 0.95 | 0.21 |  |
| AC → T-L,12wk-H,34wk vs. AC-T | 1.05 | 0.47 to 2.33 | 0.45 | 0.50 |  |
| AC-TH,9-10wk vs. AC-T | 0.50 | 0.24 to 1.06 | 0.97 | 0.18 |  |
| AC-T → VX vs. AC-T | 0.72 | 0.51 to 1.03 | 0.96 | 0.19 |  |
| AC-VH,9wk vs. AC-T | 0.90 | 0.22 to 3.64 | 0.56 | 0.50 |  |
| AC-V vs. AC-T | 1.35 | 0.35 to 5.25 | 0.33 | 0.47 |  |
| T → AV vs. AC-T | 2.42 | 1.08 to 5.47 | 0.02 | 0.13 |  |
| Dose Dense vs. AC-T | 0.80 | 0.58 to 1.10 | 0.92 | 0.28 |  |
| CMF vs. AC-T | 2.38 | 1.45 to 3.88 | 0.00 | 0.02 |  |
| AC vs. AC-T | 1.65 | 1.21 to 2.24 | 0.00 | 0.02 |  |
| No Tx vs. AC-T | 3.85 | 1.91 to 7.73 | 0.00 | 0.01 |  |
| E vs. AC-T | 3.90 | 1.04 to 14.78 | 0.02 | 0.15 |  |
| TCH,52wk vs. AC-TH,52wk | 1.10 | 0.89 to 1.37 | 0.19 | 0.39 |  |
| AC-TH,26wk vs. AC-TH,52wk | 1.35 | 1.00 to 1.83 | 0.02 | 0.16 |  |
| AC-TH,104wk vs. AC-TH,52wk | 0.97 | 0.79 to 1.18 | 0.63 | 0.48 |  |
| AC-THSC,52wk vs. AC-TH,52wk | 0.76 | 0.44 to 1.31 | 0.84 | 0.37 |  |
| AC-TL,52wk vs. AC-TH,52wk | 1.34 | 1.12 to 1.61 | 0.00 | 0.02 |  |
| AC-TL,52wk-H,52wk vs. AC-TH,52wk | 0.78 | 0.61 to 0.99 | 0.98 | 0.14 |  |
| AC-TL,34wk-H,12wk vs. AC-TH,52wk | 0.91 | 0.71 to 1.16 | 0.78 | 0.41 |  |
| AC → T-L,12wk-H,52wk vs. AC-TH,52wk | 0.63 | 0.24 to 1.67 | 0.82 | 0.38 |  |
| AC → T-L,12wk-H,34wk vs. AC-TH,52wk | 1.52 | 0.69 to 3.35 | 0.15 | 0.36 |  |
| AC-TH,9-10wk vs. AC-TH,52wk | 0.73 | 0.35 to 1.53 | 0.80 | 0.40 |  |
| AC-T → VX vs. AC-TH,52wk | 1.04 | 0.72 to 1.50 | 0.42 | 0.49 |  |
| AC-VH,9wk vs. AC-TH,52wk | 1.29 | 0.32 to 5.24 | 0.36 | 0.48 |  |
| AC-V vs. AC-TH,52wk | 1.95 | 0.51 to 7.54 | 0.17 | 0.37 |  |
| T → AV vs. AC-TH,52wk | 3.49 | 1.55 to 7.96 | 0.00 | 0.03 |  |
| Dose Dense vs. AC-TH,52wk | 1.15 | 0.83 to 1.61 | 0.20 | 0.40 |  |
| CMF vs. AC-TH,52wk | 3.45 | 2.08 to 5.66 | 0.00 | 0.00 |  |
| AC vs. AC-TH,52wk | 2.38 | 1.73 to 3.29 | 0.00 | 0.00 |  |
| No Tx vs. AC-TH,52wk | 5.55 | 2.74 to 11.24 | 0.00 | 0.00 |  |
| E vs. AC-TH,52wk | 5.63 | 1.49 to 21.39 | 0.01 | 0.07 |  |
| AC-TH,26wk vs. TCH,52wk | 1.23 | 0.85 to 1.78 | 0.14 | 0.34 |  |
| AC-TH,104wk vs. TCH,52wk | 0.88 | 0.66 to 1.17 | 0.81 | 0.39 |  |
| AC-THSC,52wk vs. TCH,52wk | 0.69 | 0.38 to 1.24 | 0.89 | 0.31 |  |
| AC-TL,52wk vs. TCH,52wk | 1.22 | 0.93 to 1.60 | 0.08 | 0.27 |  |
| AC-TL,52wk-H,52wk vs. TCH,52wk | 0.71 | 0.51 to 0.97 | 0.98 | 0.13 |  |
| AC-TL,34wk-H,12wk vs. TCH,52wk | 0.82 | 0.59 to 1.14 | 0.88 | 0.33 |  |
| AC → T-L,12wk-H,52wk vs. TCH,52wk | 0.57 | 0.21 to 1.55 | 0.86 | 0.34 |  |
| AC → T-L,12wk-H,34wk vs. TCH,52wk | 1.38 | 0.61 to 3.14 | 0.22 | 0.42 |  |
| AC-TH,9-10wk vs. TCH,52wk | 0.66 | 0.31 to 1.42 | 0.86 | 0.35 |  |
| AC-T → VX vs. TCH,52wk | 0.94 | 0.63 to 1.42 | 0.61 | 0.49 |  |
| AC-VH,9wk vs. TCH,52wk | 1.17 | 0.28 to 4.86 | 0.41 | 0.49 |  |
| AC-V vs. TCH,52wk | 1.77 | 0.46 to 6.94 | 0.21 | 0.41 |  |
| T → AV vs. TCH,52wk | 3.18 | 1.38 to 7.35 | 0.00 | 0.06 |  |
| Dose Dense vs. TCH,52wk | 1.05 | 0.72 to 1.52 | 0.41 | 0.49 |  |
| CMF vs. TCH,52wk | 3.12 | 1.84 to 5.31 | 0.00 | 0.00 |  |
| AC vs. TCH,52wk | 2.16 | 1.50 to 3.12 | 0.00 | 0.00 |  |
| No Tx vs. TCH,52wk | 5.05 | 2.43 to 10.44 | 0.00 | 0.00 |  |
| E vs. TCH,52wk | 5.11 | 1.34 to 19.70 | 0.01 | 0.09 |  |
| AC-TH,104wk vs. AC-TH,26wk | 0.71 | 0.50 to 1.02 | 0.97 | 0.18 |  |
| AC-THSC,52wk vs. AC-TH,26wk | 0.56 | 0.30 to 1.05 | 0.96 | 0.18 |  |
| AC-TL,52wk vs. AC-TH,26wk | 0.99 | 0.70 to 1.41 | 0.52 | 0.50 |  |
| AC-TL,52wk-H,52wk vs. AC-TH,26wk | 0.57 | 0.39 to 0.84 | 1.00 | 0.05 |  |
| AC-TL,34wk-H,12wk vs. AC-TH,26wk | 0.67 | 0.45 to 0.99 | 0.98 | 0.15 |  |
| AC → T-L,12wk-H,52wk vs. AC-TH,26wk | 0.46 | 0.17 to 1.29 | 0.93 | 0.26 |  |
| AC → T-L,12wk-H,34wk vs. AC-TH,26wk | 1.12 | 0.48 to 2.62 | 0.40 | 0.49 |  |
| AC-TH,9-10wk vs. AC-TH,26wk | 0.53 | 0.24 to 1.20 | 0.94 | 0.25 |  |
| AC-T → VX vs. AC-TH,26wk | 0.77 | 0.48 to 1.24 | 0.86 | 0.35 |  |
| AC-VH,9wk vs. AC-TH,26wk | 0.95 | 0.23 to 4.00 | 0.52 | 0.50 |  |
| AC-V vs. AC-TH,26wk | 1.44 | 0.36 to 5.74 | 0.30 | 0.46 |  |
| T → AV vs. AC-TH,26wk | 2.58 | 1.08 to 6.21 | 0.02 | 0.13 |  |
| Dose Dense vs. AC-TH,26wk | 0.85 | 0.54 to 1.33 | 0.76 | 0.43 |  |
| CMF vs. AC-TH,26wk | 2.55 | 1.42 to 4.55 | 0.00 | 0.03 |  |
| AC vs. AC-TH,26wk | 1.76 | 1.13 to 2.73 | 0.01 | 0.08 |  |
| No Tx vs. AC-TH,26wk | 4.10 | 1.90 to 8.85 | 0.00 | 0.02 |  |
| E vs. AC-TH,26wk | 4.16 | 1.07 to 16.40 | 0.02 | 0.14 |  |
| AC-THSC,52wk vs. AC-TH,104wk | 0.79 | 0.44 to 1.41 | 0.79 | 0.41 |  |
| AC-TL,52wk vs. AC-TH,104wk | 1.39 | 1.07 to 1.82 | 0.01 | 0.09 |  |
| AC-TL,52wk-H,52wk vs. AC-TH,104wk | 0.80 | 0.59 to 1.09 | 0.92 | 0.28 |  |
| AC-TL,34wk-H,12wk vs. AC-TH,104wk | 0.94 | 0.69 to 1.29 | 0.66 | 0.47 |  |
| AC → T-L,12wk-H,52wk vs. AC-TH,104wk | 0.65 | 0.24 to 1.77 | 0.80 | 0.40 |  |
| AC → T-L,12wk-H,34wk vs. AC-TH,104wk | 1.57 | 0.69 to 3.54 | 0.14 | 0.35 |  |
| AC-TH,9-10wk vs. AC-TH,104wk | 0.75 | 0.35 to 1.62 | 0.77 | 0.42 |  |
| AC-T → VX vs. AC-TH,104wk | 1.07 | 0.71 to 1.63 | 0.36 | 0.48 |  |
| AC-VH,9wk vs. AC-TH,104wk | 1.34 | 0.32 to 5.48 | 0.35 | 0.48 |  |
| AC-V vs. AC-TH,104wk | 2.01 | 0.52 to 7.88 | 0.16 | 0.37 |  |
| T → AV vs. AC-TH,104wk | 3.61 | 1.57 to 8.37 | 0.00 | 0.03 |  |
| Dose Dense vs. AC-TH,104wk | 1.19 | 0.81 to 1.75 | 0.18 | 0.39 |  |
| CMF vs. AC-TH,104wk | 3.56 | 2.08 to 6.06 | 0.00 | 0.00 |  |
| AC vs. AC-TH,104wk | 2.46 | 1.70 to 3.57 | 0.00 | 0.00 |  |
| No Tx vs. AC-TH,104wk | 5.75 | 2.75 to 11.89 | 0.00 | 0.00 |  |
| E vs. AC-TH,104wk | 5.81 | 1.52 to 22.53 | 0.00 | 0.07 |  |
| AC-TL,52wk vs. AC-THSC,52wk | 1.77 | 1.00 to 3.15 | 0.03 | 0.16 |  |
| AC-TL,52wk-H,52wk vs. AC-THSC,52wk | 1.02 | 0.56 to 1.87 | 0.47 | 0.50 |  |
| AC-TL,34wk-H,12wk vs. AC-THSC,52wk | 1.19 | 0.66 to 2.17 | 0.28 | 0.45 |  |
| AC → T-L,12wk-H,52wk vs. AC-THSC,52wk | 0.83 | 0.27 to 2.55 | 0.63 | 0.48 |  |
| AC → T-L,12wk-H,34wk vs. AC-THSC,52wk | 1.99 | 0.76 to 5.25 | 0.08 | 0.27 |  |
| AC-TH,9-10wk vs. AC-THSC,52wk | 0.95 | 0.38 to 2.42 | 0.54 | 0.50 |  |
| AC-T → VX vs. AC-THSC,52wk | 1.37 | 0.71 to 2.66 | 0.18 | 0.38 |  |
| AC-VH,9wk vs. AC-THSC,52wk | 1.70 | 0.37 to 7.74 | 0.25 | 0.43 |  |
| AC-V vs. AC-THSC,52wk | 2.56 | 0.60 to 11.06 | 0.10 | 0.30 |  |
| T → AV vs. AC-THSC,52wk | 4.62 | 1.71 to 12.44 | 0.00 | 0.03 |  |
| Dose Dense vs. AC-THSC,52wk | 1.52 | 0.80 to 2.89 | 0.10 | 0.30 |  |
| CMF vs. AC-THSC,52wk | 4.53 | 2.16 to 9.50 | 0.00 | 0.01 |  |
| AC vs. AC-THSC,52wk | 3.13 | 1.66 to 5.91 | 0.00 | 0.02 |  |
| No Tx vs. AC-THSC,52wk | 7.30 | 2.98 to 17.87 | 0.00 | 0.01 |  |
| E vs. AC-THSC,52wk | 7.45 | 1.78 to 31.42 | 0.00 | 0.06 |  |
| AC-TL,52wk-H,52wk vs. AC-TL,52wk | 0.58 | 0.43 to 0.77 | 1.00 | 0.01 |  |
| AC-TL,34wk-H,12wk vs. AC-TL,52wk | 0.68 | 0.50 to 0.91 | 1.00 | 0.07 |  |
| AC → T-L,12wk-H,52wk vs. AC-TL,52wk | 0.47 | 0.18 to 1.27 | 0.93 | 0.25 |  |
| AC → T-L,12wk-H,34wk vs. AC-TL,52wk | 1.13 | 0.50 to 2.55 | 0.38 | 0.49 |  |
| AC-TH,9-10wk vs. AC-TL,52wk | 0.54 | 0.25 to 1.16 | 0.94 | 0.24 |  |
| AC-T → VX vs. AC-TL,52wk | 0.78 | 0.52 to 1.16 | 0.89 | 0.31 |  |
| AC-VH,9wk vs. AC-TL,52wk | 0.96 | 0.23 to 3.97 | 0.52 | 0.50 |  |
| AC-V vs. AC-TL,52wk | 1.45 | 0.37 to 5.68 | 0.30 | 0.46 |  |
| T → AV vs. AC-TL,52wk | 2.60 | 1.14 to 6.02 | 0.01 | 0.11 |  |
| Dose Dense vs. AC-TL,52wk | 0.86 | 0.59 to 1.25 | 0.79 | 0.41 |  |
| CMF vs. AC-TL,52wk | 2.57 | 1.51 to 4.34 | 0.00 | 0.01 |  |
| AC vs. AC-TL,52wk | 1.77 | 1.24 to 2.54 | 0.00 | 0.03 |  |
| No Tx vs. AC-TL,52wk | 4.14 | 2.00 to 8.55 | 0.00 | 0.01 |  |
| E vs. AC-TL,52wk | 4.20 | 1.10 to 16.21 | 0.02 | 0.13 |  |
| AC-TL,34wk-H,12wk vs. AC-TL,52wk-H,52wk | 1.17 | 0.83 to 1.63 | 0.18 | 0.39 |  |
| AC → T-L,12wk-H,52wk vs. AC-TL,52wk-H,52wk | 0.81 | 0.30 to 2.22 | 0.66 | 0.47 |  |
| AC → T-L,12wk-H,34wk vs. AC-TL,52wk-H,52wk | 1.96 | 0.85 to 4.45 | 0.06 | 0.23 |  |
| AC-TH,9-10wk vs. AC-TL,52wk-H,52wk | 0.93 | 0.43 to 2.04 | 0.57 | 0.50 |  |
| AC-T → VX vs. AC-TL,52wk-H,52wk | 1.34 | 0.87 to 2.08 | 0.10 | 0.30 |  |
| AC-VH,9wk vs. AC-TL,52wk-H,52wk | 1.66 | 0.40 to 6.91 | 0.24 | 0.43 |  |
| AC-V vs. AC-TL,52wk-H,52wk | 2.51 | 0.64 to 9.91 | 0.09 | 0.29 |  |
| T → AV vs. AC-TL,52wk-H,52wk | 4.49 | 1.92 to 10.61 | 0.00 | 0.01 |  |
| Dose Dense vs. AC-TL,52wk-H,52wk | 1.48 | 0.98 to 2.23 | 0.03 | 0.17 |  |
| CMF vs. AC-TL,52wk-H,52wk | 4.43 | 2.54 to 7.68 | 0.00 | 0.00 |  |
| AC vs. AC-TL,52wk-H,52wk | 3.06 | 2.05 to 4.57 | 0.00 | 0.00 |  |
| No Tx vs. AC-TL,52wk-H,52wk | 7.14 | 3.40 to 15.10 | 0.00 | 0.00 |  |
| E vs. AC-TL,52wk-H,52wk | 7.24 | 1.88 to 28.33 | 0.00 | 0.04 |  |
| AC → T-L,12wk-H,52wk vs. AC-TL,34wk-H,12wk | 0.69 | 0.25 to 1.89 | 0.76 | 0.43 |  |
| AC → T-L,12wk-H,34wk vs. AC-TL,34wk-H,12wk | 1.67 | 0.73 to 3.82 | 0.11 | 0.31 |  |
| AC-TH,9-10wk vs. AC-TL,34wk-H,12wk | 0.80 | 0.37 to 1.75 | 0.71 | 0.45 |  |
| AC-T → VX vs. AC-TL,34wk-H,12wk | 1.15 | 0.74 to 1.78 | 0.27 | 0.44 |  |
| AC-VH,9wk vs. AC-TL,34wk-H,12wk | 1.43 | 0.34 to 5.90 | 0.31 | 0.46 |  |
| AC-V vs. AC-TL,34wk-H,12wk | 2.15 | 0.54 to 8.53 | 0.14 | 0.35 |  |
| T → AV vs. AC-TL,34wk-H,12wk | 3.86 | 1.64 to 9.07 | 0.00 | 0.03 |  |
| Dose Dense vs. AC-TL,34wk-H,12wk | 1.27 | 0.84 to 1.92 | 0.13 | 0.33 |  |
| CMF vs. AC-TL,34wk-H,12wk | 3.80 | 2.18 to 6.61 | 0.00 | 0.00 |  |
| AC vs. AC-TL,34wk-H,12wk | 2.62 | 1.76 to 3.93 | 0.00 | 0.00 |  |
| No Tx vs. AC-TL,34wk-H,12wk | 6.12 | 2.90 to 12.93 | 0.00 | 0.00 |  |
| E vs. AC-TL,34wk-H,12wk | 6.21 | 1.61 to 24.20 | 0.00 | 0.06 |  |
| AC → T-L,12wk-H,34wk vs. AC → T-L,12wk-H,52wk | 2.41 | 0.69 to 8.40 | 0.08 | 0.28 |  |
| AC-TH,9-10wk vs. AC → T-L,12wk-H,52wk | 1.15 | 0.34 to 3.92 | 0.41 | 0.49 |  |
| AC-T → VX vs. AC → T-L,12wk-H,52wk | 1.65 | 0.58 to 4.66 | 0.17 | 0.38 |  |
| AC-VH,9wk vs. AC → T-L,12wk-H,52wk | 2.06 | 0.37 to 11.28 | 0.21 | 0.40 |  |
| AC-V vs. AC → T-L,12wk-H,52wk | 3.10 | 0.58 to 16.45 | 0.09 | 0.29 |  |
| T → AV vs. AC → T-L,12wk-H,52wk | 5.57 | 1.55 to 19.92 | 0.00 | 0.07 |  |
| Dose Dense vs. AC → T-L,12wk-H,52wk | 1.84 | 0.66 to 5.12 | 0.12 | 0.33 |  |
| CMF vs. AC → T-L,12wk-H,52wk | 5.48 | 1.82 to 16.28 | 0.00 | 0.03 |  |
| AC vs. AC → T-L,12wk-H,52wk | 3.79 | 1.35 to 10.48 | 0.01 | 0.08 |  |
| No Tx vs. AC → T-L,12wk-H,52wk | 8.82 | 2.67 to 29.25 | 0.00 | 0.02 |  |
| E vs. AC → T-L,12wk-H,52wk | 9.02 | 1.72 to 46.42 | 0.00 | 0.07 |  |
| AC-TH,9-10wk vs. AC → T-L,12wk-H,34wk | 0.48 | 0.16 to 1.41 | 0.91 | 0.29 |  |
| AC-T → VX vs. AC → T-L,12wk-H,34wk | 0.69 | 0.29 to 1.64 | 0.80 | 0.40 |  |
| AC-VH,9wk vs. AC → T-L,12wk-H,34wk | 0.86 | 0.17 to 4.30 | 0.58 | 0.49 |  |
| AC-V vs. AC → T-L,12wk-H,34wk | 1.29 | 0.27 to 6.15 | 0.38 | 0.48 |  |
| T → AV vs. AC → T-L,12wk-H,34wk | 2.31 | 0.74 to 7.29 | 0.07 | 0.26 |  |
| Dose Dense vs. AC → T-L,12wk-H,34wk | 0.76 | 0.32 to 1.79 | 0.73 | 0.44 |  |
| CMF vs. AC → T-L,12wk-H,34wk | 2.27 | 0.89 to 5.82 | 0.04 | 0.20 |  |
| AC vs. AC → T-L,12wk-H,34wk | 1.57 | 0.67 to 3.68 | 0.15 | 0.36 |  |
| No Tx vs. AC → T-L,12wk-H,34wk | 3.65 | 1.26 to 10.68 | 0.01 | 0.09 |  |
| E vs. AC → T-L,12wk-H,34wk | 3.73 | 0.79 to 17.58 | 0.05 | 0.21 |  |
| AC-T → VX vs. AC-TH,9-10wk | 1.43 | 0.63 to 3.26 | 0.20 | 0.40 |  |
| AC-VH,9wk vs. AC-TH,9-10wk | 1.78 | 0.53 to 5.91 | 0.17 | 0.38 |  |
| AC-V vs. AC-TH,9-10wk | 2.69 | 0.87 to 8.33 | 0.04 | 0.20 |  |
| T → AV vs. AC-TH,9-10wk | 4.82 | 1.61 to 14.55 | 0.00 | 0.05 |  |
| Dose Dense vs. AC-TH,9-10wk | 1.59 | 0.70 to 3.53 | 0.13 | 0.34 |  |
| CMF vs. AC-TH,9-10wk | 4.74 | 1.95 to 11.50 | 0.00 | 0.02 |  |
| AC vs. AC-TH,9-10wk | 3.28 | 1.47 to 7.31 | 0.00 | 0.05 |  |
| No Tx vs. AC-TH,9-10wk | 7.64 | 2.77 to 21.31 | 0.00 | 0.01 |  |
| E vs. AC-TH,9-10wk | 7.78 | 1.70 to 35.63 | 0.00 | 0.07 |  |
| AC-VH,9wk vs. AC-T → VX | 1.25 | 0.29 to 5.31 | 0.39 | 0.49 |  |
| AC-V vs. AC-T → VX | 1.88 | 0.46 to 7.63 | 0.19 | 0.39 |  |
| T → AV vs. AC-T → VX | 3.37 | 1.39 to 8.17 | 0.00 | 0.06 |  |
| Dose Dense vs. AC-T → VX | 1.11 | 0.69 to 1.78 | 0.34 | 0.47 |  |
| CMF vs. AC-T → VX | 3.31 | 1.80 to 6.07 | 0.00 | 0.01 |  |
| AC vs. AC-T → VX | 2.29 | 1.43 to 3.66 | 0.00 | 0.01 |  |
| No Tx vs. AC-T → VX | 5.35 | 2.44 to 11.67 | 0.00 | 0.00 |  |
| E vs. AC-T → VX | 5.40 | 1.38 to 21.67 | 0.01 | 0.09 |  |
| AC-V vs. AC-VH,9wk | 1.50 | 0.29 to 7.88 | 0.31 | 0.46 |  |
| T → AV vs. AC-VH,9wk | 2.72 | 0.54 to 13.70 | 0.11 | 0.32 |  |
| Dose Dense vs. AC-VH,9wk | 0.89 | 0.21 to 3.77 | 0.56 | 0.50 |  |
| CMF vs. AC-VH,9wk | 2.66 | 0.60 to 11.85 | 0.10 | 0.30 |  |
| AC vs. AC-VH,9wk | 1.84 | 0.44 to 7.80 | 0.20 | 0.40 |  |
| No Tx vs. AC-VH,9wk | 4.30 | 0.88 to 20.80 | 0.04 | 0.18 |  |
| E vs. AC-VH,9wk | 4.38 | 0.63 to 30.80 | 0.07 | 0.25 |  |
| T → AV vs. AC-V | 1.80 | 0.38 to 8.70 | 0.23 | 0.42 |  |
| Dose Dense vs. AC-V | 0.59 | 0.15 to 2.36 | 0.77 | 0.42 |  |
| CMF vs. AC-V | 1.76 | 0.42 to 7.43 | 0.22 | 0.42 |  |
| AC vs. AC-V | 1.22 | 0.31 to 4.87 | 0.39 | 0.49 |  |
| No Tx vs. AC-V | 2.85 | 0.62 to 13.05 | 0.09 | 0.29 |  |
| E vs. AC-V | 2.89 | 0.44 to 19.22 | 0.14 | 0.34 |  |
| Dose Dense vs. T → AV | 0.33 | 0.14 to 0.78 | 0.99 | 0.07 |  |
| CMF vs. T → AV | 0.98 | 0.42 to 2.28 | 0.52 | 0.50 |  |
| AC vs. T → AV | 0.68 | 0.32 to 1.44 | 0.84 | 0.36 |  |
| No Tx vs. T → AV | 1.59 | 0.59 to 4.25 | 0.18 | 0.38 |  |
| E vs. T → AV | 1.62 | 0.36 to 7.20 | 0.27 | 0.44 |  |
| CMF vs. Dose Dense | 2.98 | 1.70 to 5.25 | 0.00 | 0.01 |  |
| AC vs. Dose Dense | 2.06 | 1.36 to 3.12 | 0.00 | 0.02 |  |
| No Tx vs. Dose Dense | 4.81 | 2.28 to 10.21 | 0.00 | 0.01 |  |
| E vs. Dose Dense | 4.88 | 1.25 to 19.09 | 0.01 | 0.11 |  |
| AC vs. CMF | 0.69 | 0.47 to 1.01 | 0.97 | 0.17 |  |
| No Tx vs. CMF | 1.61 | 0.98 to 2.66 | 0.03 | 0.17 |  |
| E vs. CMF | 1.64 | 0.48 to 5.68 | 0.22 | 0.41 |  |
| No Tx vs. AC | 2.33 | 1.24 to 4.38 | 0.00 | 0.06 |  |
| E vs. AC | 2.37 | 0.65 to 8.64 | 0.10 | 0.29 |  |
| E vs. No Tx | 1.02 | 0.27 to 3.85 | 0.49 | 0.50 |  |
| Model Fit Statistics | **DIC = 28.17**  **TotResDev = 33.94 vs. 34.00** | | | | |

*AC* anthracycline (doxorubicin, epirubicin) + cyclophosphamide, *AV* anthracycline + vinorelbine, *CrI* credible interval, *CMF* cyclophosphamide + methotrexate + fluorouracil, *DIC* deviance information criterion, *Dose dense* AC → T, or AC, either weekly or biweekly, *E* epirubicin, *H* Herceptin® intravenous (IV), *HR* hazard ratio, *HSC* Herceptin® subcutaneous (SC), *L* lapatinib, *No Tx* no treatment, *OS* overall survival, *p(better)* probability better, *SD* standard deviation, *T* taxane (docetaxel, paclitaxel), *T1* treatment 1, *T2* treatment 2, *TCH* docetaxel + carboplatin + Herceptin® IV, *TotResDev* total residual deviance, *V* vinorelbine, *wk* weeks, *X* capecitabine.

Table 8 Additional measures of treatment effect for 2016 reference case analysis – fixed effects model

| Treatment | Mean SUCRA | Mean p(best) | Mean Rank (95% CrI) |
| --- | --- | --- | --- |
| AC-TL,52wk-H,52wk | 87.05 | 6.65 | 3 (1 to 7) |
| AC → T-L,12wk-H,52wk | 86.27 | 43.41 | 2 (1 to 15) |
| AC-TH,9-10wk | 84.26 | 21.07 | 3 (1 to 13) |
| AC-THSC,52wk | 84.04 | 15.74 | 3 (1 to 12) |
| AC-TL,34wk-H,12wk | 76.60 | 0.93 | 5 (2 to 11) |
| AC-TH,104wk | 71.33 | 0.16 | 7 (3 to 11) |
| AC-TH,52wk | 68.13 | 0.00 | 7 (5 to 10) |
| AC-T → VX | 64.51 | 0.60 | 8 (2 to 14) |
| TCH,52wk | 59.03 | 0.01 | 9 (5 to 13) |
| Dose dense | 55.78 | 0.08 | 10 (4 to 15) |
| AC-VH,9wk | 52.40 | 8.68 | 12 (1 to 20) |
| AC-TH,26wk | 43.21 | 0.00 | 13 (7 to 16) |
| AC-TL,52wk | 43.13 | 0.00 | 12 (9 to 15) |
| AC → T-L,12wk-H,34wk | 41.83 | 0.90 | 14 (2 to 19) |
| AC-T | 36.99 | 0.00 | 14 (11 to 16) |
| AC-V | 34.86 | 1.72 | 16 (2 to 21) |
| AC | 22.37 | 0.00 | 17 (15 to 18) |
| T → AV | 13.42 | 0.00 | 18 (14 to 21) |
| CMF | 13.33 | 0.00 | 18 (16 to 20) |
| E | 7.17 | 0.04 | 20 (14 to 21) |
| No Tx | 4.31 | 0.00 | 20 (18 to 21) |

*AC* anthracycline (doxorubicin, epirubicin) + cyclophosphamide, *AV* anthracycline + vinorelbine, *CrI* credible interval, *CMF* cyclophosphamide + methotrexate + fluorouracil, *Dose dense* AC → T, or AC, either weekly or biweekly, *E* epirubicin, *H* Herceptin® intravenous (IV), *HSC* Herceptin® subcutaneous (SC), *L* lapatinib, *No Tx* no treatment, *p(best)* probability best, *SUCRA* surface under the cumulative ranking curve, *T* taxane (docetaxel, paclitaxel), *T1* treatment 1, *T2* treatment 2, *TCH* docetaxel + carboplatin + Herceptin® IV, *V*  vinorelbine, *wk* weeks, *X* capecitabine.

Table 9 NMA results of 2016 reference case OS analysis for random effects model

| Comparison (T1 vs. T2) | HR of T1 vs. T2 | 95% CrI of HR | Probability of T1 better than T2 | SD of p(better) |
| --- | --- | --- | --- | --- |
| AC-TH,52wk vs. AC-T | 0.70 | 0.62 to 0.82 | 1.00 | 0.02 |
| TCH,52wk vs. AC-T | 0.77 | 0.59 to 1.06 | 0.96 | 0.21 |
| AC-TH,26wk vs. AC-T | 0.93 | 0.62 to 1.35 | 0.65 | 0.48 |
| AC-TH,104wk vs. AC-T | 0.68 | 0.51 to 0.94 | 0.99 | 0.11 |
| AC-THSC,52wk vs. AC-T | 0.53 | 0.29 to 0.97 | 0.98 | 0.14 |
| AC-TL,52wk vs. AC-T | 0.93 | 0.72 to 1.19 | 0.74 | 0.44 |
| AC-TL,52wk-H,52wk vs. AC-T | 0.54 | 0.39 to 0.76 | 1.00 | 0.03 |
| AC-TL,34wk-H,12wk vs. AC-T | 0.63 | 0.44 to 0.90 | 0.99 | 0.09 |
| AC → T-L,12wk-H,52wk vs. AC-T | 0.43 | 0.15 to 1.21 | 0.94 | 0.24 |
| AC → T-L,12wk-H,34wk vs. AC-T | 1.07 | 0.48 to 2.49 | 0.43 | 0.50 |
| AC-TH,9-10wk vs. AC-T | 0.51 | 0.25 to 1.12 | 0.95 | 0.21 |
| AC-T → VX vs. AC-T | 0.72 | 0.47 to 1.12 | 0.93 | 0.25 |
| AC-VH,9wk vs. AC-T | 0.94 | 0.21 to 3.82 | 0.54 | 0.50 |
| AC-V vs. AC-T | 1.43 | 0.36 to 5.56 | 0.33 | 0.47 |
| T → AV vs. AC-T | 2.40 | 1.02 to 5.66 | 0.02 | 0.15 |
| Dose Dense vs. AC-T | 0.81 | 0.57 to 1.13 | 0.89 | 0.31 |
| CMF vs. AC-T | 2.42 | 1.40 to 4.19 | 0.00 | 0.04 |
| AC vs. AC-T | 1.65 | 1.17 to 2.33 | 0.00 | 0.06 |
| No Tx vs. AC-T | 3.92 | 1.83 to 8.54 | 0.00 | 0.02 |
| E vs. AC-T | 3.72 | 0.95 to 15.38 | 0.03 | 0.17 |
| TCH,52wk vs. AC-TH,52wk | 1.11 | 0.82 to 1.51 | 0.23 | 0.42 |
| AC-TH,26wk vs. AC-TH,52wk | 1.32 | 0.90 to 1.87 | 0.07 | 0.25 |
| AC-TH,104wk vs. AC-TH,52wk | 0.98 | 0.73 to 1.31 | 0.57 | 0.49 |
| AC-THSC,52wk vs. AC-TH,52wk | 0.76 | 0.42 to 1.35 | 0.83 | 0.38 |
| AC-TL,52wk vs. AC-TH,52wk | 1.33 | 1.02 to 1.67 | 0.02 | 0.14 |
| AC-TL,52wk-H,52wk vs. AC-TH,52wk | 0.77 | 0.56 to 1.04 | 0.96 | 0.21 |
| AC-TL,34wk-H,12wk vs. AC-TH,52wk | 0.90 | 0.64 to 1.25 | 0.74 | 0.44 |
| AC → T-L,12wk-H,52wk vs. AC-TH,52wk | 0.62 | 0.21 to 1.73 | 0.81 | 0.39 |
| AC → T-L,12wk-H,34wk vs. AC-TH,52wk | 1.53 | 0.70 to 3.46 | 0.15 | 0.35 |
| AC-TH,9-10wk vs. AC-TH,52wk | 0.73 | 0.35 to 1.58 | 0.78 | 0.42 |
| AC-T → VX vs. AC-TH,52wk | 1.02 | 0.64 to 1.65 | 0.45 | 0.50 |
| AC-VH,9wk vs. AC-TH,52wk | 1.34 | 0.30 to 5.43 | 0.34 | 0.47 |
| AC-V vs. AC-TH,52wk | 2.04 | 0.52 to 7.90 | 0.17 | 0.37 |
| T → AV vs. AC-TH,52wk | 3.44 | 1.43 to 8.12 | 0.00 | 0.05 |
| Dose Dense vs. AC-TH,52wk | 1.15 | 0.79 to 1.66 | 0.23 | 0.42 |
| CMF vs. AC-TH,52wk | 3.47 | 1.95 to 6.03 | 0.00 | 0.01 |
| AC vs. AC-TH,52wk | 2.36 | 1.61 to 3.40 | 0.00 | 0.01 |
| No Tx vs. AC-TH,52wk | 5.60 | 2.57 to 12.11 | 0.00 | 0.01 |
| E vs. AC-TH,52wk | 5.37 | 1.34 to 22.07 | 0.01 | 0.09 |
| AC-TH,26wk vs. TCH,52wk | 1.20 | 0.73 to 1.87 | 0.22 | 0.41 |
| AC-TH,104wk vs. TCH,52wk | 0.88 | 0.58 to 1.33 | 0.75 | 0.43 |
| AC-THSC,52wk vs. TCH,52wk | 0.69 | 0.35 to 1.32 | 0.88 | 0.33 |
| AC-TL,52wk vs. TCH,52wk | 1.20 | 0.81 to 1.71 | 0.16 | 0.37 |
| AC-TL,52wk-H,52wk vs. TCH,52wk | 0.69 | 0.45 to 1.07 | 0.96 | 0.21 |
| AC-TL,34wk-H,12wk vs. TCH,52wk | 0.81 | 0.51 to 1.26 | 0.83 | 0.37 |
| AC → T-L,12wk-H,52wk vs. TCH,52wk | 0.56 | 0.19 to 1.57 | 0.85 | 0.36 |
| AC → T-L,12wk-H,34wk vs. TCH,52wk | 1.39 | 0.59 to 3.33 | 0.22 | 0.42 |
| AC-TH,9-10wk vs. TCH,52wk | 0.66 | 0.30 to 1.50 | 0.83 | 0.37 |
| AC-T → VX vs. TCH,52wk | 0.93 | 0.54 to 1.56 | 0.62 | 0.48 |
| AC-VH,9wk vs. TCH,52wk | 1.21 | 0.26 to 5.02 | 0.40 | 0.49 |
| AC-V vs. TCH,52wk | 1.83 | 0.45 to 7.32 | 0.22 | 0.41 |
| T → AV vs. TCH,52wk | 3.08 | 1.24 to 7.61 | 0.01 | 0.09 |
| Dose Dense vs. TCH,52wk | 1.04 | 0.65 to 1.62 | 0.43 | 0.49 |
| CMF vs. TCH,52wk | 3.13 | 1.66 to 5.80 | 0.00 | 0.03 |
| AC vs. TCH,52wk | 2.13 | 1.34 to 3.33 | 0.00 | 0.05 |
| No Tx vs. TCH,52wk | 5.03 | 2.22 to 11.50 | 0.00 | 0.02 |
| E vs. TCH,52wk | 4.83 | 1.18 to 20.43 | 0.01 | 0.11 |
| AC-TH,104wk vs. AC-TH,26wk | 0.73 | 0.47 to 1.20 | 0.90 | 0.29 |
| AC-THSC,52wk vs. AC-TH,26wk | 0.57 | 0.29 to 1.15 | 0.94 | 0.23 |
| AC-TL,52wk vs. AC-TH,26wk | 1.00 | 0.65 to 1.54 | 0.49 | 0.50 |
| AC-TL,52wk-H,52wk vs. AC-TH,26wk | 0.58 | 0.37 to 0.95 | 0.98 | 0.13 |
| AC-TL,34wk-H,12wk vs. AC-TH,26wk | 0.68 | 0.42 to 1.12 | 0.94 | 0.24 |
| AC → T-L,12wk-H,52wk vs. AC-TH,26wk | 0.47 | 0.15 to 1.40 | 0.91 | 0.29 |
| AC → T-L,12wk-H,34wk vs. AC-TH,26wk | 1.17 | 0.49 to 2.87 | 0.37 | 0.48 |
| AC-TH,9-10wk vs. AC-TH,26wk | 0.56 | 0.25 to 1.29 | 0.91 | 0.29 |
| AC-T → VX vs. AC-TH,26wk | 0.78 | 0.44 to 1.40 | 0.81 | 0.39 |
| AC-VH,9wk vs. AC-TH,26wk | 1.02 | 0.22 to 4.29 | 0.49 | 0.50 |
| AC-V vs. AC-TH,26wk | 1.53 | 0.37 to 6.17 | 0.29 | 0.46 |
| T → AV vs. AC-TH,26wk | 2.60 | 1.02 to 6.85 | 0.02 | 0.15 |
| Dose Dense vs. AC-TH,26wk | 0.87 | 0.52 to 1.48 | 0.70 | 0.46 |
| CMF vs. AC-TH,26wk | 2.61 | 1.36 to 5.17 | 0.00 | 0.06 |
| AC vs. AC-TH,26wk | 1.78 | 1.07 to 3.07 | 0.01 | 0.12 |
| No Tx vs. AC-TH,26wk | 4.20 | 1.79 to 10.08 | 0.00 | 0.03 |
| E vs. AC-TH,26wk | 4.09 | 0.97 to 18.10 | 0.03 | 0.17 |
| AC-THSC,52wk vs. AC-TH,104wk | 0.78 | 0.40 to 1.49 | 0.78 | 0.41 |
| AC-TL,52wk vs. AC-TH,104wk | 1.36 | 0.92 to 1.94 | 0.05 | 0.22 |
| AC-TL,52wk-H,52wk vs. AC-TH,104wk | 0.79 | 0.51 to 1.20 | 0.87 | 0.33 |
| AC-TL,34wk-H,12wk vs. AC-TH,104wk | 0.92 | 0.59 to 1.43 | 0.65 | 0.48 |
| AC → T-L,12wk-H,52wk vs. AC-TH,104wk | 0.63 | 0.21 to 1.85 | 0.79 | 0.41 |
| AC → T-L,12wk-H,34wk vs. AC-TH,104wk | 1.58 | 0.67 to 3.77 | 0.15 | 0.35 |
| AC-TH,9-10wk vs. AC-TH,104wk | 0.75 | 0.34 to 1.70 | 0.74 | 0.44 |
| AC-T → VX vs. AC-TH,104wk | 1.05 | 0.62 to 1.76 | 0.42 | 0.49 |
| AC-VH,9wk vs. AC-TH,104wk | 1.36 | 0.30 to 5.85 | 0.33 | 0.47 |
| AC-V vs. AC-TH,104wk | 2.08 | 0.52 to 8.24 | 0.17 | 0.37 |
| T → AV vs. AC-TH,104wk | 3.48 | 1.41 to 8.71 | 0.00 | 0.06 |
| Dose Dense vs. AC-TH,104wk | 1.18 | 0.74 to 1.85 | 0.23 | 0.42 |
| CMF vs. AC-TH,104wk | 3.52 | 1.86 to 6.61 | 0.00 | 0.02 |
| AC vs. AC-TH,104wk | 2.43 | 1.52 to 3.77 | 0.00 | 0.03 |
| No Tx vs. AC-TH,104wk | 5.72 | 2.51 to 13.00 | 0.00 | 0.02 |
| E vs. AC-TH,104wk | 5.50 | 1.36 to 23.32 | 0.01 | 0.09 |
| AC-TL,52wk vs. AC-THSC,52wk | 1.73 | 0.92 to 3.30 | 0.04 | 0.20 |
| AC-TL,52wk-H,52wk vs. AC-THSC,52wk | 1.00 | 0.53 to 1.99 | 0.50 | 0.50 |
| AC-TL,34wk-H,12wk vs. AC-THSC,52wk | 1.19 | 0.60 to 2.34 | 0.31 | 0.46 |
| AC → T-L,12wk-H,52wk vs. AC-THSC,52wk | 0.82 | 0.24 to 2.58 | 0.63 | 0.48 |
| AC → T-L,12wk-H,34wk vs. AC-THSC,52wk | 2.02 | 0.76 to 5.66 | 0.08 | 0.28 |
| AC-TH,9-10wk vs. AC-THSC,52wk | 0.96 | 0.38 to 2.54 | 0.54 | 0.50 |
| AC-T → VX vs. AC-THSC,52wk | 1.35 | 0.63 to 2.83 | 0.21 | 0.41 |
| AC-VH,9wk vs. AC-THSC,52wk | 1.76 | 0.35 to 8.12 | 0.23 | 0.42 |
| AC-V vs. AC-THSC,52wk | 2.64 | 0.62 to 11.33 | 0.10 | 0.30 |
| T → AV vs. AC-THSC,52wk | 4.51 | 1.57 to 12.68 | 0.00 | 0.05 |
| Dose Dense vs. AC-THSC,52wk | 1.51 | 0.75 to 3.00 | 0.12 | 0.32 |
| CMF vs. AC-THSC,52wk | 4.53 | 2.04 to 10.70 | 0.00 | 0.02 |
| AC vs. AC-THSC,52wk | 3.10 | 1.55 to 6.27 | 0.00 | 0.03 |
| No Tx vs. AC-THSC,52wk | 7.29 | 2.77 to 19.80 | 0.00 | 0.01 |
| E vs. AC-THSC,52wk | 7.08 | 1.56 to 34.96 | 0.01 | 0.07 |
| AC-TL,52wk-H,52wk vs. AC-TL,52wk | 0.58 | 0.41 to 0.84 | 1.00 | 0.05 |
| AC-TL,34wk-H,12wk vs. AC-TL,52wk | 0.68 | 0.47 to 0.99 | 0.98 | 0.15 |
| AC → T-L,12wk-H,52wk vs. AC-TL,52wk | 0.47 | 0.16 to 1.34 | 0.91 | 0.29 |
| AC → T-L,12wk-H,34wk vs. AC-TL,52wk | 1.16 | 0.52 to 2.72 | 0.36 | 0.48 |
| AC-TH,9-10wk vs. AC-TL,52wk | 0.55 | 0.26 to 1.25 | 0.91 | 0.28 |
| AC-T → VX vs. AC-TL,52wk | 0.78 | 0.47 to 1.27 | 0.85 | 0.36 |
| AC-VH,9wk vs. AC-TL,52wk | 1.00 | 0.22 to 4.26 | 0.49 | 0.50 |
| AC-V vs. AC-TL,52wk | 1.53 | 0.38 to 6.12 | 0.29 | 0.46 |
| T → AV vs. AC-TL,52wk | 2.59 | 1.06 to 6.37 | 0.02 | 0.14 |
| Dose Dense vs. AC-TL,52wk | 0.87 | 0.57 to 1.33 | 0.75 | 0.43 |
| CMF vs. AC-TL,52wk | 2.62 | 1.43 to 4.69 | 0.00 | 0.03 |
| AC vs. AC-TL,52wk | 1.78 | 1.16 to 2.78 | 0.01 | 0.08 |
| No Tx vs. AC-TL,52wk | 4.23 | 1.90 to 9.55 | 0.00 | 0.02 |
| E vs. AC-TL,52wk | 4.02 | 1.00 to 17.19 | 0.03 | 0.16 |
| AC-TL,34wk-H,12wk vs. AC-TL,52wk-H,52wk | 1.17 | 0.78 to 1.75 | 0.22 | 0.42 |
| AC → T-L,12wk-H,52wk vs. AC-TL,52wk-H,52wk | 0.80 | 0.27 to 2.39 | 0.65 | 0.48 |
| AC → T-L,12wk-H,34wk vs. AC-TL,52wk-H,52wk | 2.02 | 0.86 to 4.75 | 0.05 | 0.23 |
| AC-TH,9-10wk vs. AC-TL,52wk-H,52wk | 0.95 | 0.43 to 2.14 | 0.55 | 0.50 |
| AC-T → VX vs. AC-TL,52wk-H,52wk | 1.33 | 0.77 to 2.39 | 0.14 | 0.35 |
| AC-VH,9wk vs. AC-TL,52wk-H,52wk | 1.72 | 0.37 to 7.35 | 0.23 | 0.42 |
| AC-V vs. AC-TL,52wk-H,52wk | 2.63 | 0.65 to 10.15 | 0.09 | 0.29 |
| T → AV vs. AC-TL,52wk-H,52wk | 4.48 | 1.79 to 11.29 | 0.00 | 0.03 |
| Dose Dense vs. AC-TL,52wk-H,52wk | 1.49 | 0.92 to 2.39 | 0.05 | 0.22 |
| CMF vs. AC-TL,52wk-H,52wk | 4.50 | 2.36 to 8.44 | 0.00 | 0.01 |
| AC vs. AC-TL,52wk-H,52wk | 3.06 | 1.90 to 4.97 | 0.00 | 0.01 |
| No Tx vs. AC-TL,52wk-H,52wk | 7.22 | 3.15 to 16.65 | 0.00 | 0.01 |
| E vs. AC-TL,52wk-H,52wk | 7.02 | 1.69 to 30.57 | 0.00 | 0.06 |
| AC → T-L,12wk-H,52wk vs. AC-TL,34wk-H,12wk | 0.68 | 0.23 to 2.02 | 0.75 | 0.44 |
| AC → T-L,12wk-H,34wk vs. AC-TL,34wk-H,12wk | 1.70 | 0.72 to 4.18 | 0.11 | 0.31 |
| AC-TH,9-10wk vs. AC-TL,34wk-H,12wk | 0.81 | 0.37 to 1.86 | 0.68 | 0.47 |
| AC-T → VX vs. AC-TL,34wk-H,12wk | 1.14 | 0.65 to 1.97 | 0.31 | 0.46 |
| AC-VH,9wk vs. AC-TL,34wk-H,12wk | 1.48 | 0.32 to 6.40 | 0.30 | 0.46 |
| AC-V vs. AC-TL,34wk-H,12wk | 2.26 | 0.56 to 8.94 | 0.14 | 0.35 |
| T → AV vs. AC-TL,34wk-H,12wk | 3.82 | 1.47 to 9.59 | 0.00 | 0.05 |
| Dose Dense vs. AC-TL,34wk-H,12wk | 1.28 | 0.78 to 2.09 | 0.15 | 0.36 |
| CMF vs. AC-TL,34wk-H,12wk | 3.83 | 1.99 to 7.27 | 0.00 | 0.01 |
| AC vs. AC-TL,34wk-H,12wk | 2.62 | 1.60 to 4.25 | 0.00 | 0.02 |
| No Tx vs. AC-TL,34wk-H,12wk | 6.23 | 2.70 to 14.41 | 0.00 | 0.01 |
| E vs. AC-TL,34wk-H,12wk | 5.93 | 1.47 to 25.93 | 0.01 | 0.08 |
| AC → T-L,12wk-H,34wk vs. AC → T-L,12wk-H,52wk | 2.49 | 0.71 to 8.56 | 0.07 | 0.26 |
| AC-TH,9-10wk vs. AC → T-L,12wk-H,52wk | 1.17 | 0.32 to 4.56 | 0.41 | 0.49 |
| AC-T → VX vs. AC → T-L,12wk-H,52wk | 1.64 | 0.55 to 5.32 | 0.19 | 0.39 |
| AC-VH,9wk vs. AC → T-L,12wk-H,52wk | 2.14 | 0.34 to 12.19 | 0.20 | 0.40 |
| AC-V vs. AC → T-L,12wk-H,52wk | 3.11 | 0.59 to 19.95 | 0.10 | 0.30 |
| T → AV vs. AC → T-L,12wk-H,52wk | 5.53 | 1.40 to 21.98 | 0.01 | 0.08 |
| Dose Dense vs. AC → T-L,12wk-H,52wk | 1.86 | 0.63 to 5.53 | 0.14 | 0.35 |
| CMF vs. AC → T-L,12wk-H,52wk | 5.63 | 1.68 to 17.87 | 0.00 | 0.04 |
| AC vs. AC → T-L,12wk-H,52wk | 3.82 | 1.25 to 11.71 | 0.01 | 0.08 |
| No Tx vs. AC → T-L,12wk-H,52wk | 9.10 | 2.59 to 31.80 | 0.00 | 0.02 |
| E vs. AC → T-L,12wk-H,52wk | 8.53 | 1.66 to 51.27 | 0.01 | 0.07 |
| AC-TH,9-10wk vs. AC → T-L,12wk-H,34wk | 0.47 | 0.16 to 1.48 | 0.90 | 0.30 |
| AC-T → VX vs. AC → T-L,12wk-H,34wk | 0.66 | 0.26 to 1.63 | 0.80 | 0.40 |
| AC-VH,9wk vs. AC → T-L,12wk-H,34wk | 0.87 | 0.17 to 4.21 | 0.57 | 0.49 |
| AC-V vs. AC → T-L,12wk-H,34wk | 1.27 | 0.28 to 6.62 | 0.39 | 0.49 |
| T → AV vs. AC → T-L,12wk-H,34wk | 2.22 | 0.64 to 7.40 | 0.11 | 0.31 |
| Dose Dense vs. AC → T-L,12wk-H,34wk | 0.76 | 0.30 to 1.79 | 0.75 | 0.44 |
| CMF vs. AC → T-L,12wk-H,34wk | 2.25 | 0.82 to 5.82 | 0.05 | 0.23 |
| AC vs. AC → T-L,12wk-H,34wk | 1.53 | 0.59 to 3.67 | 0.18 | 0.38 |
| No Tx vs. AC → T-L,12wk-H,34wk | 3.61 | 1.19 to 10.77 | 0.01 | 0.11 |
| E vs. AC → T-L,12wk-H,34wk | 3.54 | 0.75 to 17.19 | 0.06 | 0.24 |
| AC-T → VX vs. AC-TH,9-10wk | 1.39 | 0.59 to 3.41 | 0.24 | 0.43 |
| AC-VH,9wk vs. AC-TH,9-10wk | 1.84 | 0.48 to 6.35 | 0.17 | 0.38 |
| AC-V vs. AC-TH,9-10wk | 2.68 | 0.87 to 8.06 | 0.04 | 0.20 |
| T → AV vs. AC-TH,9-10wk | 4.67 | 1.45 to 15.47 | 0.00 | 0.06 |
| Dose Dense vs. AC-TH,9-10wk | 1.58 | 0.68 to 3.55 | 0.16 | 0.37 |
| CMF vs. AC-TH,9-10wk | 4.67 | 1.85 to 12.15 | 0.00 | 0.02 |
| AC vs. AC-TH,9-10wk | 3.18 | 1.40 to 7.27 | 0.00 | 0.06 |
| No Tx vs. AC-TH,9-10wk | 7.46 | 2.57 to 22.99 | 0.00 | 0.01 |
| E vs. AC-TH,9-10wk | 7.43 | 1.50 to 37.12 | 0.01 | 0.08 |
| AC-VH,9wk vs. AC-T → VX | 1.30 | 0.28 to 5.80 | 0.36 | 0.48 |
| AC-V vs. AC-T → VX | 1.97 | 0.47 to 7.96 | 0.19 | 0.39 |
| T → AV vs. AC-T → VX | 3.30 | 1.31 to 8.92 | 0.01 | 0.08 |
| Dose Dense vs. AC-T → VX | 1.12 | 0.65 to 1.95 | 0.34 | 0.47 |
| CMF vs. AC-T → VX | 3.36 | 1.68 to 6.78 | 0.00 | 0.03 |
| AC vs. AC-T → VX | 2.29 | 1.34 to 3.99 | 0.00 | 0.05 |
| No Tx vs. AC-T → VX | 5.39 | 2.28 to 13.47 | 0.00 | 0.02 |
| E vs. AC-T → VX | 5.22 | 1.28 to 23.28 | 0.01 | 0.10 |
| AC-V vs. AC-VH,9wk | 1.52 | 0.28 to 8.04 | 0.30 | 0.46 |
| T → AV vs. AC-VH,9wk | 2.57 | 0.49 to 14.23 | 0.13 | 0.34 |
| Dose Dense vs. AC-VH,9wk | 0.87 | 0.20 to 3.99 | 0.58 | 0.49 |
| CMF vs. AC-VH,9wk | 2.58 | 0.56 to 13.07 | 0.11 | 0.31 |
| AC vs. AC-VH,9wk | 1.74 | 0.41 to 8.46 | 0.22 | 0.41 |
| No Tx vs. AC-VH,9wk | 4.12 | 0.83 to 22.79 | 0.04 | 0.20 |
| E vs. AC-VH,9wk | 4.00 | 0.57 to 33.74 | 0.09 | 0.29 |
| T → AV vs. AC-V | 1.72 | 0.33 to 8.94 | 0.25 | 0.43 |
| Dose Dense vs. AC-V | 0.57 | 0.14 to 2.36 | 0.77 | 0.42 |
| CMF vs. AC-V | 1.69 | 0.39 to 7.40 | 0.23 | 0.42 |
| AC vs. AC-V | 1.16 | 0.28 to 4.73 | 0.42 | 0.49 |
| No Tx vs. AC-V | 2.77 | 0.57 to 13.57 | 0.10 | 0.30 |
| E vs. AC-V | 2.76 | 0.37 to 21.56 | 0.17 | 0.37 |
| Dose Dense vs. T → AV | 0.34 | 0.14 to 0.82 | 0.99 | 0.10 |
| CMF vs. T → AV | 1.02 | 0.41 to 2.36 | 0.49 | 0.50 |
| AC vs. T → AV | 0.69 | 0.31 to 1.49 | 0.83 | 0.38 |
| No Tx vs. T → AV | 1.63 | 0.57 to 4.46 | 0.17 | 0.38 |
| E vs. T → AV | 1.61 | 0.33 to 7.50 | 0.29 | 0.45 |
| CMF vs. Dose Dense | 2.99 | 1.64 to 5.56 | 0.00 | 0.02 |
| AC vs. Dose Dense | 2.06 | 1.32 to 3.20 | 0.00 | 0.04 |
| No Tx vs. Dose Dense | 4.82 | 2.17 to 10.93 | 0.00 | 0.02 |
| E vs. Dose Dense | 4.65 | 1.19 to 19.49 | 0.01 | 0.11 |
| AC vs. CMF | 0.69 | 0.45 to 1.05 | 0.96 | 0.19 |
| No Tx vs. CMF | 1.64 | 0.94 to 2.78 | 0.04 | 0.21 |
| E vs. CMF | 1.57 | 0.46 to 5.71 | 0.25 | 0.43 |
| No Tx vs. AC | 2.37 | 1.20 to 4.68 | 0.01 | 0.09 |
| E vs. AC | 2.27 | 0.62 to 9.04 | 0.11 | 0.32 |
| E vs. No Tx | 0.97 | 0.24 to 3.93 | 0.52 | 0.50 |
| Model Fit Statistics | **DIC = 28.17**  **TotResDev = 32.07 vs. 34.00**  **Heterogeneity SD = 0.08 (0.01 to 0.27)** | | | |

*AC* anthracycline (doxorubicin, epirubicin) + cyclophosphamide, *AV* anthracycline + vinorelbine, *CrI* credible interval, *CMF* cyclophosphamide + methotrexate + fluorouracil, *DIC* deviance information criterion, *Dose dense* AC → T, or AC, either weekly or biweekly, *E* epirubicin, *H* Herceptin® intravenous (IV), *HR* hazard ratio, *HSC* Herceptin® subcutaneous (SC), *L* lapatinib, *No Tx* no treatment, *OS* overall survival, *p(better)* probability better, *SD* standard deviation, *T* taxane (docetaxel, paclitaxel), *T1* treatment 1, *T2* treatment 2, *TCH* docetaxel + carboplatin + Herceptin® IV, *TotResDev* total residual deviance, *V* vinorelbine, *wk* weeks, *X* capecitabine.

Table 10 Additional measures of treatment effect for 2016 reference case analysis – random effects model

| Treatment | Mean SUCRA | Mean p(best) | Mean Rank (95% CrI) |
| --- | --- | --- | --- |
| AC-TL,52wk-H,52wk | 86.34 | 7.14 | 3 (1 to 9) |
| AC → T-L,12wk-H,52wk | 85.49 | 43.31 | 2 (1 to 15) |
| AC-THSC,52wk | 83.28 | 14.57 | 3 (1 to 13) |
| AC-TH,9-10wk | 82.91 | 21.36 | 3 (1 to 13) |
| AC-TL,34wk-H,12wk | 76.07 | 1.65 | 5 (2 to 12) |
| AC-TH,104wk | 70.07 | 0.48 | 7 (3 to 12) |
| AC-TH,52wk | 68.25 | 0.00 | 7 (4 to 10) |
| AC-T → VX | 65.38 | 1.17 | 8 (2 to 15) |
| TCH,52wk | 58.96 | 0.10 | 9 (4 to 14) |
| Dose Dense | 56.31 | 0.14 | 10 (4 to 15) |
| AC-VH,9wk | 50.88 | 8.20 | 12 (1 to 20) |
| AC-TH,26wk | 45.60 | 0.02 | 12 (5 to 16) |
| AC-TL,52wk | 44.56 | 0.00 | 12 (8 to 16) |
| AC → T-L,12wk-H,34wk | 40.79 | 0.55 | 14 (3 to 19) |
| AC-T | 38.12 | 0.00 | 13 (11 to 16) |
| AC-V | 34.43 | 1.27 | 16 (2 to 21) |
| AC | 22.65 | 0.00 | 17 (14 to 18) |
| T → AV | 14.16 | 0.00 | 18 (14 to 21) |
| CMF | 13.18 | 0.00 | 18 (16 to 20) |
| E | 8.24 | 0.05 | 20 (13 to 21) |
| No Tx | 4.33 | 0.00 | 20 (18 to 21) |

*AC* anthracycline (doxorubicin, epirubicin) + cyclophosphamide, *AV* anthracycline + vinorelbine, *CrI* credible interval, *CMF* cyclophosphamide + methotrexate + fluorouracil, *Dose dense* AC → T, or AC, either weekly or biweekly, *E* epirubicin, *H* Herceptin® intravenous (IV), *HSC* Herceptin® subcutaneous (SC), *L* lapatinib, *No Tx* no treatment, *p(best)* probability best, *SUCRA* surface under the cumulative ranking curve, *T* taxane (docetaxel, paclitaxel), *T1* treatment 1, *T2* treatment 2, *TCH* docetaxel + carboplatin + Herceptin® IV, *V* vinorelbine, *wk* weeks, *X* capecitabine.

Summary of Model Fit Statistics for Sensitivity Analyses

Table 11 Model fit statistics

| Year | Fixed effects | Random effects |
| --- | --- | --- |
| Sensitivity Analysis #1: RCTs with 100% HER2+ patients | | |
| 2008 | DIC = -1.45  TotResDev = 1 vs. 2 | DIC = 0.06  TotResDev = 1.75 vs. 2  Heterogeneity SD (95% CI) = 0.67 (0.02 to 4.53) |
| 2010 | DIC = 3.51  TotResDev = 4.62 vs. 4 | DIC = 4.41  TotResDev = 3.63 vs. 4  Heterogeneity SD (95% CI) = 0.36 (0.02 to 2.39) |
| 2012 | DIC = 10.49  TotResDev = 10.28 vs. 6 | DIC = 7.67  TotResDev = 5.07 vs. 6  Heterogeneity SD (95% CI) = 0.37 (0.05 to 1.7) |
| 2014 | DIC = 7.58  TotResDev = 12.58 vs. 10 | DIC = 7.24  TotResDev = 9.83 vs. 10  Heterogeneity SD (95% CI) = 0.22 (0.02 to 1.15) |
| 2016 | DIC = 5.98  TotResDev = 21.66 vs. 17 | DIC = 5.96  TotResDev = 18.24 vs. 17  Heterogeneity SD (95% CI) = 0.15 (0.01 to 0.51) |
| Sensitivity Analysis #2: RCTs with 100% HER2+ patients, HER2+ subgroups,  and non-randomized studies (naïve pooling) | | |
| 2008 | DIC = 1.69  TotResDev = 2.99 vs. 4 | DIC = 3.19  TotResDev = 3.74 vs. 4  Heterogeneity SD (95% CI) = 0.68 (0.01 to 4.55) |
| 2010 | DIC = 17  TotResDev = 10.63 vs. 10 | DIC = 17.96  TotResDev = 9.66 vs. 10  Heterogeneity SD (95% CI) = 0.37 (0.02 to 2.41) |
| 2012 | DIC = 30.88  TotResDev = 21.11 vs. 17 | DIC = 28.49  TotResDev = 15.86 vs. 17  Heterogeneity SD (95% CI) = 0.29 (0.03 to 1.05) |
| 2014 | DIC = 27.31  TotResDev = 27.02 vs. 26 | DIC = 27.55  TotResDev = 24.44 vs. 26  Heterogeneity SD (95% CI) = 0.12 (0.01 to 0.42) |
| 2016 | DIC = 27.48  TotResDev = 37.29 vs. 37 | DIC = 27.48  TotResDev = 35.28 vs. 37  Heterogeneity SD (95% CI) = 0.08 (0.01 to 0.26) |
| Sensitivity Analysis #3: RCTs with 100% HER2+ patients, HER2+ subgroups,  and non-randomized studies (Bayesian hierarchical model) | | |
| NA | | |
| Sensitivity Analysis #4: RCTs with 100% HER2+ patients and HER2+ subgroups,  using whole survival curves | | |
| 2008 | DIC = 123.51  TotResDev = 26.17 vs. 28 | DIC = 133.65  TotResDev = 27.52 vs. 28  Heterogeneity SD (95% CI) = 0.30 (0.05 to 2.93) |
| 2010 | DIC = 173.47  TotResDev = 91.38 vs. 118 | DIC = 190.87  TotResDev = 85.52 vs. 118  Heterogeneity SD (95% CI) = 0.27 (0.04 to 3.25) |
| 2012 | DIC = 359.84  TotResDev = 228.64 vs. 228 | DIC = 425.93  TotResDev = 229.93 vs. 228  Heterogeneity SD (95% CI) = 0.19 (0.04 to 1.16) |
| 2014 | DIC = 579.04  TotResDev = 305.22 vs. 306 | DIC = 682.14  TotResDev = 305.84 vs. 306  Heterogeneity SD (95% CI) = 0.17 (0.04 to 0.89) |
| 2016 | DIC = 616.77  TotResDev = 427.78 vs. 429 | DIC = 740.07  TotResDev = 431.08 vs. 429  Heterogeneity SD (95% CI) = 0.15 (0.04 to 0.66) |

*CI* confidence interval, *DIC* deviance information criterion, *NA* not available, *SD* standard deviation, *TotResDev* total residual deviance.

Sensitivity Analyses

The following sensitivity analyses were considered to assess the robustness of the primary cumulative NMA (cNMA) of the reference case:

1. cNMA including only RCTs with 100% HER2+ patients
2. cNMA including RCTs with 100% HER2+ patients, RCTs with HER2+ subgroups, and non-randomized studies – Naïve pooling
3. cNMA including RCTs with 100% HER2+ patients, RCTs with HER2+ subgroups, and non-randomized studies – Bayesian hierarchical model
4. cNMA of the reference case (RCTs with 100% HER2+ patients, and RCTs with HER2+ subgroups) using whole survival curves

Unfortunately, the structure of the evidence networks prevented performing sensitivity analysis #3 using the Bayesian hierarchical model. Based on the methods outlined by Schmitz et al.,^42^ the structure of the evidence networks impeded our ability to estimate the necessary parameters for the model, including bias and variance adjustment factors, since there were only three non-randomized studies available, and one of these comparisons did not contain RCT evidence. The model could have potentially been implemented if the “No chemo” node was collapsed into another node that contained RCT data; however, this was not possible while maintaining that importantly different therapeutic doses were separated into distinct nodes. Therefore, results from sensitivity analysis #3 are unavailable.

Findings from sensitivity analyses #1, #2, and #4 compared with the reference case analysis are summarized in Table 12. Table 11 of this appendix and Table 3 of the main text provide a summary of model fit statistics from these analyses.

Table 12 Summary of findings from reference case NMA and sensitivity analyses

|  | **Reference Case: RCTs with 100% HER2+ patients & RCTs with HER2+ subgroups** | | **SA#1: RCTs with 100% HER2+ patients only** | | **SA#2: Inclusion of NRS (naïve pooling)** | | **SA#4: Analysis using survival curves of reference case** | |
| --- | --- | --- | --- | --- | --- | --- | --- | --- |
| **Year** | **FE** | **RE** | **FE** | **RE** | **FE** | **RE** | **FE** | **RE** |
| **AC-TH_52wk_ vs. AC-T** | | | | | | | | |
| **2008** | 0.66 (0.53, 0.84) | 0.66 (0.03, 12.27) | 0.66 (0.53, 0.84) | 0.67 (0.04, 11.79) | 0.66 (0.53, 0.84) | 0.66 (0.03, 12.27) | 0.86 (0.78, 0.94) | 0.75 (0.21, 2.67) |
| **2010** | 0.72 (0.58, 0.89) | 0.75 (0.31, 1.90) | 0.72 (0.58, 0.89) | 0.75 (0.32, 1.85) | 0.72 (0.58, 0.89) | 0.75 (0.31, 1.90) | 0.78 (0.68, 0.89) | 0.79 (0.53, 1.18) |
| **2012** | 0.74 (0.65, 0.85) | 0.78 (0.46, 1.36) | 0.74 (0.65, 0.85) | 0.78 (0.40, 1.57) | 0.74 (0.64, 0.84) | 0.75 (0.46, 1.22) | 0.75 (0.66, 0.85) | 0.82 (0.54, 1.23) |
| **2014** | 0.71 (0.64, 0.78) | 0.73 (0.61, 0.95) | 0.70 (0.63, 0.78) | 0.73 (0.49, 1.20) | 0.71 (0.64, 0.78) | 0.72 (0.60, 0.91) | 0.73 (0.64, 0.83) | 0.75 (0.69, 0.97) |
| **2016** | 0.69 (0.63, 0.76) | 0.70 (0.61, 0.82) | 0.69 (0.63, 0.75) | 0.70 (0.57, 0.92) | 0.69 (0.63, 0.75) | 0.70 (0.61, 0.81) | 0.79 (0.75, 0.83) | 0.81 (0.77, 0.84) |
| **TCH_52wk_ vs. AC-T** | | | | | | | | |
| **2008** | NA | NA | NA | NA | NA | NA | NA | NA |
| **2010** | NA | NA | NA | NA | NA | NA | NA | NA |
| **2012** | 0.77 (0.27, 2.23) | 0.79 (0.20, 3.26) | 0.77 (0.27, 2.19) | 0.79 (0.17, 3.92) | 1.20 (0.56, 2.61) | 1.22 (0.46, 3.32) | 0.81 (0.38, 1.73) | 0.83 (0.56, 1.22) |
| **2014** | 0.77 (0.27, 2.20) | 0.77 (0.24, 2.34) | 0.77 (0.27, 2.18) | 0.76 (0.20, 2.78) | 1.18 (0.54, 2.53) | 1.17 (0.53, 2.78) | 0.81 (0.42, 1.50) | 0.81 (0.58, 1.13) |
| **2016** | 0.76 (0.63, 0.93) | 0.78 (0.58, 1.06) | 0.76 (0.63, 0.93) | 0.78 (0.51, 1.27) | 0.78 (0.64, 0.95) | 0.81 (0.60, 1.14) | 0.74 (0.63, 0.88) | 0.74 (0.50, 1.09) |
| **AC-TH_52wk_ vs. TCH_52wk_** | | | | | | | | |
| **2008** | NA | NA | NA | NA | NA | NA | NA | NA |
| **2010** | NA | NA | NA | NA | NA | NA | NA | NA |
| **2012** | 0.96 (0.33, 2.78) | 0.99 (0.23, 4.17) | 0.96 (0.34, 2.78) | 1.01 (0.19, 5.26) | 0.62 (0.28, 1.33) | 0.62 (0.23, 1.64) | 0.96 (0.89, 1.04) | 0.98 (0.90, 1.06) |
| **2014** | 0.92 (0.32, 2.63) | 0.95 (0.31, 3.03) | 0.91 (0.32, 2.63) | 0.96 (0.26, 3.85) | 0.60 (0.28, 1.30) | 0.61 (0.26, 1.35) | 0.97 (0.89, 1.04) | 0.93 (0.83, 1.08) |
| **2016** | 0.91 (0.73, 1.12) | 0.90 (0.66, 1.23) | 0.90 (0.72, 1.12) | 0.89 (0.56, 1.43) | 0.88 (0.71, 1.09) | 0.85 (0.62, 1.12) | 0.94 (0.80, 1.13) | 0.91 (0.77, 1.09) |

Hazard ratios with 95% credible intervals are shown. SA#3 could not be performed due to the structure of the evidence networks.

*AC* anthracycline (doxorubicin, epirubicin) + cyclophosphamide, *FE* fixed effects, *H* Herceptin® intravenous (IV), *HER2+* human epidermal growth factor receptor 2-positive, *HR* hormone receptor, *NA* not available, *NMA* network meta-analysis, *NRS* non-randomized studies, *RCT* randomized controlled trial, *RE* random effects, *SA* sensitivity analysis, *T* taxane (docetaxel, paclitaxel), *TCH* docetaxel + carboplatin + Herceptin® IV, *wk* weeks.

***Sensitivity Analysis #1 – RCTs with 100% HER2+ patients***

Evidence networks for sensitivity analysis #1 (RCTs with 100% HER2+ patients) are shown in Figure 1 (interactive figure online: <https://goo.gl/ppkLrG>). The final evidence network in 2016 includes 11 nodes connected by 12 RCTs (11 publications). Data from head-to-head trials were available for 16 pairwise comparisons in the network with single studies informing 11 of the comparisons.

The cNMA results of sensitivity analysis #1 for each of the three pairwise comparisons of interest are presented in Figure 2. Findings from both the FE and RE models are presented. Probability better values from the RE model are shown, which represent the probability of the first treatment being better than the second treatment within each pairwise comparison of interest.

For the pairwise comparison AC-TH_52wk_ vs. AC-T (Figure 2A), initial publicly available evidence in 2008 demonstrated an OS advantage for H/chemotherapy compared with chemotherapy alone (HR 0.67, 95% CrI 0.04 to 11.79). The corresponding probability of AC-TH_52wk_ being better than AC-T at this time point was 79% (SD 41%). The certainty of this survival benefit strengthened over time, resulting in a difference for AC-TH_52wk_ relative to AC-T in 2016 (HR 0.70, 95% CrI 0.57 to 0.92), and a p(better) value of 99% (SD 10%).

For the pairwise comparison TCH_52wk_ vs. AC-T (Figure 2B), no evidence was available for the years 2008 or 2010. Initial evidence in 2012 demonstrated no significant difference in OS with the addition of H compared with chemotherapy alone (HR 0.79, 95% CrI 0.17 to 3.92). Over time, the precision around the OS estimate improved, showing an OS advantage in 2016 (HR 0.78, 95% CrI 0.51 to 1.27). The probability of TCH_52wk_ being better than AC-T in 2012 was 64% (SD 48%), and this increased to 89% (SD 31%) in 2016.

For the pairwise comparison AC-TH_52wk_ vs. TCH_52wk_ (Figure 2C), both H/chemotherapy regimens showed a similar OS advantage. The precision around the effect estimates for this comparison has improved over time. The effect estimate in 2016 showed a slight advantage for AC-TH_52wk_ compared with TCH_52wk_, (HR 0.89, 95% CrI 0.56 to 1.43). The corresponding probability of AC-TH_52wk_ being better than TCH_52wk_in 2016 was 73% (SD 44%).

A visualization of the sample size and the number of events (ie, deaths) included in each network is shown in Figure 2D. A total of 6,752 patients (303 deaths) were included in the 2008 evidence network. By 2016, a total of 26,919 patients were included (3,091 deaths).

Fig. 1 Cumulative NMA evidence networks for overall survival – Sensitivity analysis #1


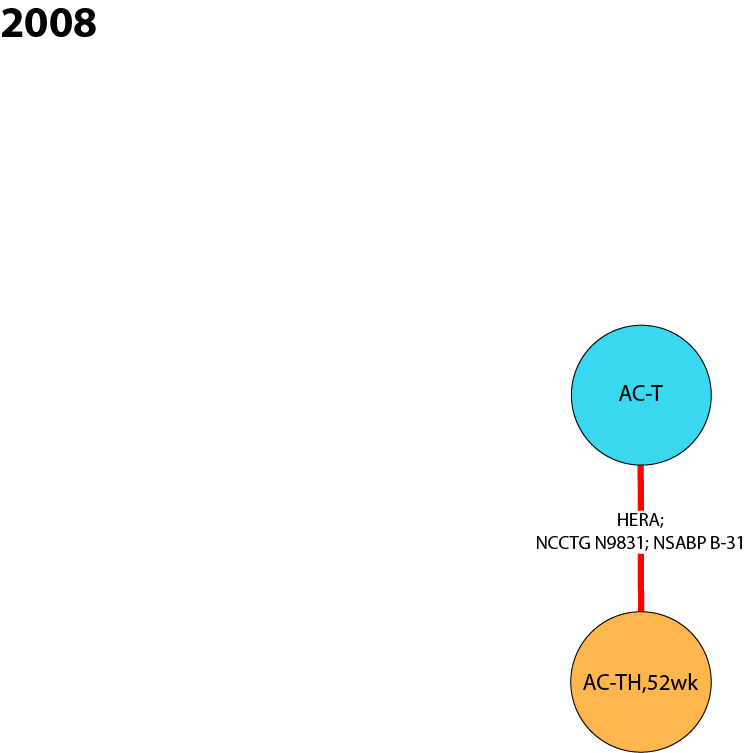


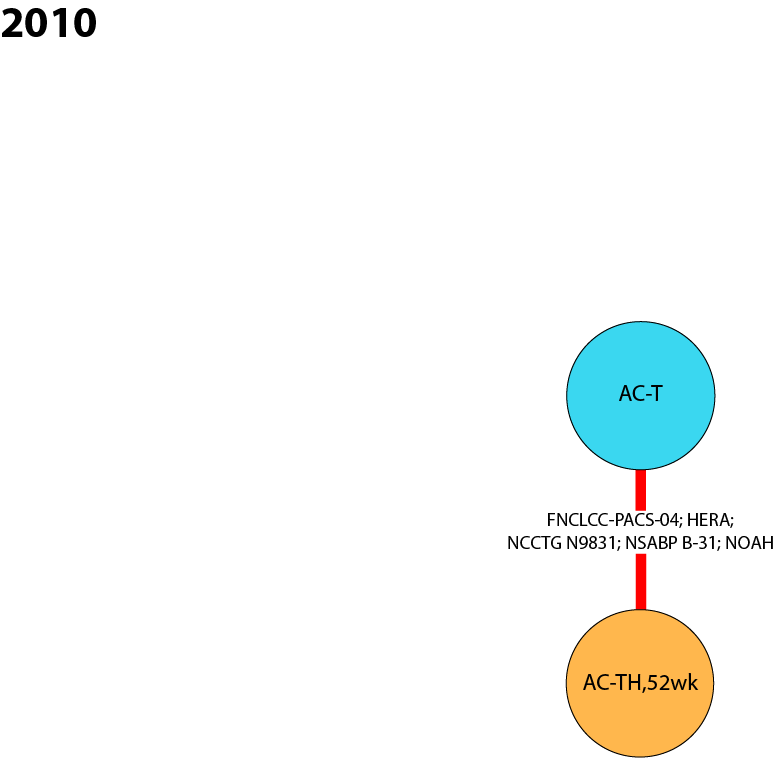


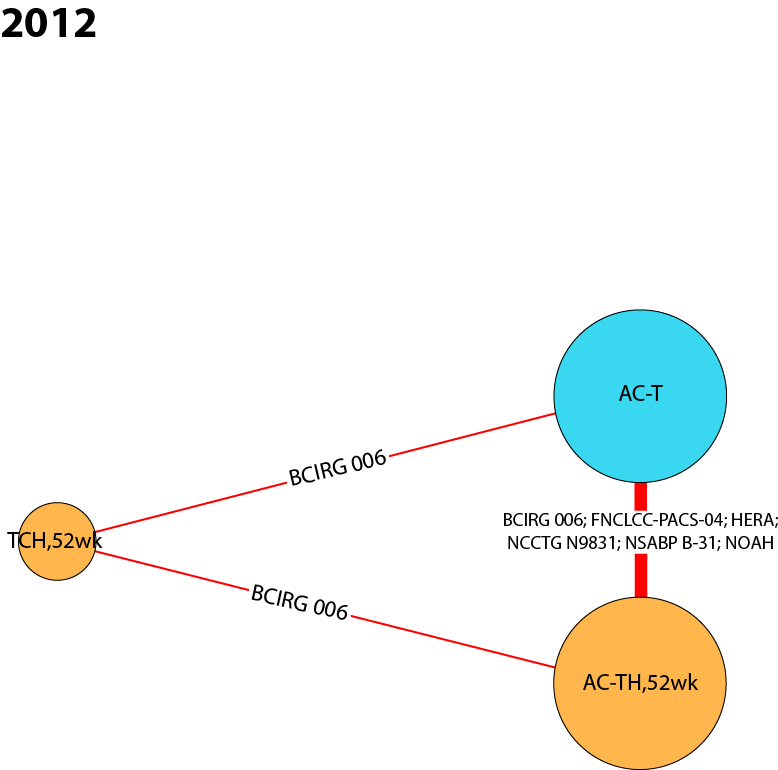


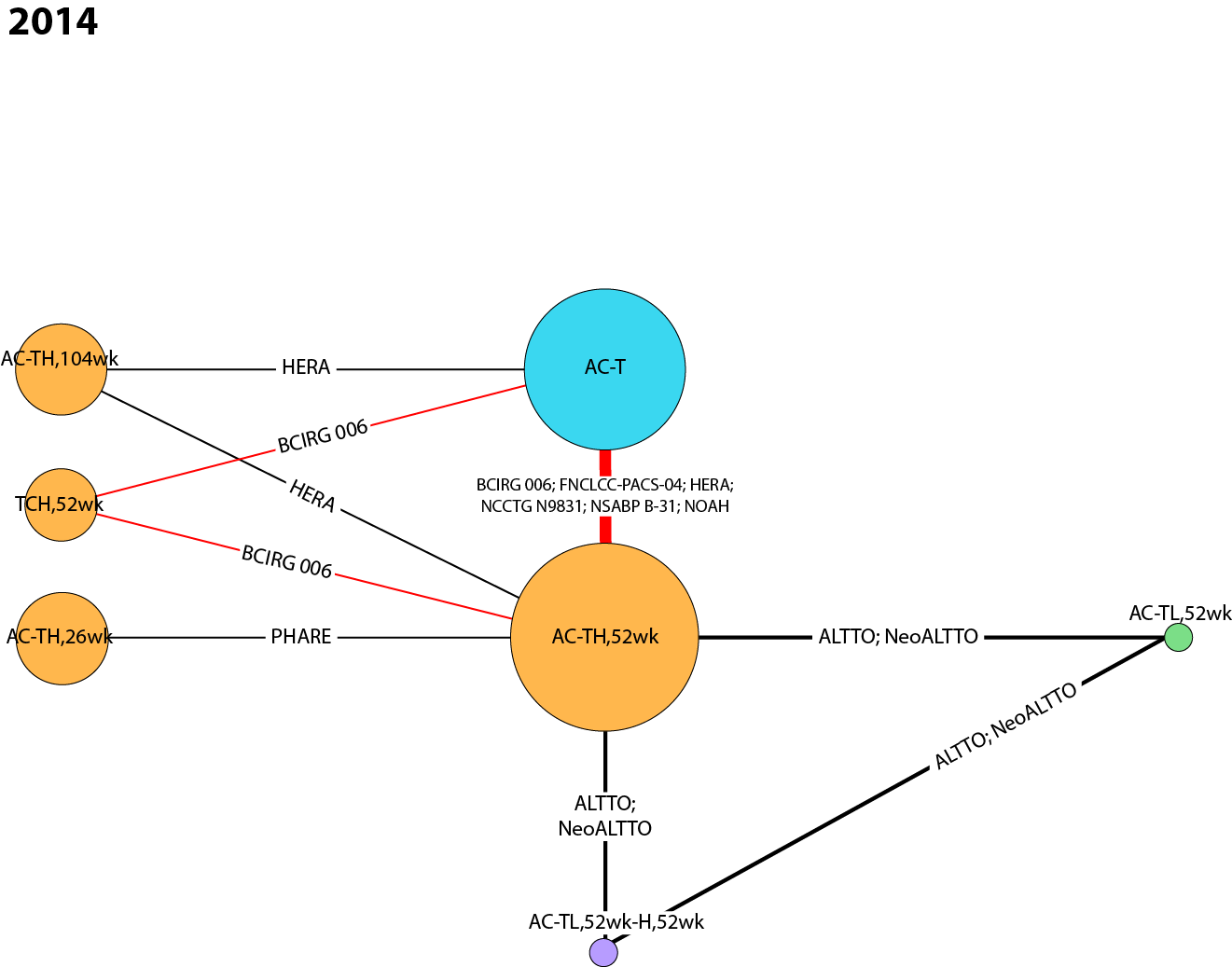


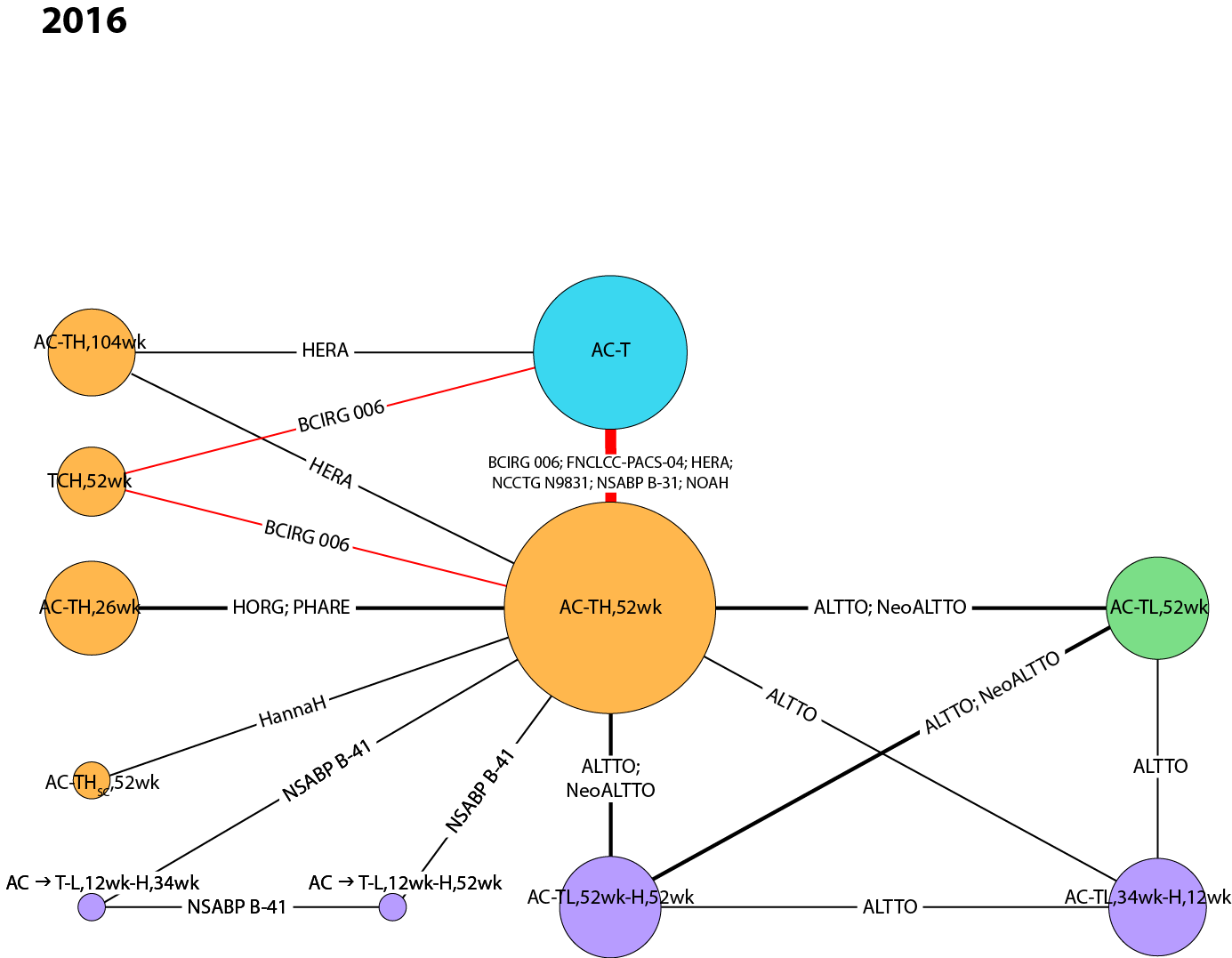


*AC* anthracycline (doxorubicin, epirubicin) + cyclophosphamide, *H* Herceptin® intravenous (IV), *HSC* Herceptin® subcutaneous (SC), *L* lapatinib, *T* taxane (docetaxel, paclitaxel), *TCH* docetaxel + carboplatin + Herceptin® IV, *wk* weeks.

Fig. 2 Cumulative NMA of overall survival results of sensitivity analysis #1 for pairwise comparisons of interest, (A) AC-TH_52wks_ vs. AC-T, (B) TCH_52wks_ vs. AC-T, (C) AC-TH_52wks_ vs. TCH_52wks_, and (D) corresponding sample sizes for each analysis.


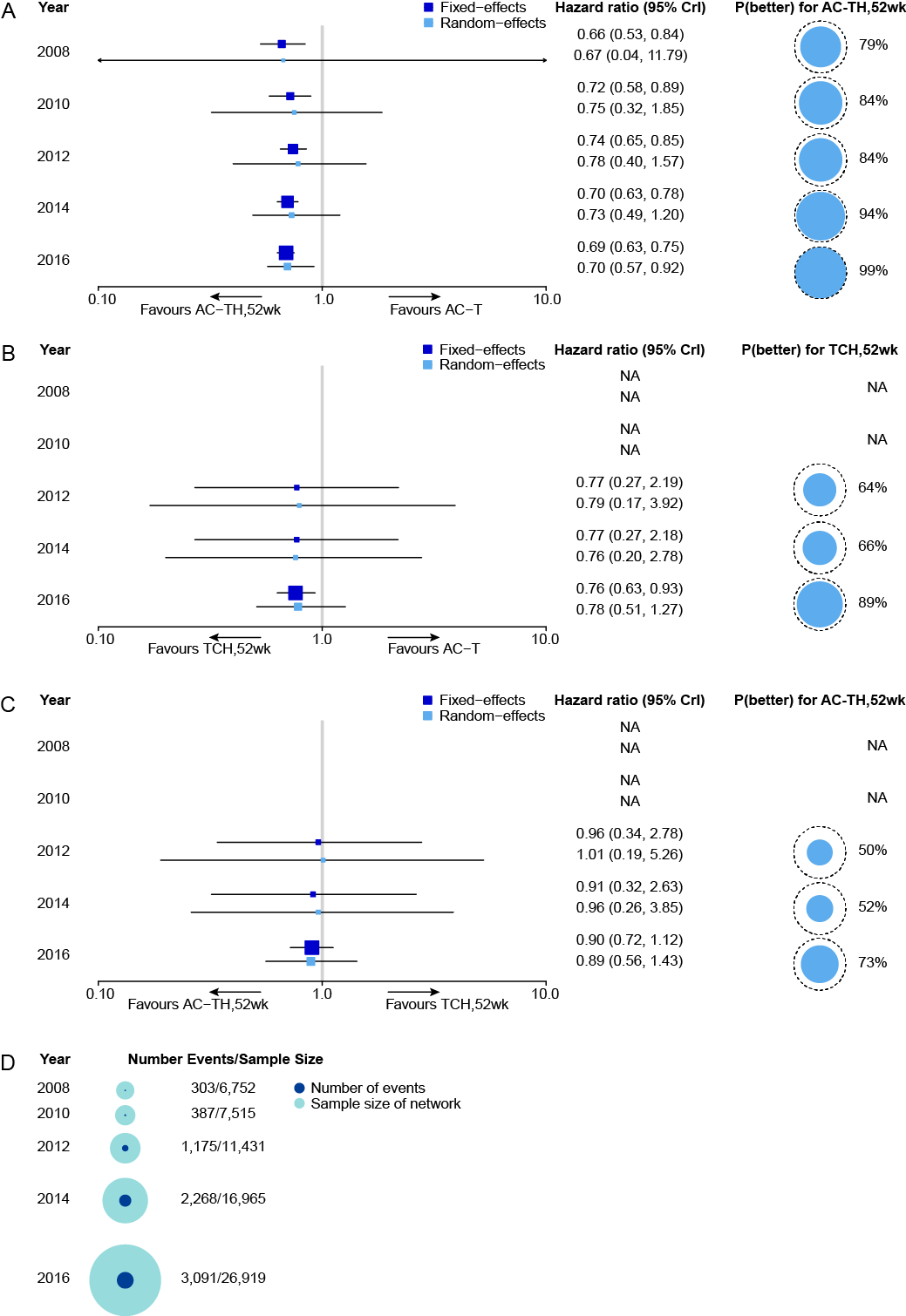


Boxes on the forest plots represent the hazard ratios, with 95% CrIs shown by the horizontal lines. The size of each box is based on the precision of each effect estimate. The x-axis is presented in log-format. Probability better values are based on the random effects model. The dashed circle represents the maximum p(better) value that is possible: 100%. *AC* anthracycline (doxorubicin, epirubicin) + cyclophosphamide, *CrI* credible interval, *H* Herceptin® intravenous (IV), *NA* not available, *OS* overall survival, *P(better)* probability better, *T* taxane (docetaxel, paclitaxel), *TCH* docetaxel + carboplatin + Herceptin® IV, *wk* weeks.

***Sensitivity Analysis #2 – RCTs with 100% HER2+ patients, RCTs with HER2+ subgroups, and non-randomized studies (naïve pooling)***

Evidence networks for sensitivity analysis #2 (RCTs with 100% HER2+ patients, RCTs with HER2+ subgroups, and non-randomized studies) are shown in Figure 3 (interactive figure online: <https://goo.gl/ppkLrG>). The final evidence network in 2016 includes 22 nodes connected by 31 studies (29 publications). Data from head-to-head trials were available for 33 pairwise comparisons in the network with single studies informing 24 of the comparisons.

The cNMA results of sensitivity analysis #2 for each of the three pairwise comparisons of interest are presented in Figure 4. Findings from both the FE and RE models are presented, with p(better) values from the RE model.

For the pairwise comparison AC-TH_52wk_ vs. AC-T (Figure 4A), initial evidence in 2008 demonstrated an OS advantage for H/chemotherapy versus chemotherapy alone (HR 0.66, 95% CrI 0.03 to 12.27). The corresponding probability of AC-TH_52wk_ being better than AC-T at this time point was 79% (SD 41%). The certainty of this survival benefit strengthened over time, resulting in a difference for AC-TH_52wk_ relative to AC-T in 2016 (HR 0.69, 95% CrI 0.61 to 0.81), and a p(better) value of 100% (SD 2%).

For the pairwise comparison TCH_52wk_ vs. AC-T (Figure 4B), no evidence was available for the years 2008 or 2010. Initial evidence in 2012 demonstrated no significant difference in OS for H/chemotherapy versus chemotherapy alone (HR 1.23, 95% CrI 0.45 to 3.47). Over time, there was a shift to an OS advantage for H/chemotherapy versus chemotherapy alone, and the precision around the OS estimate improved (2016 HR 0.80, 95% CrI 0.62 to 1.12. The probability of TCH_52wk_ being better than AC-T in 2012 was 33% (SD 47%), but this increased to 92% (SD 27%) in 2016.

For the pairwise comparison AC-TH_52wk_ vs. TCH_52wk_ (Figure 4C), both H/chemotherapy regimens showed an OS advantage. Initial evidence in 2012 suggested an OS advantage for AC-TH_52wk_­ compared with TCH_52wk_, although not significant (HR 0.61, 95% CrI 0.22 to 1.61). The precision around the effect estimates for this comparison improved over time, with a shift towards both H/chemotherapy regimens showing a similar OS advantage in 2016 (HR 0.86, 95% CrI 0.62 to 1.14). The probability of AC-TH_52wk_ being better than TCH_52wk_ remained relatively constant from 2012 to 2016, changing from 85% (SD 35%) to 87% (SD 34%), respectively.

A visualization of the sample size and the number of events in each network is shown in Figure 4D. A total of 7,341 patients (380 deaths) were included in the 2008 evidence network. By 2016, a total of 34,394 patients (4,059 deaths) were included.

Fig. 3 Cumulative NMA evidence networks for overall survival – Sensitivity analysis #2


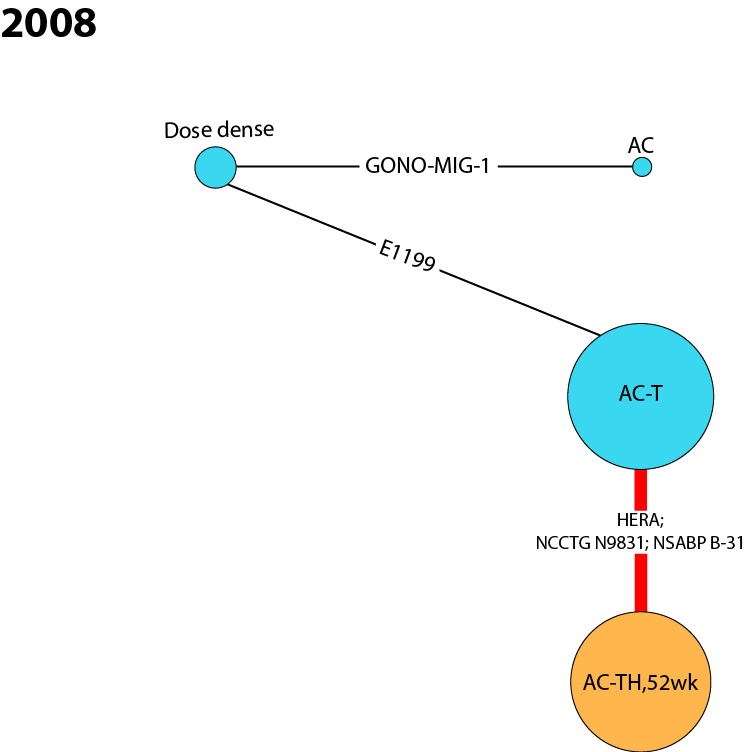


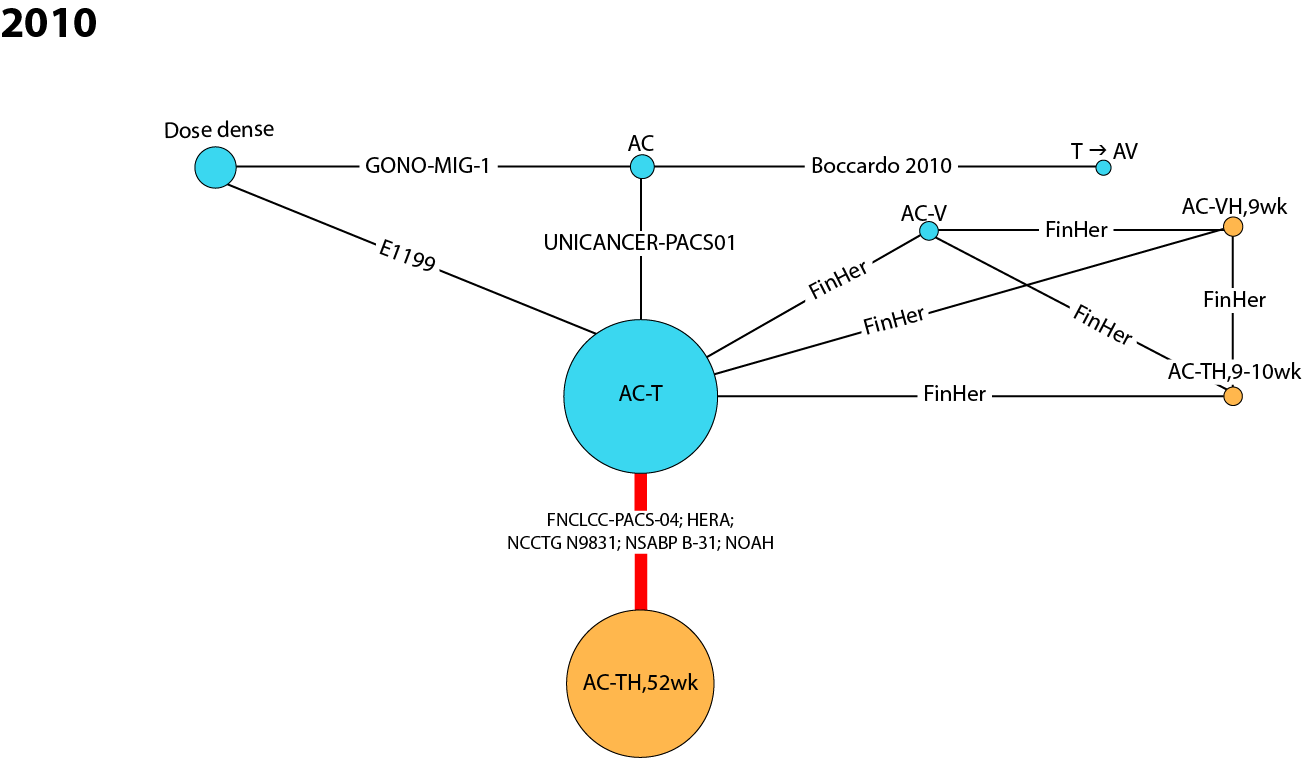


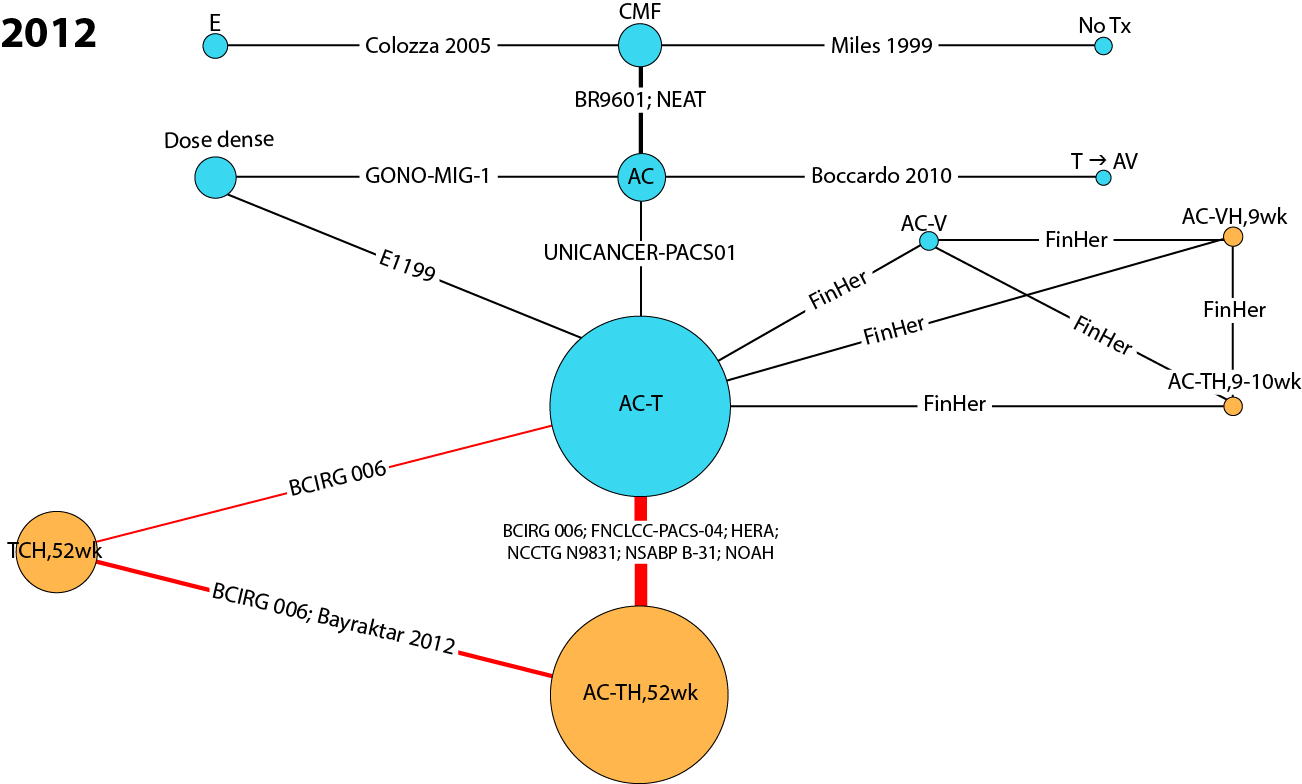


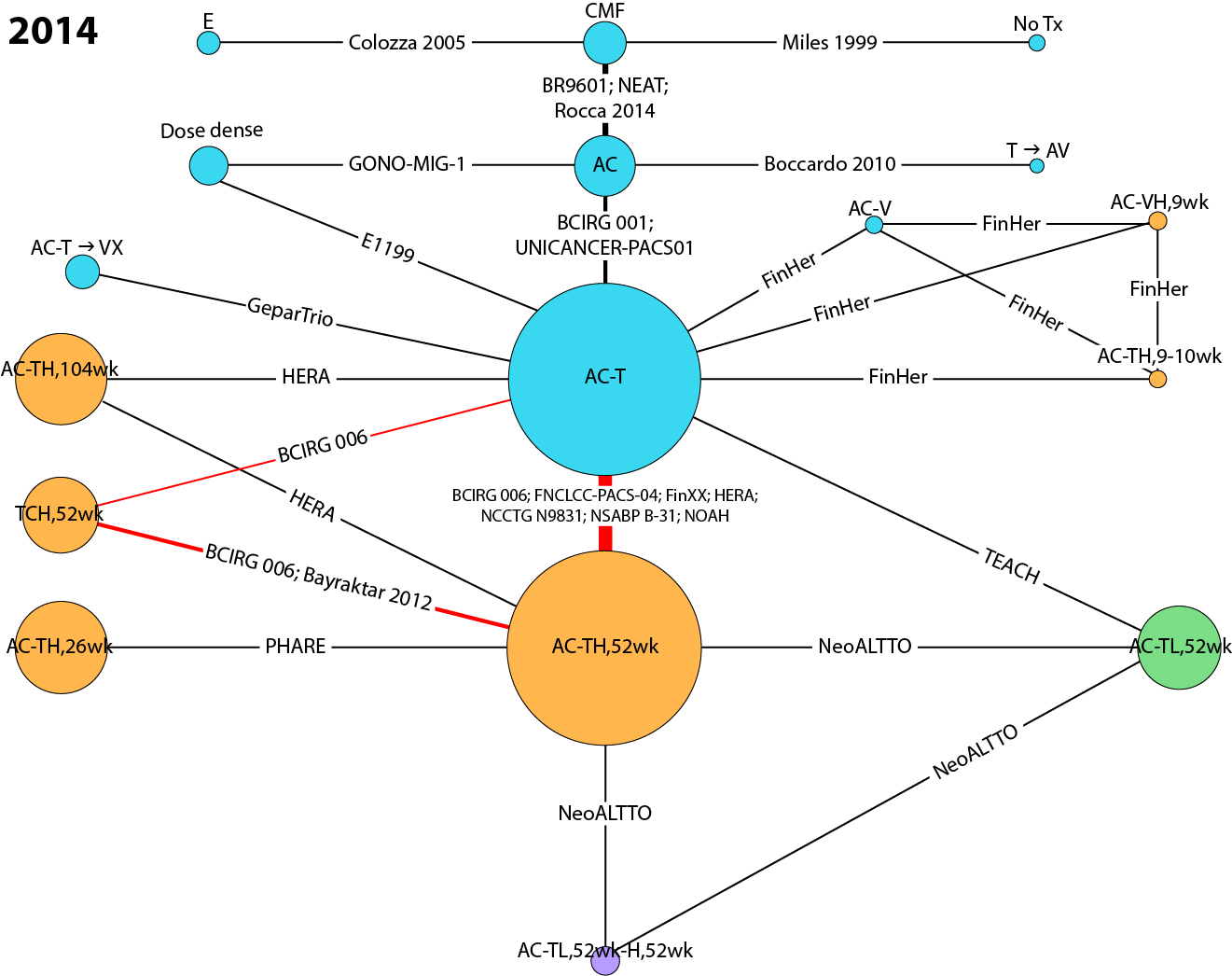


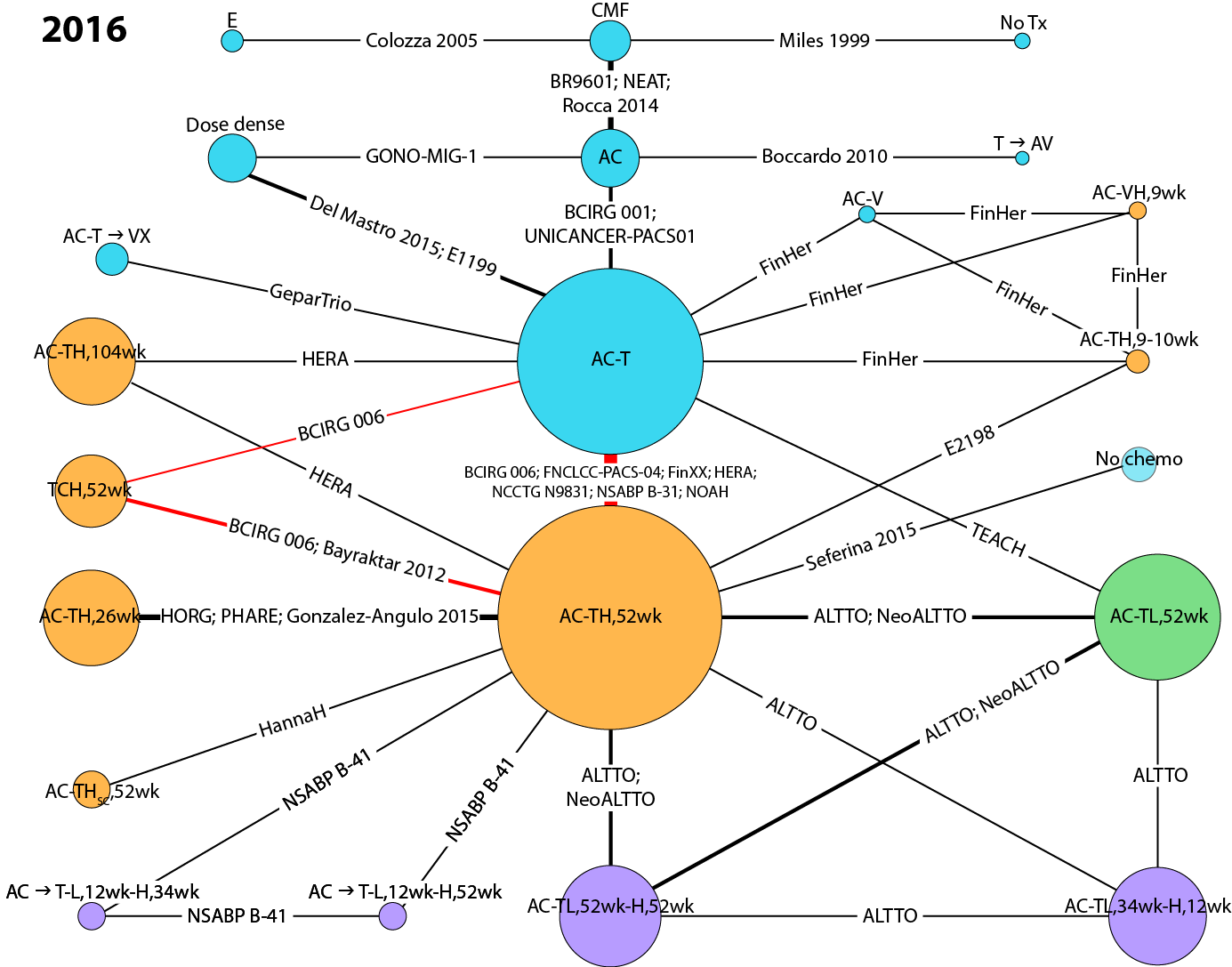


*AC* anthracycline (doxorubicin, epirubicin) + cyclophosphamide, *Dose dense* AC → T, or AC, either weekly or biweekly, *E* epirubicin, *H* Herceptin® intravenous (IV), *HSC* Herceptin® subcutaneous (SC), *L* lapatinib, *No chemo* no chemotherapy (includes endocrine therapy and radiotherapy), *No Tx* no treatment, *T* taxane (docetaxel, paclitaxel), *TCH* docetaxel + carboplatin + Herceptin® IV, *V* vinorelbine, *wk* weeks, *X* capecitabine.

Fig. 4 Cumulative NMA of overall survival results of sensitivity analysis #2 for pairwise comparisons of interest, (A) AC-TH_52wks_ vs. AC-T, (B) TCH_52wks_ vs. AC-T, (C) AC-TH_52wks_ vs. TCH_52wks_, and (D) corresponding sample sizes for each analysis.


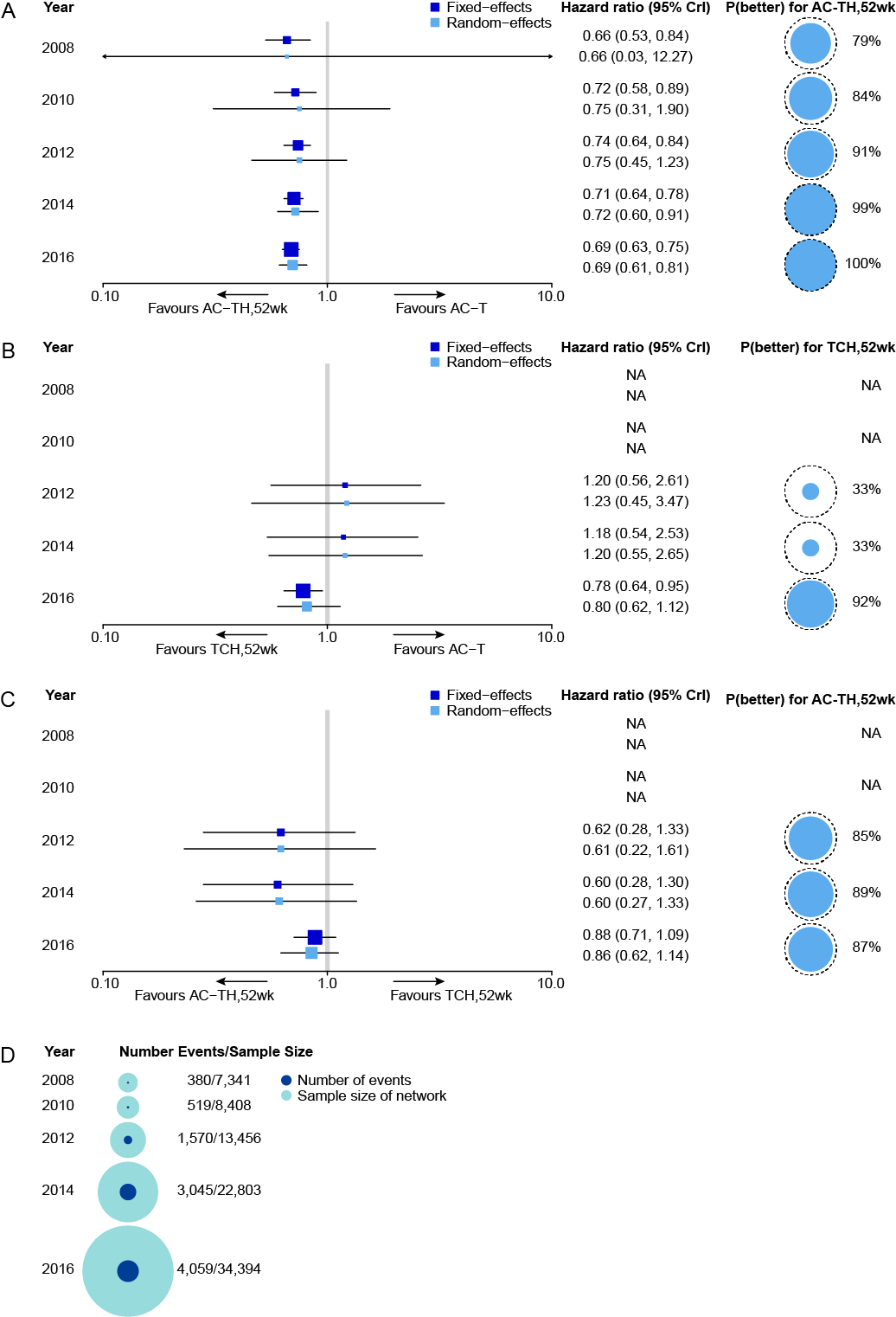


Boxes on the forest plots represent the hazard ratios, with 95% CrIs shown by the horizontal lines. The size of each box is based on the precision of each effect estimate. The x-axis is presented in log-format. Probability better values are based on the random effects model. The dashed circle represents the maximum p(better) value that is possible: 100%. *AC* anthracycline (doxorubicin, epirubicin) + cyclophosphamide, *CrI* credible interval, *H* Herceptin® intravenous (IV), *NA* not available, *OS* overall survival, *P(better)* probability better, *T* taxane (docetaxel, paclitaxel), *TCH* docetaxel + carboplatin + Herceptin® IV, *wk* weeks.

***Sensitivity Analysis #4 – RCTs with 100% HER2+ patients and RCTs with HER2+ subgroups, using whole survival curves***

Evidence networks for sensitivity analysis #4 include RCTs with 100% HER2+ patients and RCTs with HER2+ subgroups where data is available from whole survival curves (Figure 5; interactive figure online: <https://goo.gl/ppkLrG>). The final evidence network in 2016 includes 12 nodes connected by 14 studies (13 publications). Data from head-to-head trials were available for 19 pairwise comparisons in the network with single studies informing 14 of the comparisons.

Based on methods outlined by Jansen (2011), a multi-dimensional treatment effect approach was used to model the hazard over time with fractional polynomials.^43^ This method allows an NMA of survival to be performed with models that can more closely fit the data.^43^ When available, the number of patients at risk reported underneath Kaplan–Meier curves in publications was captured. If these data were not available, Kaplan–Meier curves were digitized (TechDig20). A standard censoring function was applied to data sets derived from digitized curves. This aggregate data from survival curves was analyzed with fractional polynomial NMA models using WinBUGS software (MRC Biostatistics Unit, Cambridge, UK).

The cNMA results of sensitivity analysis #4 (reference case using whole survival curves) for each of the three pairwise comparisons of interest are presented in Table 12. Overall, these results align with the reference case. The evidence networks for sensitivity analysis #4 are smaller than those for the reference case, so although there are differences in results, there are no changes in directionality of the point estimates.

Fig. 5 Cumulative NMA evidence networks for overall survival – Sensitivity analysis #4


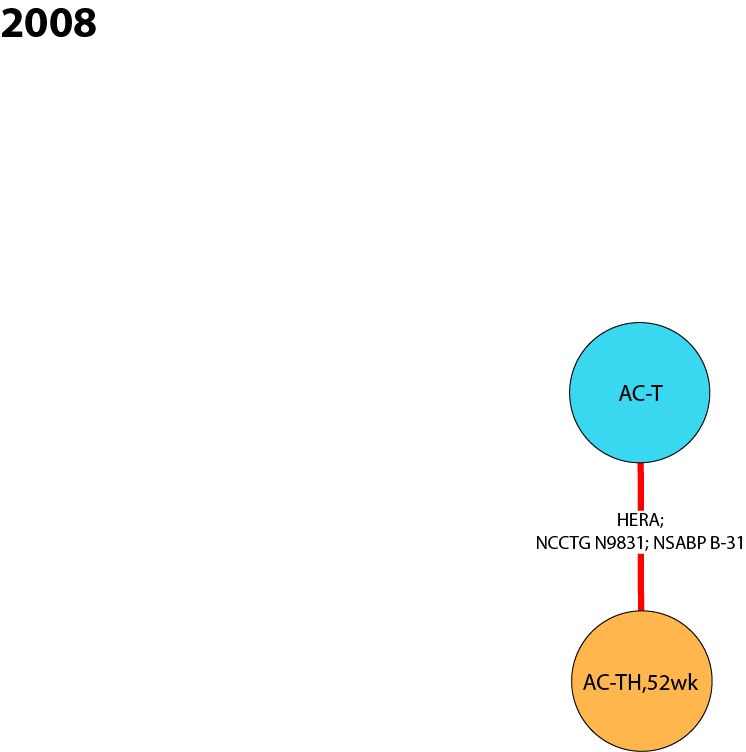


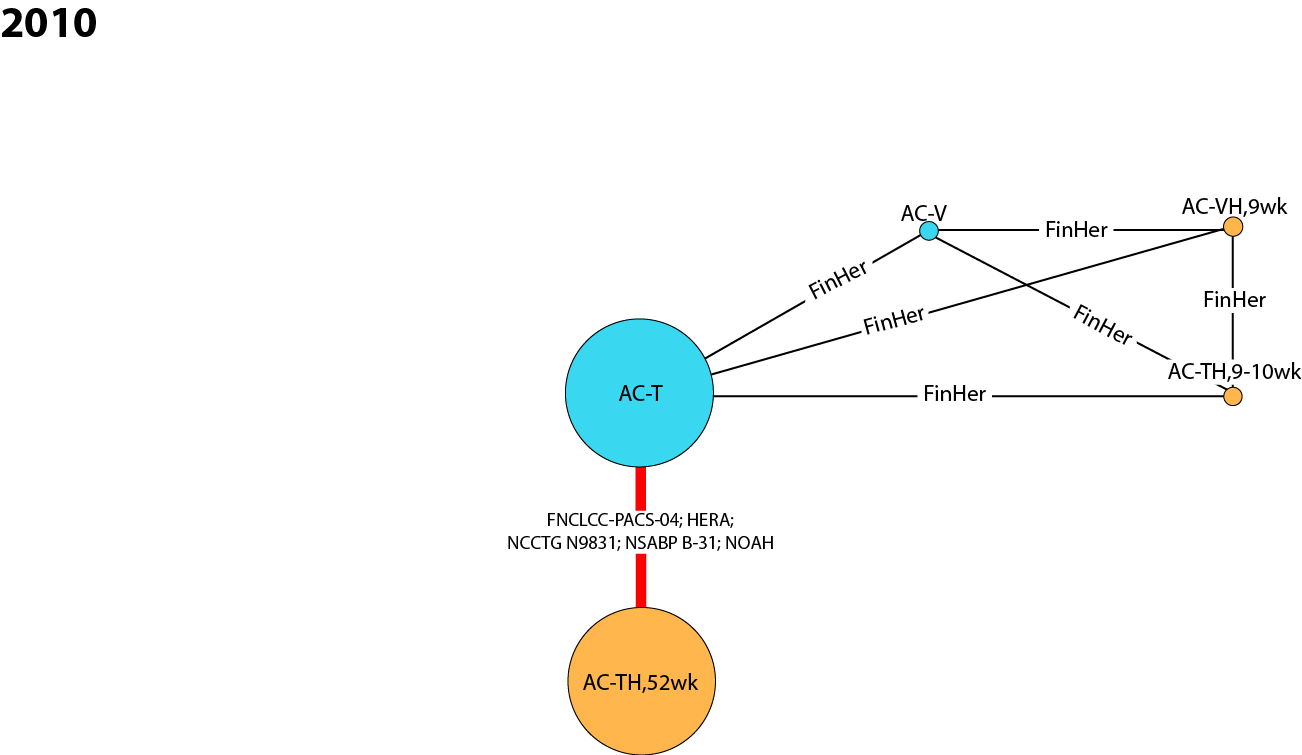


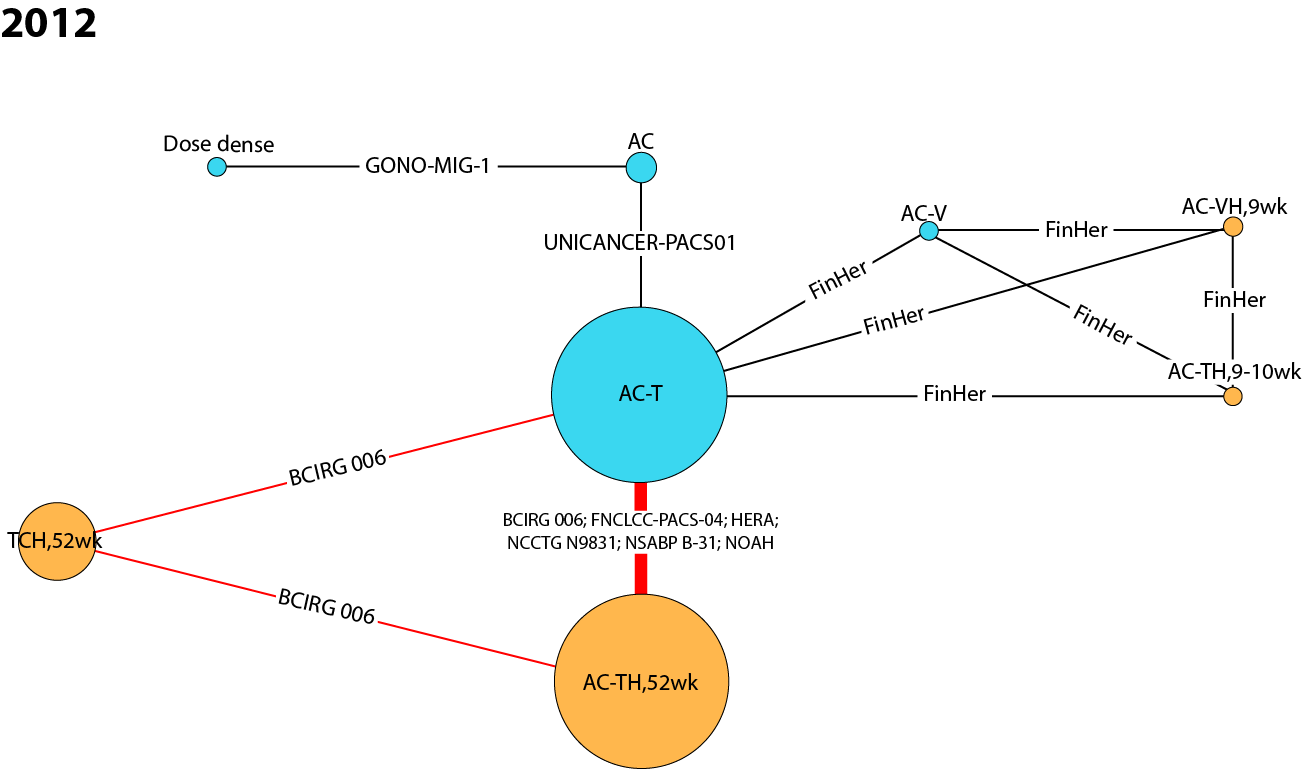


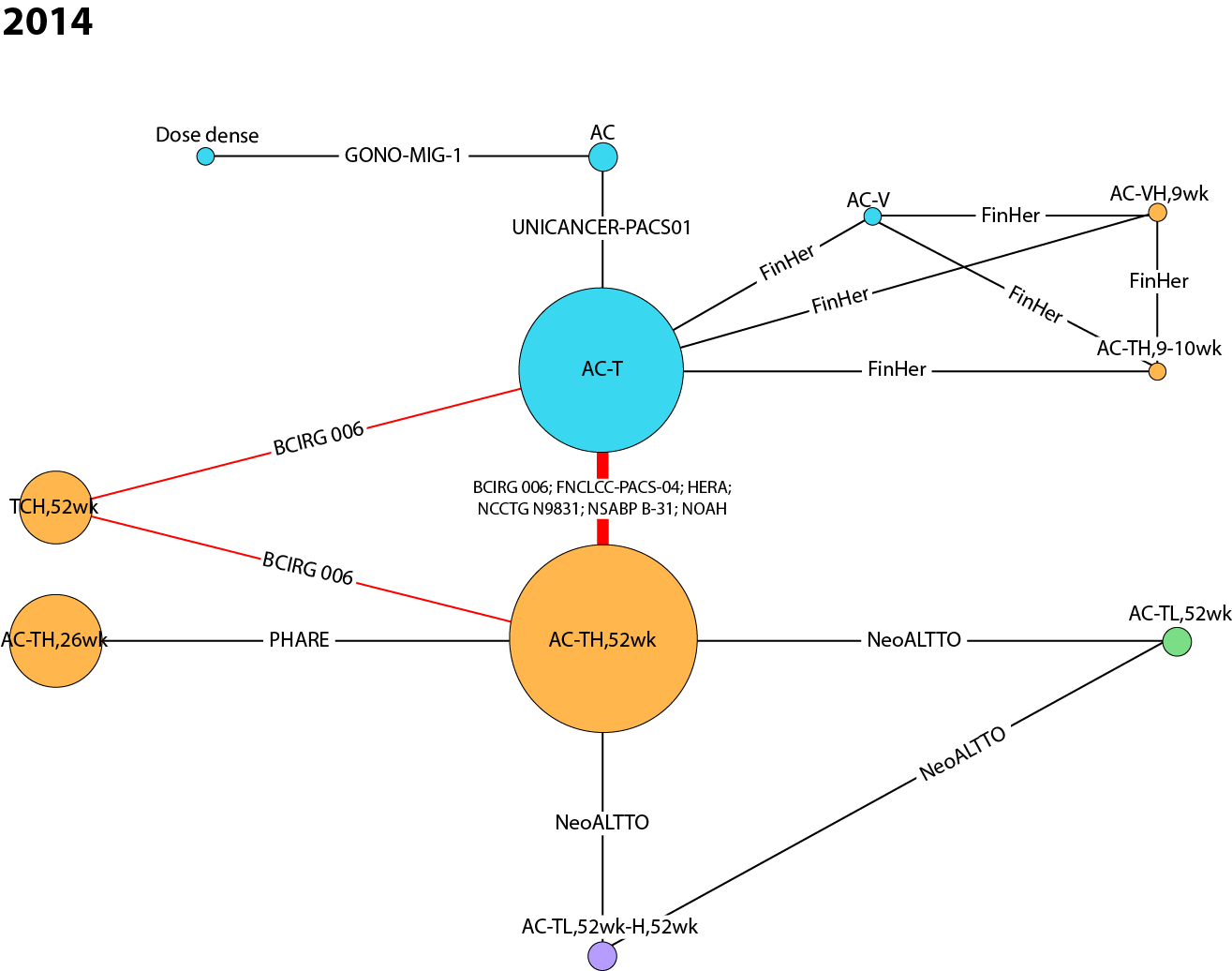


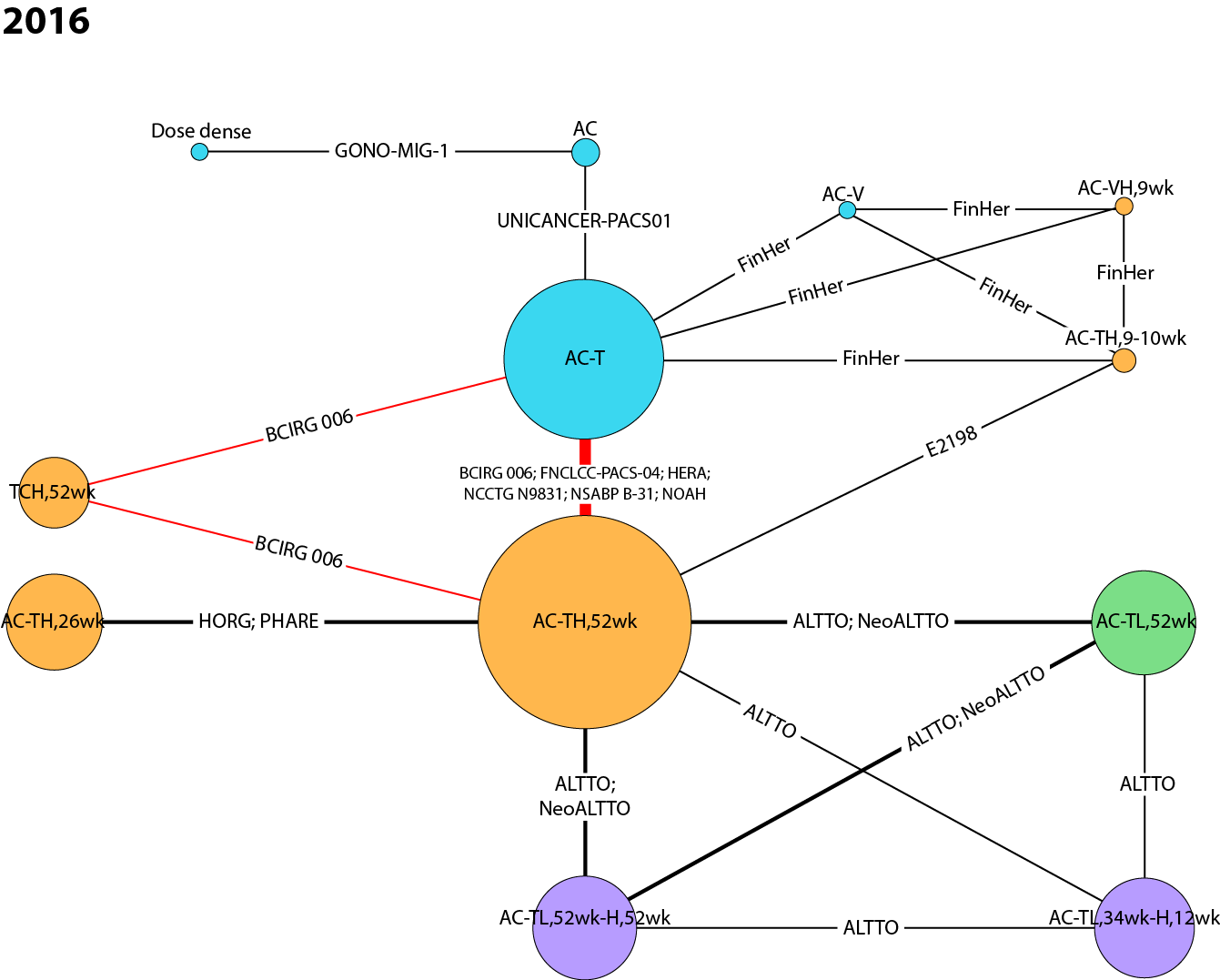


*AC* anthracycline (doxorubicin, epirubicin) + cyclophosphamide, *Dose dense* AC → T, or AC, either weekly or biweekly, *E* epirubicin, *H* Herceptin® intravenous (IV), *L* lapatinib, *T* taxane (docetaxel, paclitaxel), *TCH* docetaxel + carboplatin + Herceptin® IV, *V* vinorelbine, *wk* weeks, *X* capecitabine.

Inconsistency Analyses

The posterior mean deviance of the individual data points in the inconsistency model was plotted against their posterior mean deviance in the consistency model to help identify loops in which inconsistency was present. Fixed effects and random effects consistency and inconsistency models were performed for the OS reference case analysis at the 2016 timepoint. Both consistency models were associated with lower DIC values compared with the corresponding inconsistency models, which suggests that performing an NMA was worthwhile.

A scatterplot of the fixed effects model is shown in Figure 6. The majority of data points were close to 1, but a few points were greater than 1.25 or less than 0.25: FNCLCC-PACS 04 (AC-TH,52wk), HORG (AC-TH,26wk), NeoALTTO (AC-TL,52wk), NCCTG N9831 & NSABP B-31 (AC-TH,52wk), ALTTO (AC-TL, 52wk), NOAH (AC-TH,52wk), and FinXX (AC-TH,52wk). However, all data points are positioned close to the diagonal line, which suggests there is no major inconsistency. The fixed effects consistency model had a slightly lower posterior mean of the residual deviance compared with the fixed effects inconsistency model (33.72 vs. 35.83), and also a smaller DIC (22.39 vs. 27.47). The parameter estimates were similar for both models and there was considerable overlap in the 95% CrIs (Table 13). This suggests no evidence of severe inconsistency in the network, although this should be interpreted with caution as there may not have been sufficient power to detect inconsistency.

Fig. 6 Plot of posterior mean deviance of the individual data points in the inconsistency model against their posterior mean deviance in the consistency model – fixed effects model


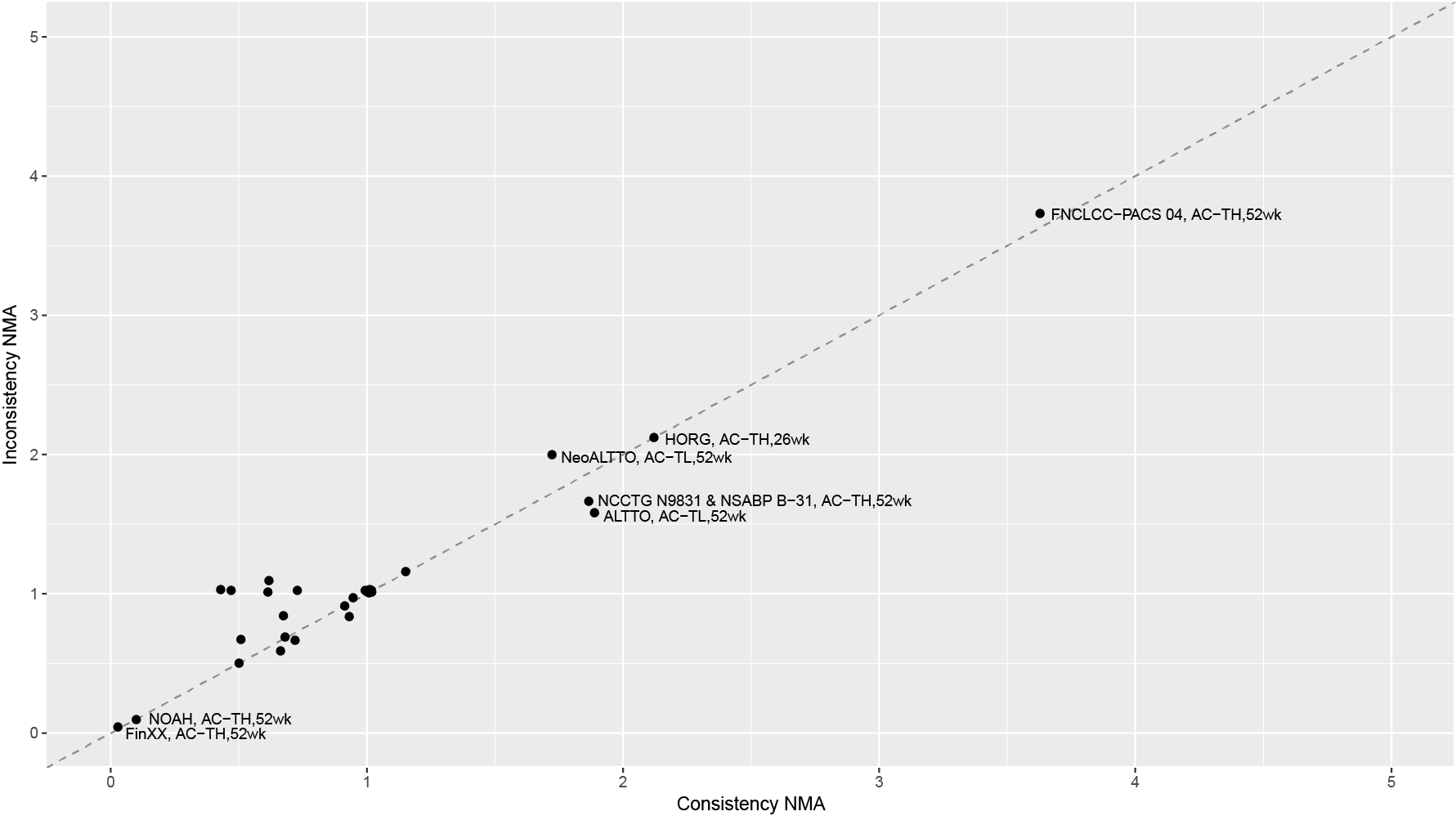


Table 13 Comparison of hazard ratios from consistency and inconsistency models – fixed effects

| Comparison | Fixed effects Consistency NMA  HR (95% CrI) | Fixed effects Inconsistency NMA  HR (95% CrI) |
| --- | --- | --- |
| AC-TH,52wk vs. AC-T | 0.69 (0.63 to 0.76) | 0.69 (0.63 to 0.75) |
| TCH,52wk vs. AC-T | 0.76 (0.63 to 0.93) | 0.76 (0.63 to 0.93) |
| AC-TH,104wk vs. AC-T | 0.71 (0.56 to 0.89) | 0.71 (0.56 to 0.89) |
| AC-TL,52wk vs. AC-T | 0.93 (0.77 to 1.12) | 1.02 (0.75 to 1.39) |
| AC-TH,9-10wk vs. AC-T | 0.54 (0.26 to 1.14) | 0.50 (0.16 to 1.60) |
| AC-T → VX vs. AC-T | 0.72 (0.51 to 1.03) | 0.72 (0.51 to 1.03) |
| AC-VH,9wk vs. AC-T | 0.89 (0.27 to 2.97) | 0.89 (0.27 to 2.94) |
| AC-V vs. AC-T | 1.35 (0.43 to 4.16) | 1.35 (0.43 to 4.21) |
| Dose Dense vs. AC-T | 0.80 (0.58 to 1.10) | 0.77 (0.55 to 1.09) |
| AC vs. AC-T | 1.65 (1.21 to 2.24) | 1.70 (1.23 to 2.36) |
| AC-TH,26wk vs. AC-TH,52wk | 1.35 (1.00 to 1.83) | 1.35 (1.00 to 1.82) |
| AC-THSC,52wk vs. AC-TH,52wk | 0.76 (0.44 to 1.31) | 0.76 (0.44 to 1.32) |
| AC-TL,52wk vs. AC-TH,52wk | 1.34 (1.12 to 1.61) | 1.28 (1.03 to 1.59) |
| AC-TL,52wk-H,52wk vs. AC-TH,52wk | 0.78 (0.61 to 0.99) | 0.78 (0.61 to 0.98) |
| AC-TL,34wk-H,12wk vs. AC-TH,52wk | 0.91 (0.71 to 1.16) | 0.90 (0.71 to 1.16) |
| AC → T-L,12wk-H,52wk vs. AC-TH,52wk | 0.63 (0.24 to 1.67) | 0.63 (0.24 to 1.66) |
| AC → T-L,12wk-H,34wk vs. AC-TH,52wk | 1.52 (0.69 to 3.35) | 1.52 (0.69 to 3.35) |
| AC-TH,9-10wk vs. AC-TH,52wk | 0.78 (0.38 to 1.65) | 0.83 (0.32 to 2.14) |
| AC vs. T → AV | 0.68 (0.32 to 1.44) | 0.68 (0.32 to 1.46) |
| AC vs. Dose Dense | 2.06 (1.36 to 3.12) | 1.69 (0.74 to 3.92) |
| AC vs. CMF | 0.69 (0.47 to 1.01) | 0.69 (0.47 to 1.01) |
| No Tx vs. CMF | 1.61 (0.98 to 2.66) | 1.62 (0.98 to 2.66) |
| E vs. CMF | 1.64 (0.48 to 5.68) | 1.64 (0.47 to 5.77) |
| Model Fit Statistics | **DIC = 22.39**  **TotResDev = 33.72 vs. 34.00** | **DIC = 27.47**  **TotResDev = 35.83 vs. 34.00** |

*AC* anthracycline (doxorubicin, epirubicin) + cyclophosphamide, *AV* anthracycline + vinorelbine, *CrI* credible interval, *CMF* cyclophosphamide + methotrexate + fluorouracil, *DIC* deviance information criterion, *Dose dense*  AC → T, or AC, either weekly or biweekly, *E* epirubicin, *H* Herceptin® intravenous (IV), *HR* hazard ratio, *HSC* Herceptin® subcutaneous (SC), *L* lapatinib, *NMA* network meta-analysis, *No Tx* no treatment, *T* taxane (docetaxel, paclitaxel), *TCH* docetaxel + carboplatin + Herceptin® IV, *TotResDev* total residual deviance, *V* vinorelbine, *wk* weeks, *X* capecitabine.

A scatterplot of the random effects model is shown in Figure 7. The majority of data points were close to 1, but a few points were greater than 1.25 or less than 0.25: FNCLCC-PACS 04 (AC-TH,52wk), HORG (AC-TH,26wk), ALTTO (AC-TL,52wk), NCCTG N9831 & NSABP B-31 (AC-TH,52wk), and FinXX (AC-TH,52wk). However, all data points are positioned close to the diagonal line, which suggests there is no major inconsistency. The random effects consistency model had a slightly lower posterior mean of the residual deviance compared with the random effects inconsistency model (31.88 vs. 33.57), and also a smaller DIC (23.43 vs. 28.17). The parameter estimates were similar for both models and there was considerable overlap in the 95% CrIs (Table 14). This suggests no evidence of severe inconsistency in the network, although this should be interpreted with caution as there may not have been sufficient power to detect inconsistency.

Fig. 7 Plot of posterior mean deviance of the individual data points in the inconsistency model against their posterior mean deviance in the consistency model – random effects model


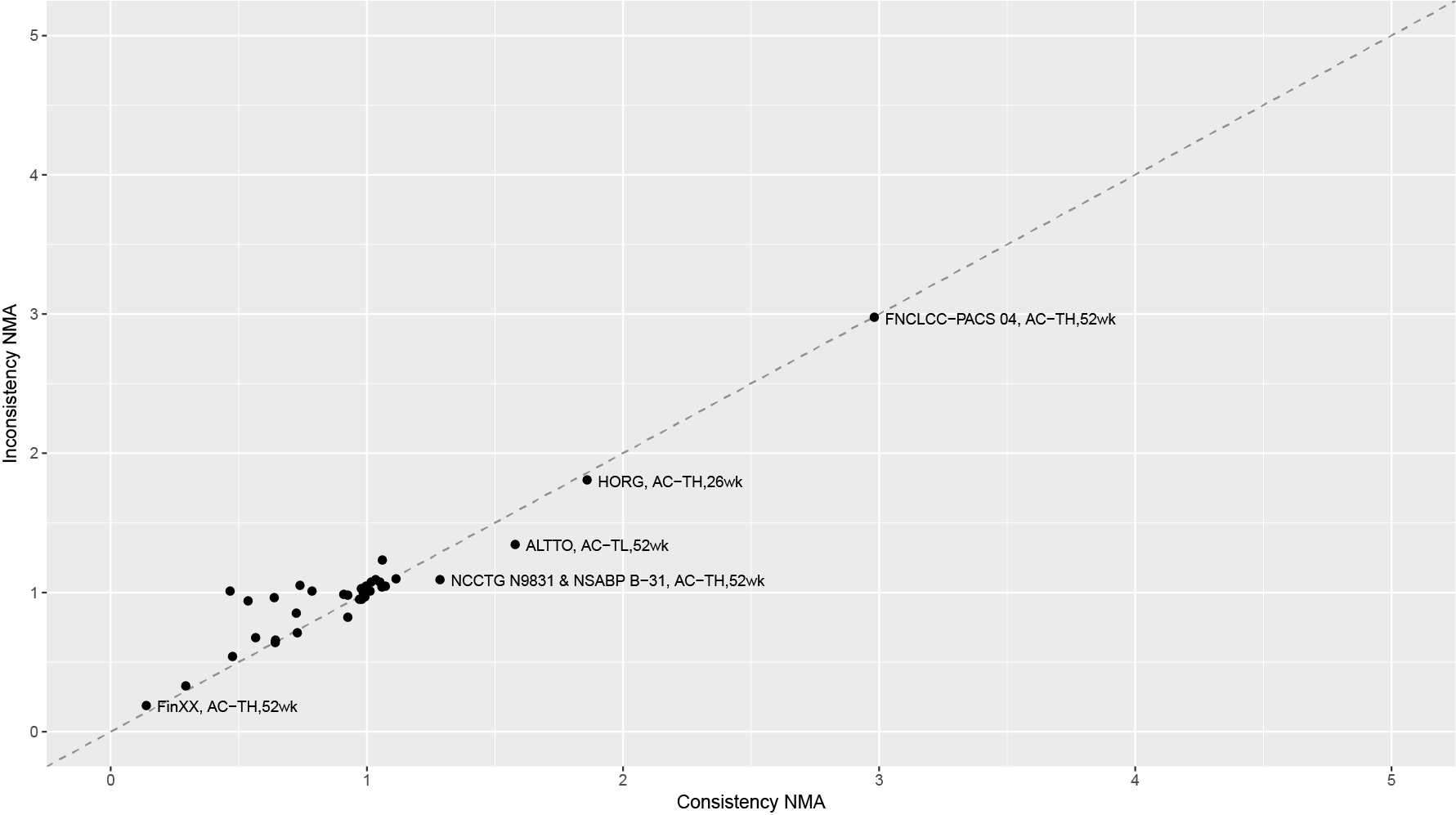


Table 14 Comparison of hazard ratios from consistency and inconsistency models – random effects

| Comparison | Fixed effects Consistency NMA  HR (95% CrI) | Fixed effects Inconsistency NMA  HR (95% CrI) |
| --- | --- | --- |
| AC-TH,52wk vs. AC-T | 0.70 (0.61 to 0.82) | 0.69 (0.59 to 0.84) |
| TCH,52wk vs. AC-T | 0.77 (0.59 to 1.07) | 0.76 (0.53 to 1.09) |
| AC-TH,104wk vs. AC-T | 0.70 (0.51 to 0.96) | 0.71 (0.49 to 1.05) |
| AC-TL,52wk vs. AC-T | 0.93 (0.72 to 1.19) | 1.02 (0.67 to 1.56) |
| AC-TH,9-10wk vs. AC-T | 0.56 (0.25 to 1.14) | 0.49 (0.15 to 1.71) |
| AC-T → VX vs. AC-T | 0.72 (0.47 to 1.10) | 0.72 (0.46 to 1.14) |
| AC-VH,9wk vs. AC-T | 0.97 (0.27 to 2.82) | 0.92 (0.27 to 3.01) |
| AC-V vs. AC-T | 1.28 (0.42 to 4.18) | 1.32 (0.42 to 4.54) |
| Dose Dense vs. AC-T | 0.80 (0.56 to 1.15) | 0.78 (0.52 to 1.14) |
| AC vs. AC-T | 1.67 (1.18 to 2.33) | 1.70 (1.17 to 2.52) |
| AC-TH,26wk vs. AC-TH,52wk | 1.33 (0.89 to 1.88) | 1.31 (0.86 to 1.89) |
| AC-THSC,52wk vs. AC-TH,52wk | 0.76 (0.42 to 1.37) | 0.76 (0.41 to 1.39) |
| AC-TL,52wk vs. AC-TH,52wk | 1.32 (1.02 to 1.67) | 1.26 (0.87 to 1.68) |
| AC-TL,52wk-H,52wk vs. AC-TH,52wk | 0.77 (0.56 to 1.03) | 0.77 (0.54 to 1.06) |
| AC-TL,34wk-H,12wk vs. AC-TH,52wk | 0.90 (0.64 to 1.24) | 0.90 (0.62 to 1.33) |
| AC → T-L,12wk-H,52wk vs. AC-TH,52wk | 0.62 (0.24 to 1.77) | 0.61 (0.22 to 1.67) |
| AC → T-L,12wk-H,34wk vs. AC-TH,52wk | 1.51 (0.68 to 3.38) | 1.49 (0.66 to 3.66) |
| AC-TH,9-10wk vs. AC-TH,52wk | 0.79 (0.35 to 1.61) | 0.80 (0.32 to 2.21) |
| AC vs. T → AV | 0.68 (0.31 to 1.52) | 0.70 (0.31 to 1.55) |
| AC vs. Dose Dense | 2.08 (1.33 to 3.25) | 1.68 (0.71 to 4.06) |
| AC vs. CMF | 0.67 (0.45 to 1.03) | 0.67 (0.44 to 1.06) |
| No Tx vs. CMF | 1.59 (0.94 to 2.76) | 1.63 (0.92 to 2.83) |
| E vs. CMF | 1.69 (0.47 to 6.05) | 1.60 (0.42 to 6.17) |
| Model Fit Statistics | **DIC = 23.43**  **TotResDev = 31.88 vs. 34.00** | **DIC = 28.17**  **TotResDev = 33.57 vs. 34.00** |

*AC* anthracycline (doxorubicin, epirubicin) + cyclophosphamide, *AV* anthracycline + vinorelbine, *CrI* credible interval, *CMF* cyclophosphamide + methotrexate + fluorouracil, *DIC* deviance information criterion, *Dose dense* AC → T, or AC, either weekly or biweekly, *E* epirubicin, *H* Herceptin® intravenous (IV), *HR* hazard ratio, *HSC* Herceptin® subcutaneous (SC), *L* lapatinib, *NMA* network meta-analysis, *No Tx* no treatment, *T* taxane (docetaxel, paclitaxel), *TCH* docetaxel + carboplatin + Herceptin® IV, *TotResDev* total residual deviance, *V* vinorelbine, *wk* weeks, *X* capecitabine.

Subgroup Analyses

The following predefined group-level factors were considered for subgroup analyses:

1. Neoadjuvant vs. adjuvant therapy
2. Node positive and node negative breast cancer
3. Hormone receptor-positive (HR+) and hormone receptor-negative (HR-) breast cancer
4. Large (≥2 cm) and small (<2 cm) tumour size

***Neoadjuvant vs. adjuvant therapy***

A step-wise approach is recommended for the treatment of EBC that often includes neoadjuvant therapy, surgery, and adjuvant therapy. Therefore, in studies that aim to investigate the impact of systemic therapy in the adjuvant setting, patients may have already received some form of systemic therapy neoadjuvantly. Unfortunately, the specific details of therapy received neoadjuvantly are not always reported. We classified studies as either neoadjuvant, adjuvant, or both neoadjuvant/adjuvant, based on the timing of therapy administered during the study. For example, patients in several trials had received neoadjuvant therapy and surgery prior to randomization, but they received adjuvant therapy during the trial, so these trials were classified as adjuvant. There were only six studies that included neoadjuvant therapy explicitly in the study design (HannaH,^10^ NeoALTTO,^13^ NOAH,^6^ NSABP B-41,^36^ GeparTrio,^22^ and Bayraktar 2012^33^). Two of these studies (GeparTrio^22^ and Bayraktar 2012^33^) focused primarily on neoadjuvant therapy, although patients also received adjuvant therapy consisting of radiotherapy and hormonal therapy. Radiotherapy and hormonal therapy were not included as separate nodes in our evidence networks, so these two studies were classified as neoadjuvant. H is usually administered for 52 weeks in both the neoadjuvant and adjuvant settings.^44,45^ In addition, most neoadjuvant studies use pCR as a surrogate endpoint and do not commonly report OS data. Since an evidence network based solely on neoadjuvant studies would result in a disconnected network, the evidence networks generated included both neoadjuvant/adjuvant therapies; most included studies focused on therapy in the adjuvant setting and based on standard therapy guidelines it is likely that most of these patients had received prior neoadjuvant therapy.^44,45^ Therefore, we did not perform a subgroup analysis comparing neoadjuvant versus adjuvant therapy due to a lack of information about neoadjuvant therapies, a lack of OS data, and disconnected evidence networks. Although our evidence networks combined neoadjuvant and adjuvant therapies, we do not expect substantial heterogeneity to have been introduced, given that most studies included both neoadjuvant and adjuvant treatments.

***Node positive and node negative breast cancer***

Evidence networks for this subgroup analysis are shown in Figure 8 (interactive figure online: <https://goo.gl/ppkLrG>), and the studies included in the analyses are shown in Table 15. For the node positive subgroup, no data were available for the 2008 time point, and for the node negative subgroup, no data were available for the 2008 or 2010 time points.

Fig. 8 Cumulative NMA evidence networks for overall survival for node subgroups. (A) Node positive subgroup, (B) node negative subgroup.

**A**


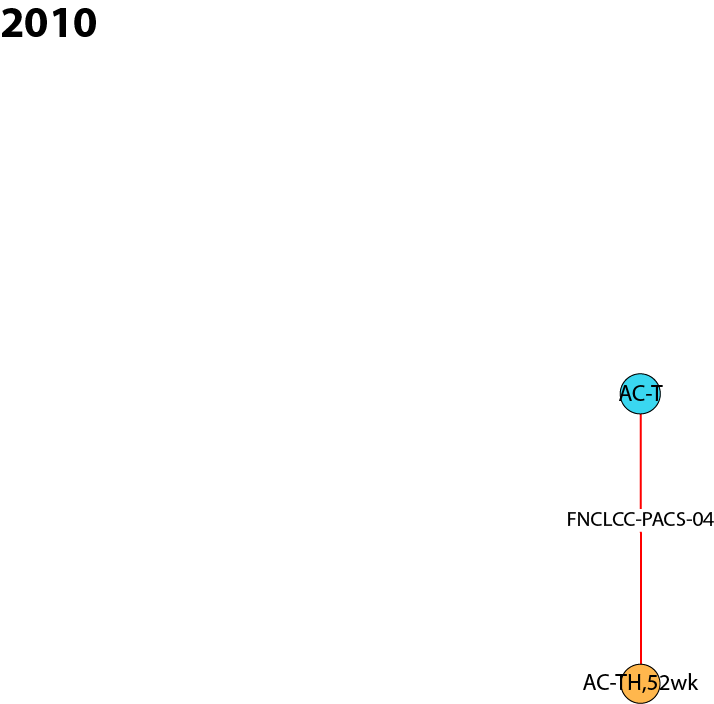


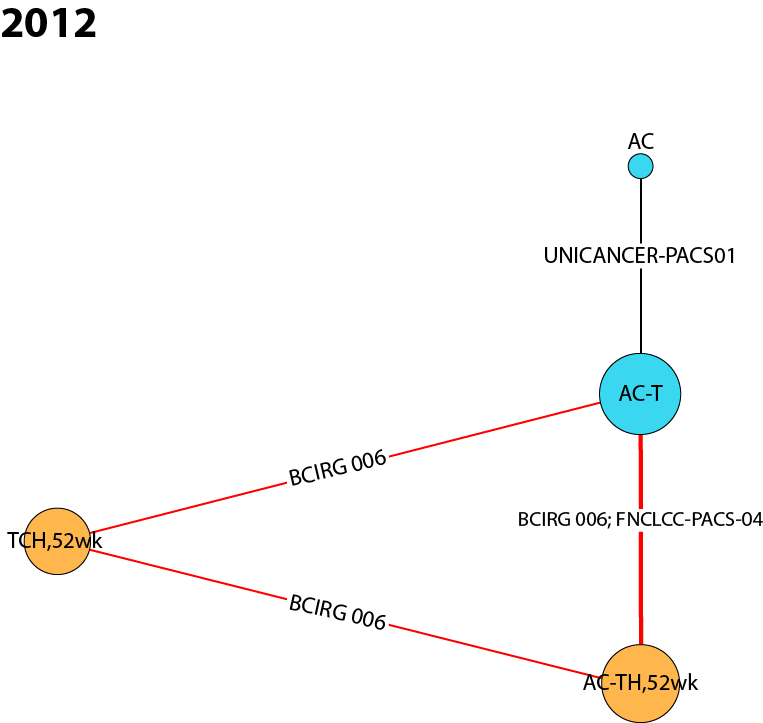


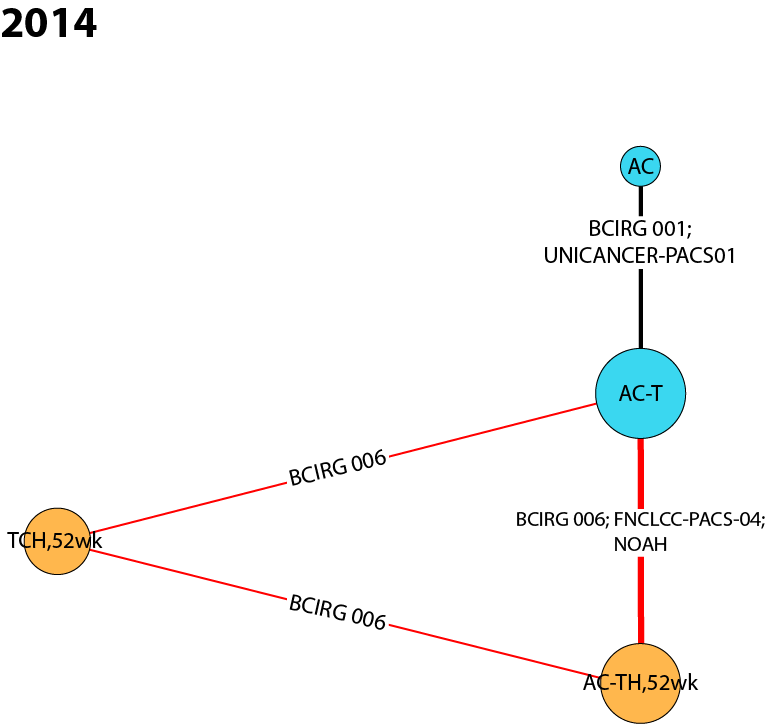


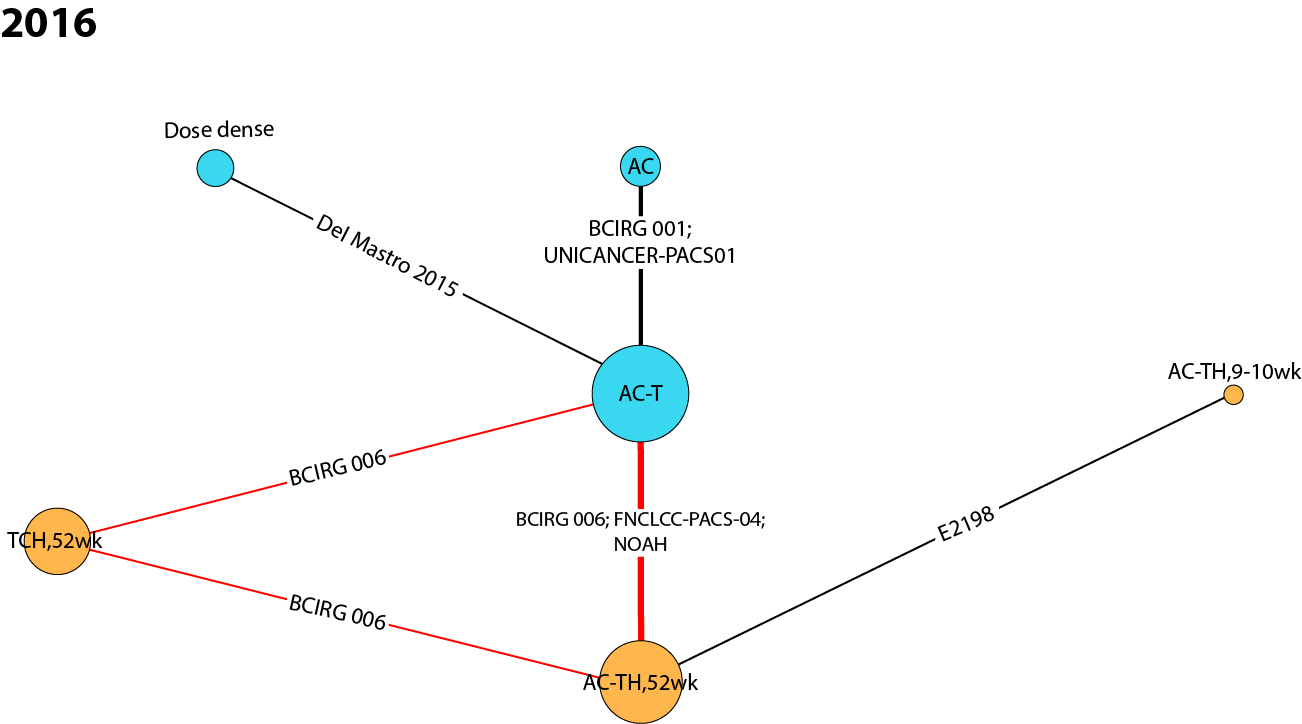


**B**


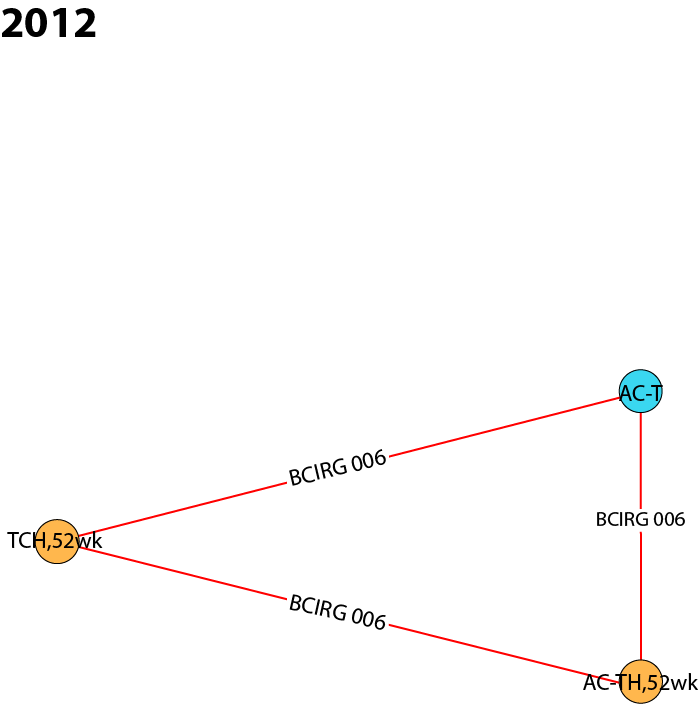


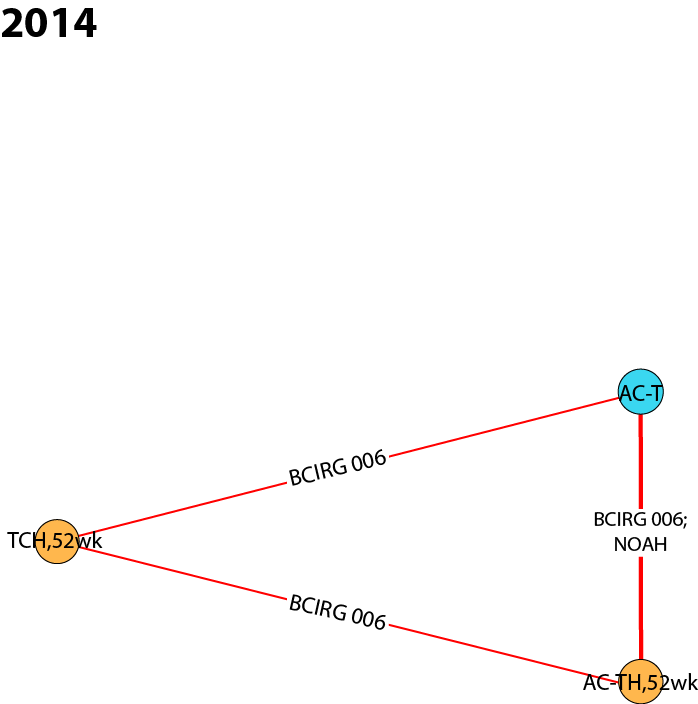


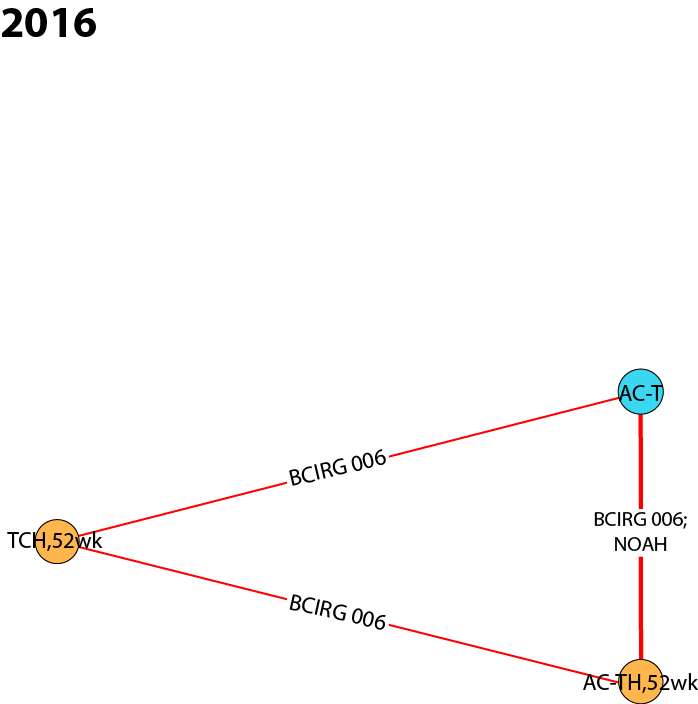


*AC* anthracycline (doxorubicin, epirubicin) + cyclophosphamide, *Dose dense* AC → T, or AC, either weekly or biweekly, *H* Herceptin® intravenous (IV), *T* taxane (docetaxel, paclitaxel), *TCH* docetaxel + carboplatin + Herceptin® IV, *wk* weeks.

Table 15 Summary of studies included in node positive and node negative subgroup analyses

|  | **2008** | **2010** | **2012** | **2014** | **2016** |
| --- | --- | --- | --- | --- | --- |
| **Node positive Subgroup** | | | | | |
| RCTs: 100% HER2+ | - | - | BCIRG 006  (Slamon 2011)^8^ | BCIRG 006  (Slamon 2011)^8^ | BCIRG 006  (Slamon 2011)^8^ |
|  | - | FNCLCC-PACS 04  (Spielmann 2009)^9^ | FNCLCC-PACS 04  (Spielmann 2009)^9^ | FNCLCC-PACS 04  (Spielmann 2009)^9^ | FNCLCC-PACS 04  (Spielmann 2009)^9^ |
|  | - | - | - | NOAH  (Gianni 2014)^6^ | NOAH  (Gianni 2014)^6^ |
| RCTs: HER2+ subgroups | - | - | - | BCIRG 001  (Mackey 2013)^16^ | BCIRG 001  (Mackey 2013)^16^ |
|  | - | - | - | - | Del Mastro 2015^26^ |
|  | - | - | - | - | E2198  (Schneider 2015)^19^ |
|  | - | - | UNICANCER-PACS-01  (Coudert 2012)^25^ | UNICANCER-PACS-01  (Coudert 2012)^25^ | UNICANCER-PACS-01  (Coudert 2012)^25^ |
| **Node negative Subgroup** | | | | | |
| RCTs: 100% HER2+ | - | - | BCIRG 006  (Slamon 2011)^8^ | BCIRG 006  (Slamon 2011)^8^ | BCIRG 006  (Slamon 2011)^8^ |
|  | - | - | - | NOAH  (Gianni 2014)^6^ | NOAH  (Gianni 2014)^6^ |

*HER2+* human epidermal growth factor receptor 2-positive, *RCT* randomized controlled trial.

For the pairwise comparison AC-TH_52wk_ vs. AC-T, initial evidence in 2010 for the node positive subgroup is based entirely on data from the FNCLCC-PACS 04 trial (Spielmann 2009), resulting in an effect estimate with a wide credible interval and in favour of chemotherapy alone (2010 HR 1.27, 95% CrI 0.68 to 2.38). However, with the incorporation of more data in 2012, 2014, and 2016, the effect estimate shifts to favour H/chemotherapy and the certainty in the survival benefit becomes stronger (2016 HR 0.73, 95% CrI 0.58 to 0.91). For the node negative subgroup, there is a consistent OS advantage for patients receiving H/chemotherapy compared with chemotherapy alone, and all effect estimates are statistically significant, although there is less precision (2012 HR 0.38, 95% CrI 0.17 to 0.87; 2016 HR 0.44, 95% CrI 0.23 to 0.85).

For the pairwise comparison TCH_52wk_ vs. AC-T, there is an OS advantage for H/chemotherapy versus chemotherapy alone for both the node positive and node negative subgroups. The effect estimates were very similar across all three time points within the node positive analysis (2016 HR 0.81, 95% CrI 0.63 to 1.06) and within the node negative analysis (2016 HR 0.56, 95% CrI 0.27 to 1.14). No results were statistically significant, but a more pronounced OS advantage was observed for the node negative subgroup (lower HR point estimate), although the estimates were less precise compared with the node positive subgroup.

For the pairwise comparison AC-TH_52wk_ vs. TCH_52wk_, there appears to be no clear significant difference in OS for node positive (2016 HR 0.89, 95% CrI 0.63 to 1.25) or node negative patients (2016 HR 0.79, 95% CrI 0.30 to 2.08). Although the effect estimates from both analyses favoured AC-TH_52wk_ compared with TCH_52wk_, no statistical significance was observed, and there were very wide credible intervals for the node negative subgroup.

Wider credible intervals were observed for the node negative subgroup compared with the node positive subgroup, which is likely due to the smaller evidence networks and smaller sample size, as clinical studies tend not to focus exclusively on node negative patients. Only two trials (three treatments) were available for the node negative subgroup, compared with seven trials (six treatments) for the node positive subgroup.

***Hormone receptor-positive (HR+) and hormone receptor-negative (HR-) breast cancer***

Evidence networks for this subgroup analysis are shown in Figure 9 (interactive figure online: <https://goo.gl/ppkLrG>), and the studies included in the analyses are shown in Table 16. The same studies were included in both the HR+ and HR- analyses, although the subgroup sample sizes are different. No data were available for the 2008 or 2010 time points. The final evidence networks in 2016 include three nodes connected by four RCTs (three publications).

Fig. 9 Cumulative NMA evidence networks for overall survival for hormone receptor subgroups. (A) HR+ subgroup, (B) HR- subgroup.

**A B**


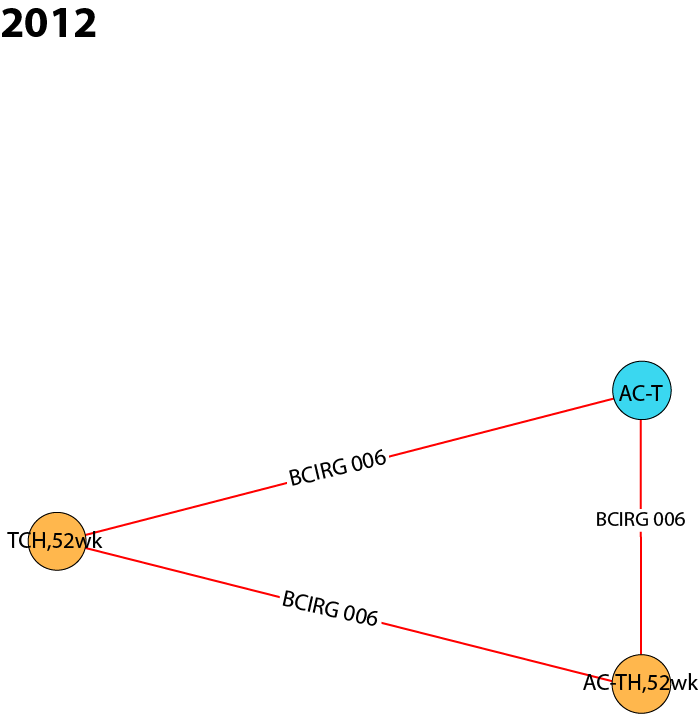


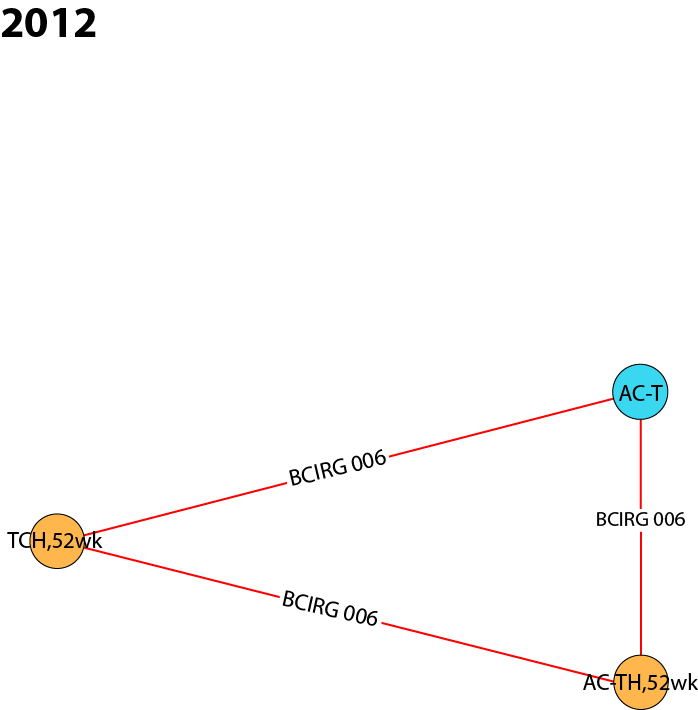


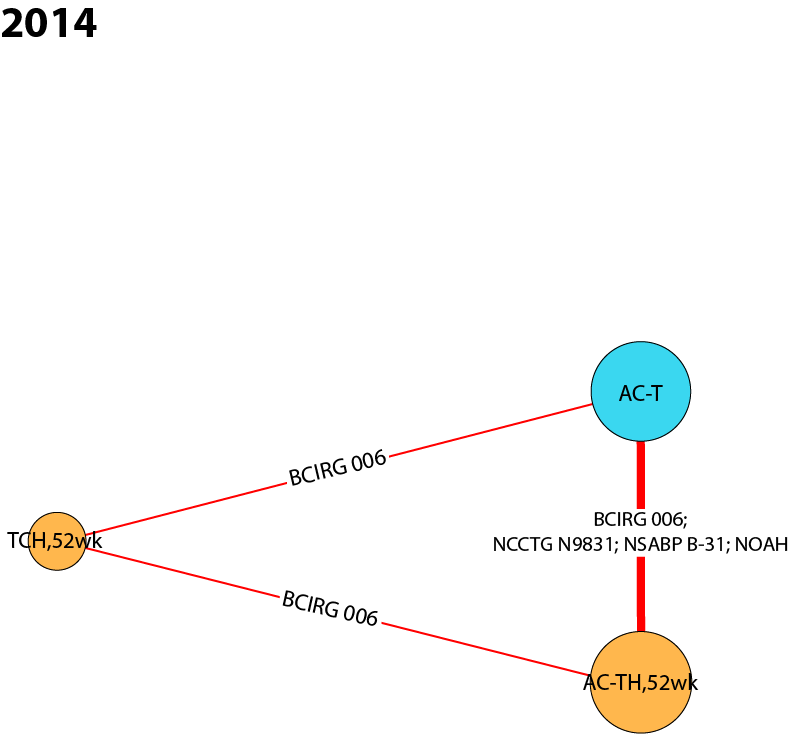


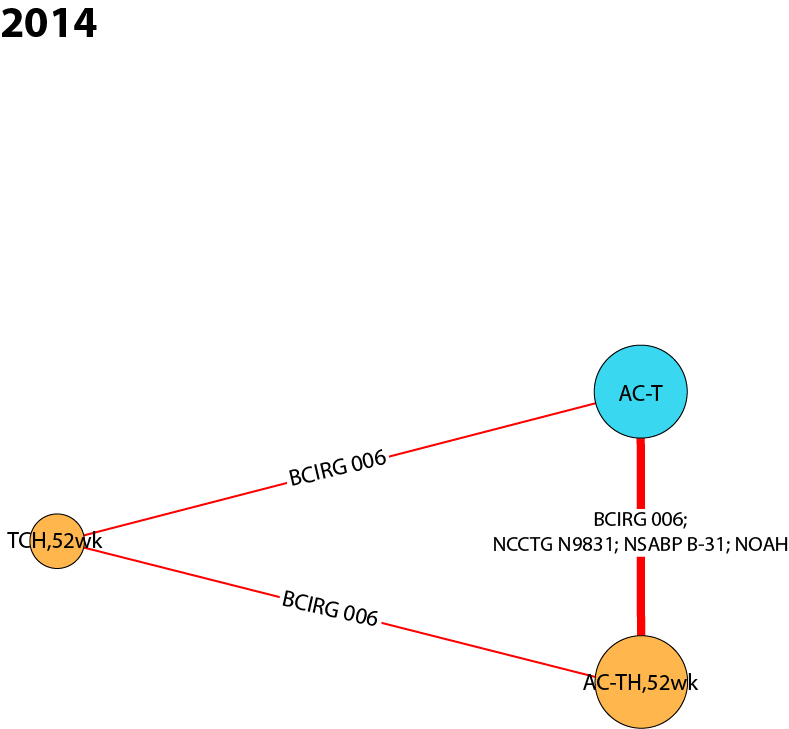


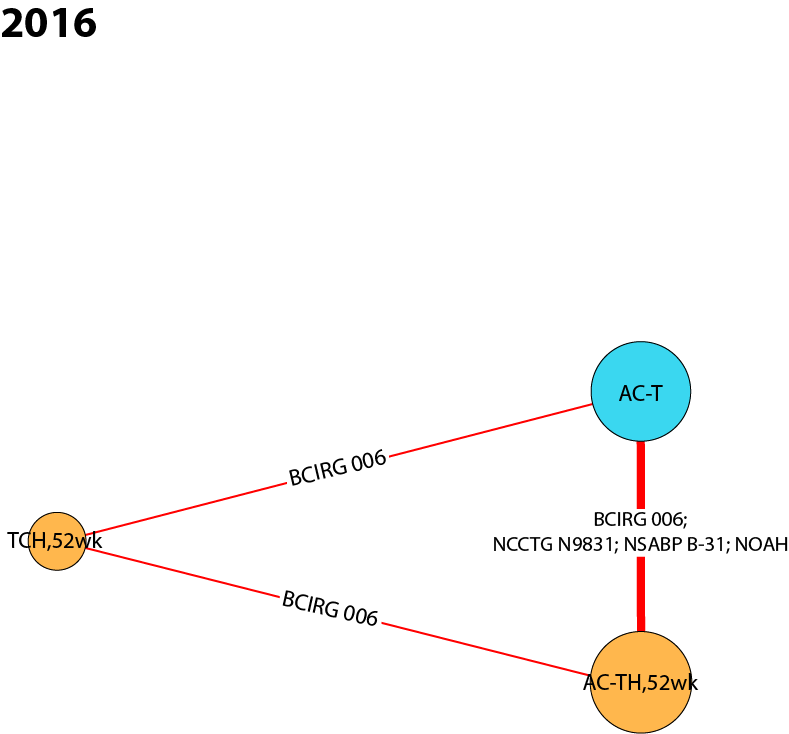


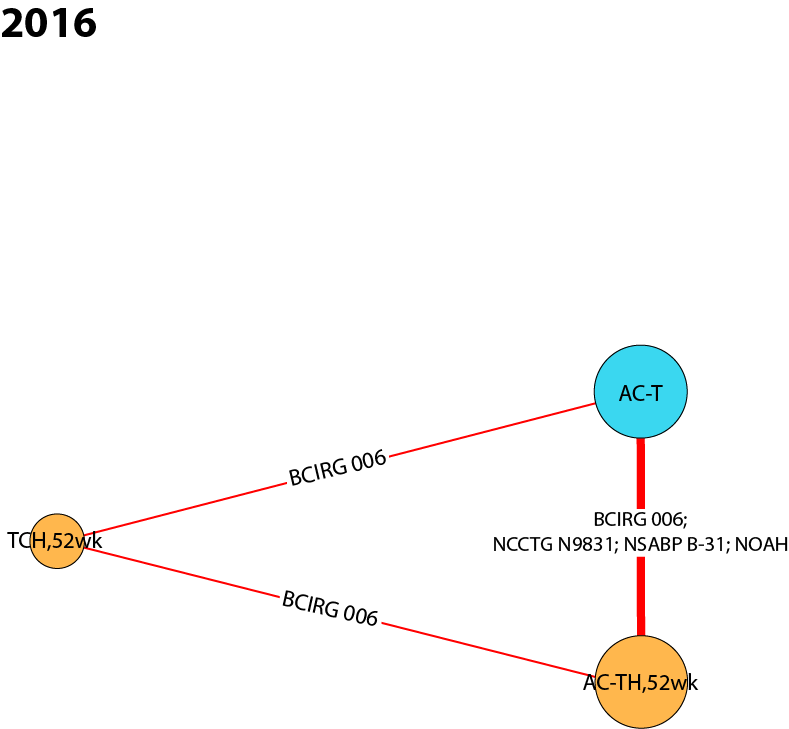


*AC* anthracycline (doxorubicin, epirubicin) + cyclophosphamide, *H* Herceptin® intravenous (IV), *T* taxane (docetaxel, paclitaxel), *TCH* docetaxel + carboplatin + Herceptin® IV, *wk* weeks.

Table 16 Summary of studies included in HR+ and HR- subgroup analyses

|  | **2008** | **2010** | **2012** | **2014** | **2016** |
| --- | --- | --- | --- | --- | --- |
| RCTs: 100% HER2+ | - | - | BCIRG 006  (Slamon 2011)^8^ | BCIRG 006  (Slamon 2011)^8^ | BCIRG 006  (Slamon 2011)^8^ |
|  | - | - | - | NCCTG N9831 & NSABP B-31  (Perez 2014)^12^ | NCCTG N9831 & NSABP B-31  (Perez 2014)^12^ |
|  | - | - | - | NOAH  (Gianni 2014)^6^ | NOAH  (Gianni 2014)^6^ |

*HER2+* human epidermal growth factor receptor 2-positive, *HR* hormone receptor, *RCT* randomized controlled trial.

For the pairwise comparison AC-TH_52wk_ vs. AC-T, initial evidence in 2012 was very similar for the HR+ subgroup (HR 0.62, 95% CrI 0.42 to 0.92) and the HR- subgroup (HR 0.63, 95% CrI 0.45 to 0.89), and demonstrated an OS advantage for H/chemotherapy compared with chemotherapy alone. The certainty in these survival benefits strengthened over time, and the effect estimates remained similar and statistically significant for both subgroups in 2016 (HR+ subgroup: HR 0.64, 95% CrI 0.53 to 0.77; HR- subgroup: HR 0.63, 95% CrI 0.53 to 0.75).

For the pairwise comparison TCH_52wk_ vs. AC-T, there appears to be no significant difference in OS for HR+ patients who received TCH_52wk_ compared with AC-T, with effect estimates remaining constant from 2012 (HR 0.95, 95% CrI 0.67 to 1.37) to 2016 (HR 0.95, 95% CrI 0.66 to 1.36). However, for the HR- subgroup, there is an OS advantage for H/taxane-based chemotherapy compared with chemotherapy alone, with consistent statistically significant differences for all three time points (2016 HR 0.63, 95% CrI 0.45 to 0.88).

For the pairwise comparison AC-TH_52wk_ vs. TCH_52wk_, there is an OS advantage for AC-TH_52wk_ compared with TCH_52wk_ for the HR+ subgroup, resulting in a statistically significant effect estimate in 2016 (HR 0.67, 95% CrI 0.45 to 0.99). However, there appears to be no significant difference in OS for HR- patients, with all three effect estimates hovering around equivalence (2016 HR 1.00, 95% CrI 0.69 to 1.47).

***Large (≥2 cm) and small (<2 cm) tumour size***

Evidence networks for this subgroup analysis are shown in Figure 10 (interactive figure online: <https://goo.gl/ppkLrG>), and the studies included in the analyses are shown in Table 17. No data were available for the 2008 or 2010 time points. For the large tumours (≥2 cm) subgroup, the 2016 evidence network included five nodes connected by five RCTs (four publications). For the small tumours (<2 cm) subgroup, the 2016 evidence network included three nodes connected by three RCTs (two publications).

Fig. 10 Cumulative NMA evidence networks for overall survival for subgroups based on tumour size. (A) Large tumours (≥2 cm) subgroup, (B) small tumours (<2 cm) subgroup.

**A**


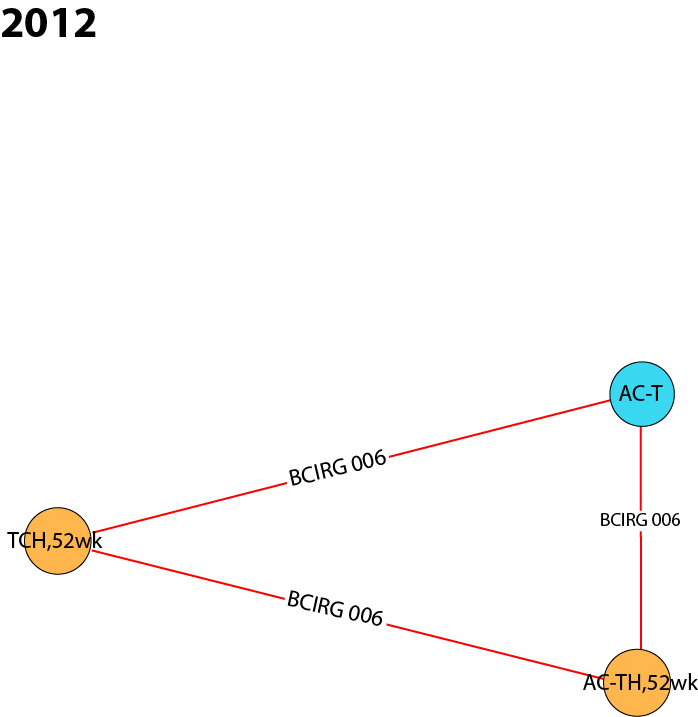


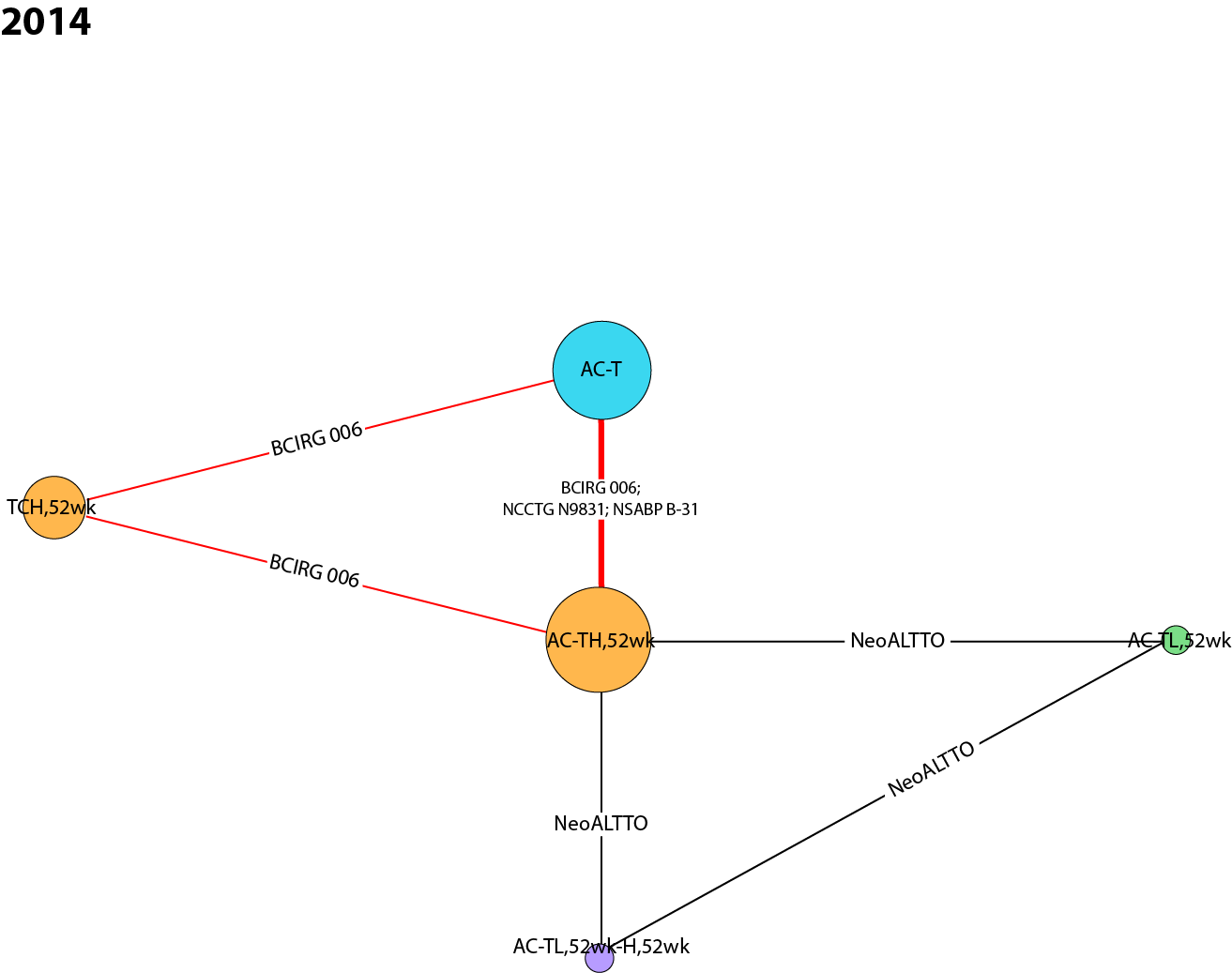


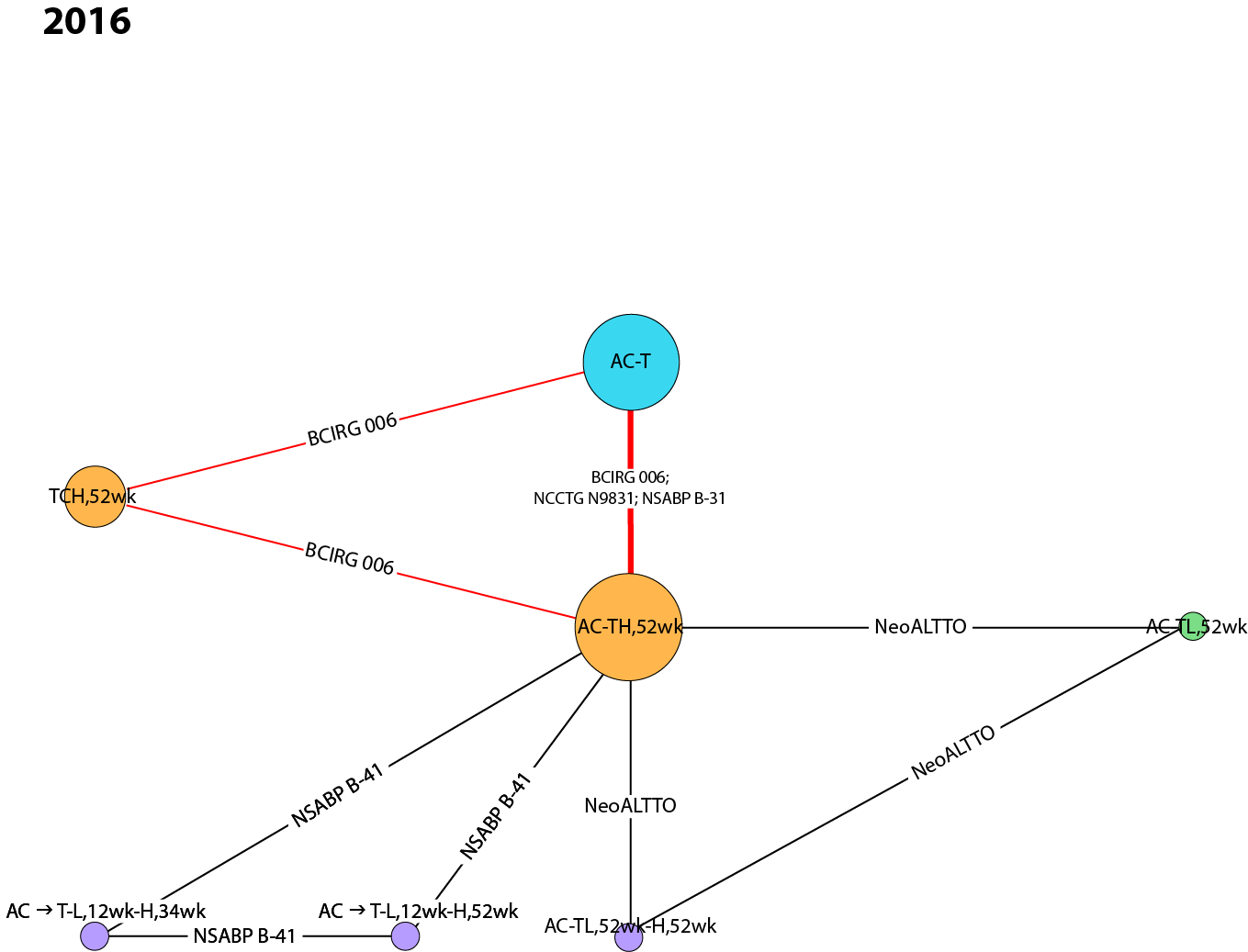


**B**


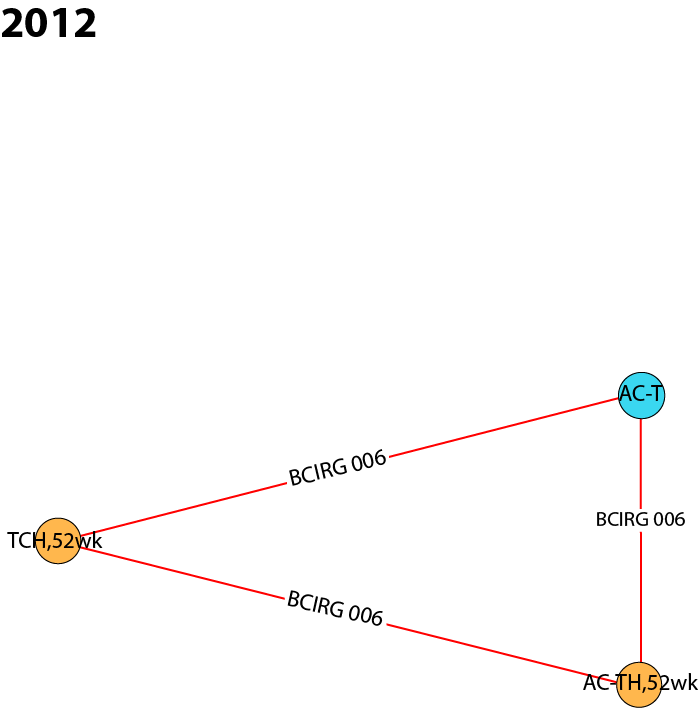


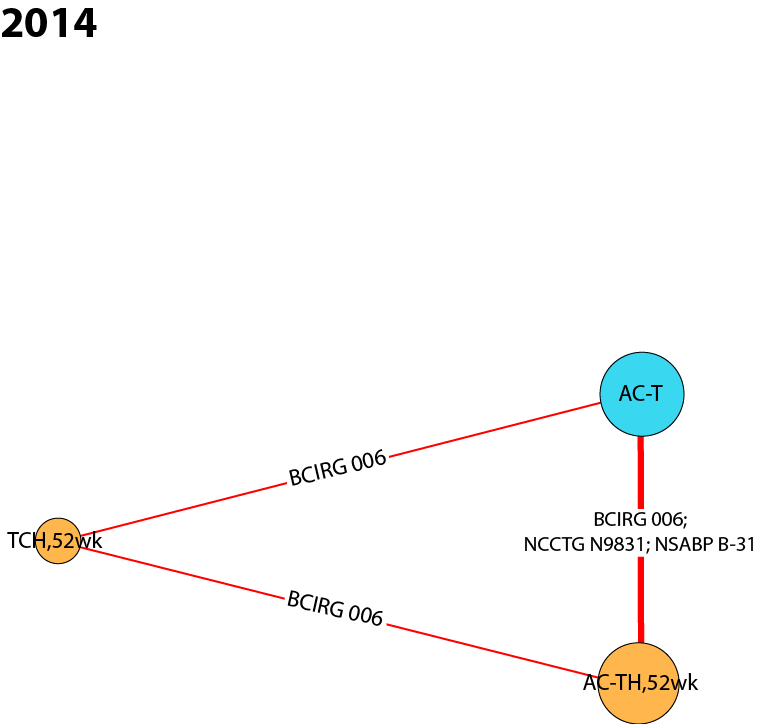


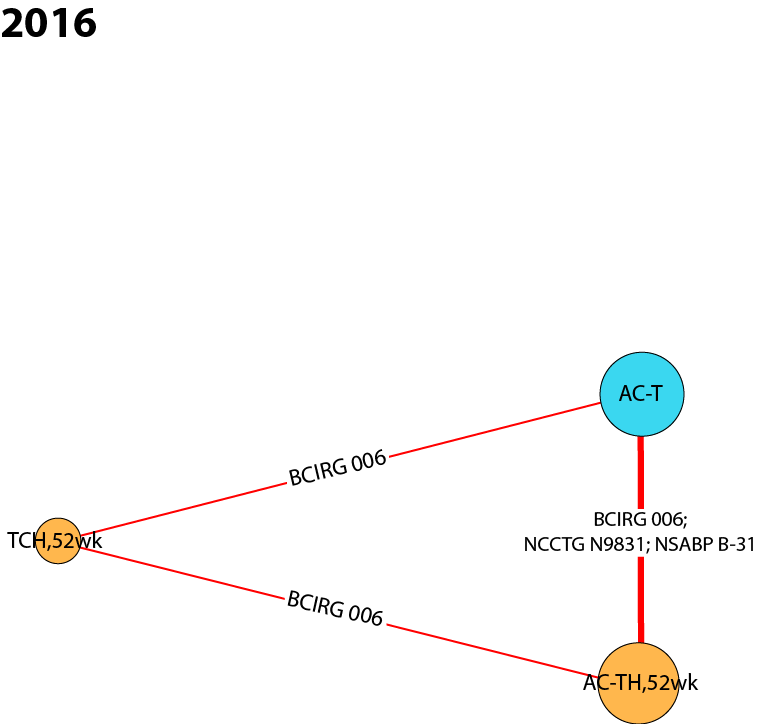


*AC* anthracycline (doxorubicin, epirubicin) + cyclophosphamide, *Dose dense* AC → T, or AC, either weekly or biweekly, *H* Herceptin® intravenous (IV), *L* lapatinib, *T* taxane (docetaxel, paclitaxel), *TCH* docetaxel + carboplatin + Herceptin® IV, *wk* weeks.

Table 17 Summary of studies included in large (≥2 cm) and small (<2 cm) tumour size subgroup analyses

|  | **2008** | **2010** | **2012** | **2014** | **2016** |
| --- | --- | --- | --- | --- | --- |
| **Large tumour (≥2cm) subgroup** | | | | | |
| RCTs: 100% HER2+ | - | - | BCIRG 006  (Slamon 2011)^8^ | BCIRG 006  (Slamon 2011)^8^ | BCIRG 006  (Slamon 2011)^8^ |
|  | - | - | - | NCCTG N9831 & NSABP B-31  (Perez 2014)^12^ | NCCTG N9831 & NSABP B-31  (Perez 2014)^12^ |
|  | - | - | - | NeoALTTO (de Azambuja 2014)^13^ | NeoALTTO (de Azambuja 2014)^13^ |
|  | - | - | - | - | NSABP B-41 (Robidoux 2016)^36^ |
| **Small tumour (<2cm) subgroup** | | | | | |
| RCTs: 100% HER2+ | - | - | BCIRG 006  (Slamon 2011)^8^ | BCIRG 006  (Slamon 2011)^8^ | BCIRG 006  (Slamon 2011)^8^ |
|  | - | - | - | NCCTG N9831 & NSABP B-31  (Perez 2014)^12^ | NCCTG N9831 & NSABP B-31  (Perez 2014)^12^ |

*HER2+* human epidermal growth factor receptor 2-positive, *RCT* randomized controlled trial.

For the pairwise comparison AC-TH_52wk_ vs. AC-T, there is an OS advantage for H/chemotherapy versus chemotherapy alone for both subgroups, and the precision around the effect estimates improved over time. All effect estimates were statistically significant for the subgroup of patients with large tumours ≥2 cm (2012 HR 0.66, 95% CrI 0.49 to 0.88; 2016 HR 0.66, 95% CrI 0.56 to 0.79) and for the subgroup of patients with small tumours <2 cm (2012 HR 0.49, 95% CrI 0.27 to 0.91; 2016 HR 0.51, 95% CrI 0.39 to 0.66).

A similar trend was observed for the pairwise comparison TCH_52wk_ vs. AC-T, with an OS advantage for H/chemotherapy compared with chemotherapy alone for both subgroups; however, no statistical significance was observed. The effect estimates for this comparison are virtually identical within each subgroup (large tumours: HR 0.77, 95% CrI 0.58 to 1.02; small tumours: HR 0.75, 95% CrI 0.43 to 1.30), as direct evidence for this comparison is derived from only one RCT (BCIRG 006) and the evidence networks are relatively small.

For the pairwise comparison AC-TH_52wk_ vs. TCH_52wk_, there is an OS advantage for AC-TH_52wk_ compared with TCH_52wk_ for both subgroups, although no comparisons are statistically significant. There is a slight improvement in precision over time for both the large tumours subgroup (2012 HR 0.86, 95% CrI 0.58 to 1.27; 2016 HR 0.86, 95% CrI 0.62 to 1.19) and the small tumours subgroup (2012 HR 0.65, 95% CrI 0.29 to 1.47; 2016 HR 0.68, 95% CrI 0.37 to 1.23).

PRISMA NMA Checklist

Table 18 PRISMA NMA checklist of items to include when reporting a systematic review involving a network meta-analysis

| **Section/Topic** | **Item #** | **Checklist Item** | **Reported on Page #** |
| --- | --- | --- | --- |
| **TITLE** |  |  |  |
| Title | 1 | Identify the report as a systematic review *incorporating a network meta-analysis (or related form of meta-analysis).* | 1 |
|  |  |  |  |
| **ABSTRACT** |  |  |  |
| Structured summary | 2 | Provide a structured summary including, as applicable:  **Background:** main objectives  **Methods:** data sources; study eligibility criteria, participants, and interventions; study appraisal; and *synthesis methods, such as network meta-analysis.*  **Results:** number of studies and participants identified; summary estimates with corresponding confidence/credible intervals; *treatment rankings may also be discussed. Authors may choose to summarize pairwise comparisons against a chosen treatment included in their analyses for brevity.*  **Discussion/Conclusions:** limitations; conclusions and implications of findings.  **Other:** primary source of funding; systematic review registration number with registry name. | 2 |
|  |  |  |  |
| **INTRODUCTION** |  |  |  |
| Rationale | 3 | Describe the rationale for the review in the context of what is already known*, including mention of why a network meta-analysis has been conducted.* | 3 |
| Objectives | 4 | Provide an explicit statement of questions being addressed, with reference to population, interventions, comparisons, outcomes, and study design (PICOS). | 3 |
|  |  |  |  |
| **METHODS** |  |  |  |
| Protocol and registration | 5 | Indicate whether a review protocol exists and if and where it can be accessed (e.g., Web address); and, if available, provide registration information, including registration number. | 4 |
| Eligibility criteria | 6 | Specify study characteristics (e.g., PICOS, length of follow-up) and report characteristics (e.g., years considered, language, publication status) used as criteria for eligibility, giving rationale. *Clearly describe eligible treatments included in the treatment network, and note whether any have been clustered or merged into the same node (with justification).* | 4-5 |
| Information sources | 7 | Describe all information sources (e.g., databases with dates of coverage, contact with study authors to identify additional studies) in the search and date last searched. | 4-5 |
| Search | 8 | Present full electronic search strategy for at least one database, including any limits used, such that it could be repeated. | 4-5 |
| Study selection | 9 | State the process for selecting studies (i.e., screening, eligibility, included in systematic review, and, if applicable, included in the meta-analysis). | 4-5 |
| Data collection process | 10 | Describe method of data extraction from reports (e.g., piloted forms, independently, in duplicate) and any processes for obtaining and confirming data from investigators. | 4-5 |
| Data items | 11 | List and define all variables for which data were sought (e.g., PICOS, funding sources) and any assumptions and simplifications made. | 4-5 |
| Geometry of the network | **S1** | Describe methods used to explore the geometry of the treatment network under study and potential biases related to it. This should include how the evidence base has been graphically summarized for presentation, and what characteristics were compiled and used to describe the evidence base to readers. | 5 |
| Risk of bias within individual studies | 12 | Describe methods used for assessing risk of bias of individual studies (including specification of whether this was done at the study or outcome level), and how this information is to be used in any data synthesis. | 5 |
| Summary measures | 13 | State the principal summary measures (e.g., risk ratio, difference in means). *Also describe the use of additional summary measures assessed, such as treatment rankings and surface under the cumulative ranking curve (SUCRA) values, as well as modified approaches used to present summary findings from meta-analyses.* | 5 |
| Planned methods of analysis | 14 | Describe the methods of handling data and combining results of studies for each network meta-analysis. This should include, but not be limited to:   - *Handling of multi-arm trials;* - *Selection of variance structure;* - *Selection of prior distributions in Bayesian analyses; and* - *Assessment of model fit.* | 6 |
| Assessment of Inconsistency | **S2** | Describe the statistical methods used to evaluate the agreement of direct and indirect evidence in the treatment network(s) studied. Describe efforts taken to address its presence when found. | 7 |
| Risk of bias across studies | 15 | Specify any assessment of risk of bias that may affect the cumulative evidence (e.g., publication bias, selective reporting within studies). | 6 |
| Additional analyses | 16 | Describe methods of additional analyses if done, indicating which were pre-specified. This may include, but not be limited to, the following:   - Sensitivity or subgroup analyses; - Meta-regression analyses; - *Alternative formulations of the treatment network; and* - *Use of alternative prior distributions for Bayesian analyses (if applicable).* | 7 |
|  |  |  |  |
| **RESULTS**** |  |  |  |
| Study selection | 17 | Give numbers of studies screened, assessed for eligibility, and included in the review, with reasons for exclusions at each stage, ideally with a flow diagram. | 8, Fig 1 |
| **Presentation of network structure** | **S3** | Provide a network graph of the included studies to enable visualization of the geometry of the treatment network. | 8, Fig 2 |
| **Summary of network geometry** | **S4** | Provide a brief overview of characteristics of the treatment network. This may include commentary on the abundance of trials and randomized patients for the different interventions and pairwise comparisons in the network, gaps of evidence in the treatment network, and potential biases reflected by the network structure. | 8 |
| Study characteristics | 18 | For each study, present characteristics for which data were extracted (e.g., study size, PICOS, follow-up period) and provide the citations. | 8-9, Table 1 |
| Risk of bias within studies | 19 | Present data on risk of bias of each study and, if available, any outcome level assessment. | 8-9, Appendix |
| Results of individual studies | 20 | For all outcomes considered (benefits or harms), present, for each study: 1) simple summary data for each intervention group, and 2) effect estimates and confidence intervals. *Modified approaches may be needed to deal with information from larger networks.* | Appendix |
| Synthesis of results | 21 | Present results of each meta-analysis done, including confidence/credible intervals. *In larger networks, authors may focus on comparisons versus a particular comparator (e.g., placebo or standard care), with full findings presented in an appendix. League tables and forest plots may be considered to summarize pairwise comparisons.* If additional summary measures were explored (such as treatment rankings), these should also be presented. | 8-9, Fig 3, Appendix |
| **Exploration for inconsistency** | **S5** | Describe results from investigations of inconsistency. This may include such information as measures of model fit to compare consistency and inconsistency models, *P* values from statistical tests, or summary of inconsistency estimates from different parts of the treatment network. | 8-9 |
| Risk of bias across studies | 22 | Present results of any assessment of risk of bias across studies for the evidence base being studied. | 9 |
| Results of additional analyses | 23 | Give results of additional analyses, if done (e.g., sensitivity or subgroup analyses, meta-regression analyses*, alternative network geometries studied, alternative choice of prior distributions for Bayesian analyses,* and so forth). | 10, Fig 4, Appendix |
|  |  |  |  |
| **DISCUSSION** |  |  |  |
| Summary of evidence | 24 | Summarize the main findings, including the strength of evidence for each main outcome; consider their relevance to key groups (e.g., healthcare providers, users, and policy-makers). | 11-12 |
| Limitations | 25 | Discuss limitations at study and outcome level (e.g., risk of bias), and at review level (e.g., incomplete retrieval of identified research, reporting bias). *Comment on the validity of the assumptions, such as transitivity and consistency. Comment on any concerns regarding network geometry (e.g., avoidance of certain comparisons).* | 13-14 |
| Conclusions | 26 | Provide a general interpretation of the results in the context of other evidence, and implications for future research. | 15 |
| **FUNDING** |  |  | 2,8 |
| Funding | 27 | Describe sources of funding for the systematic review and other support (e.g., supply of data); role of funders for the systematic review. This should also include information regarding whether funding has been received from manufacturers of treatments in the network and/or whether some of the authors are content experts with professional conflicts of interest that could affect use of treatments in the network. |  |

*PICOS* population, intervention, comparators, outcomes, study design.

*Text in italics indicates wording specific to reporting of network meta-analyses that has been added to guidance from the PRISMA statement.

**Authors may wish to plan for use of appendices to present all relevant information in full detail for items in this section.

# REFERENCES

1. Ross JS, Slodkowska EA, Symmans WF, Pusztai L, Ravdin PM, Hortobagyi GN. The HER-2 receptor and breast cancer: ten years of targeted anti-HER-2 therapy and personalized medicine. *Oncologist* 2009; **14:** 320–68.

2. Goldhirsch A, Gelber RD, Piccart-Gebhart MJ, et al. 2 years versus 1 year of adjuvant trastuzumab for HER2-positive breast cancer (HERA): an open-label, randomised controlled trial. *Lancet* 2013; **382:** 1021–28.

3. Smith I, Procter M, Gelber RD, et al. 2-year follow-up of trastuzumab after adjuvant chemotherapy in HER2-positive breast cancer: a randomised controlled trial. *Lancet* 2007; **369:** 29–36.

4. Gianni L, Dafni U, Gelber RD, et al. Treatment with trastuzumab for 1 year after adjuvant chemotherapy in patients with HER2-positive early breast cancer: a 4-year follow-up of a randomised controlled trial. *Lancet Oncol* 2011; **12:** 236–44.

5. Piccart-Gebhart M, Holmes E, Baselga J, et al. Adjuvant lapatinib and trastuzumab for early human epidermal growth factor receptor 2-positive breast cancer: results from the randomized phase III adjuvant lapatinib and/or trastuzumab treatment optimization trial. *J Clin Oncol* 2016; **34:** 1034–42.

6. Gianni L, Eiermann W, Semiglazov V, et al. Neoadjuvant and adjuvant trastuzumab in patients with HER2-positive locally advanced breast cancer (NOAH): follow-up of a randomised controlled superiority trial with a parallel HER2-negative cohort. *Lancet Oncol* 2014; **15:** 640–47.

7. Gianni L, Eiermann W, Semiglazov V, et al. Neoadjuvant chemotherapy with trastuzumab followed by adjuvant trastuzumab versus neoadjuvant chemotherapy alone, in patients with HER2-positive locally advanced breast cancer (the NOAH trial): a randomised controlled superiority trial with a parallel HER2-negative cohort. *Lancet* 2010; **375:** 377–84.

8. Slamon D, Eiermann W, Robert N, et al. Adjuvant trastuzumab in HER2-positive breast cancer. *N Engl J Med* 2011; **365:** 1273–83.

9. Spielmann M, Roche H, Delozier T, et al. Trastuzumab for patients with axillary-node-positive breast cancer: results of the FNCLCC-PACS 04 trial. *J Clin Oncol* 2009; **27:** 6129–34.

10. Jackisch C, Hegg R, Stroyakovskiy D, et al. HannaH phase III randomised study: association of total pathological complete response with event-free survival in HER2-positive early breast cancer treated with neoadjuvant-adjuvant trastuzumab after 2 years of treatment-free follow-up. *Eur J Cancer* 2016; **62:** 62–75.

11. Mavroudis D, Saloustros E, Malamos N, et al. Six versus 12 months of adjuvant trastuzumab in combination with dose-dense chemotherapy for women with HER2-positive breast cancer: a multicenter randomized study by the Hellenic Oncology Research Group (HORG). *Ann Oncol* 2015; **26:** 1333–40.

12. Perez EA, Romond EH, Suman VJ, et al. Trastuzumab plus adjuvant chemotherapy for human epidermal growth factor receptor 2-positive breast cancer: planned joint analysis of overall survival from NSABP B-31 and NCCTG N9831. *J Clin Oncol* 2014; **32:** 3744–52.

13. de Azambuja E, Holmes AP, Piccart-Gebhart M, et al. Lapatinib with trastuzumab for HER2-positive early breast cancer (NeoALTTO): survival outcomes of a randomised, open-label, multicentre, phase 3 trial and their association with pathological complete response. *Lancet Oncol* 2014; **15:** 1137–46.

14. Robidoux A, Tang G, Rastogi P, et al. Lapatinib as a component of neoadjuvant therapy for HER2-positive operable breast cancer (NSABP protocol B-41): an open-label, randomised phase 3 trial. *Lancet Oncol* 2013; **14:** 1183–92.

15. Pivot X, Romieu G, Debled M, et al. 6 months versus 12 months of adjuvant trastuzumab for patients with HER2-positive early breast cancer (PHARE): a randomised phase 3 trial. *Lancet Oncol* 2013; **14:** 741–48.

16. Mackey JR, Martin M, Pienkowski T, et al. Adjuvant docetaxel, doxorubicin, and cyclophosphamide in node-positive breast cancer: 10-year follow-up of the phase 3 randomised BCIRG 001 trial. *Lancet Oncol* 2013; **14:** 72–80.

17. Earl HM, Hiller L, Dunn JA, et al. Adjuvant epirubicin followed by cyclophosphamide, methotrexate and fluorouracil (CMF) vs CMF in early breast cancer: results with over 7 years median follow-up from the randomised phase III NEAT/BR9601 trials. *British Journal of Cancer* 2012; **107:** 1257–67.

18. Sparano JA, Zhao F, Martino S, et al. Long-term follow-up of the E1199 phase III trial evaluating the role of taxane and schedule in operable breast cancer. *J Clin Oncol* 2015; **33:** 2353–60.

19. Schneider BP, O'Neill A, Shen F, et al. Pilot trial of paclitaxel-trastuzumab adjuvant therapy for early stage breast cancer: a trial of the ECOG-ACRIN cancer research group (E2198). *British Journal of Cancer* 2015; **113:** 1651–57.

20. Joensuu H, Bono P, Kataja V, et al. Fluorouracil, epirubicin, and cyclophosphamide with either docetaxel or vinorelbine, with or without trastuzumab, as adjuvant treatments of breast cancer: final results of the FinHer Trial. *J Clin Oncol* 2009; **27:** 5685–92.

21. Joensuu H, Kellokumpu-Lehtinen PL, Huovinen R, et al. Outcome of patients with HER2-positive breast cancer treated with or without adjuvant trastuzumab in the Finland Capecitabine Trial (FinXX). *Acta Oncologica (Stockholm, Sweden)* 2014; **53:** 186–94.

22. von Minckwitz G, Blohmer JU, Costa SD, et al. Response-guided neoadjuvant chemotherapy for breast cancer. *J Clin Oncol* 2013; **31:** 3623–30.

23. Del Mastro L, Bruzzi P, Nicolo G, et al. HER2 expression and efficacy of dose-dense anthracycline-containing adjuvant chemotherapy in breast cancer patients. *British Journal of Cancer* 2005; **93:** 7–14.

24. Goss PE, Smith IE, O'Shaughnessy J, et al. Adjuvant lapatinib for women with early-stage HER2-positive breast cancer: a randomised, controlled, phase 3 trial. *Lancet Oncol* 2013; **14:** 88–96.

25. Coudert B, Asselain B, Campone M, et al. Extended benefit from sequential administration of docetaxel after standard fluorouracil, epirubicin, and cyclophosphamide regimen for node-positive breast cancer: the 8-year follow-up results of the UNICANCER-PACS01 trial. *Oncologist* 2012; **17:** 900–09.

26. Del Mastro L, De PS, Bruzzi P, et al. Fluorouracil and dose-dense chemotherapy in adjuvant treatment of patients with early-stage breast cancer: An open-label, 2 x 2 factorial, randomised phase 3 trial. *Lancet* 2015; **385:** 1863–72.

27. Rocca A, Bravaccini S, Scarpi E, et al. Benefit from anthracyclines in relation to biological profiles in early breast cancer. *Breast Cancer Res Treat* 2014; **144:** 307–18.

28. Boccardo F, Amadori D, Guglielmini P, et al. Epirubicin followed by cyclophosphamide, methotrexate and 5-fluorouracil versus paclitaxel followed by epirubicin and vinorelbine in patients with high-risk operable breast cancer. *Oncology* 2010; **78:** 274–81.

29. Miles DW, Harris WH, Gillett CE, Smith P, Barnes DM. Effect of c-erbB(2) and estrogen receptor status on survival of women with primary breast cancer treated with adjuvant cyclophosphamide/methotrexate/fluorouracil. *International Journal of Cancer* 1999; **84:** 354–59.

30. Colozza M, Sidoni A, Mosconi AM, et al. HER2 overexpression as a predictive marker in a randomized trial comparing adjuvant cyclophosphamide/methotrexate/5-fluorouracil with epirubicin in patients with stage I/II breast cancer: long-term results. *Clin Breast Cancer* 2005; **6:** 253–59.

31. Sterne JAC, Higgins JPT, Reeves BC. on behalf of the development group for ROBINS-I. ROBINS-I: a tool for assessing Risk Of Bias In Non-randomized Studies of Interventions, Version 5 July 2016. <http://www.riskofbias.info>. Accessed Sept 9th 2016.

32. Sterne JA, Hernan MA, Reeves BC, et al. ROBINS-I: a tool for assessing risk of bias in non-randomised studies of interventions. *BMJ* 2016; **355:** i4919.

33. Bayraktar S, Gonzalez-Angulo AM, Lei X, et al. Efficacy of neoadjuvant therapy with trastuzumab concurrent with anthracycline- and nonanthracycline-based regimens for HER2-positive breast cancer. *Cancer* 2012; **118:** 2385–93.

34. Gonzalez-Angulo AM, Parinyanitikul N, Lei X, et al. Effect of adjuvant trastuzumab among patients treated with anti-HER2-based neoadjuvant therapy. *British Journal of Cancer* 2015; **112:** 630–35.

35. Seferina SC, Lobbezoo DJ, de BM, et al. Real-life use and effectiveness of adjuvant trastuzumab in early breast cancer patients: a study of the Southeast Netherlands Breast Cancer Consortium. *Oncologist* 2015; **20:** 856–63.

36. Robidoux A, Tang G, Rastogi P, et al. Evaluation of lapatinib as a component of neoadjuvant therapy for HER2+ operable breast cancer: 5-year outcomes of NSABP protocol B-41. *J Clin Oncol* 2016; **34:** 501 (abstr).

37. Sparano JA, Wang M, Martino S, et al. Weekly paclitaxel in the adjuvant treatment of breast cancer. *N Engl J Med* 2008; **358:** 1663–71.

38. Slamon DJ, Eiermann W, Robert NJ, et al. Ten year follow-up of BCIRG-006 comparing doxorubicin plus cyclophosphamide followed by docetaxel (AC→T) with doxorubicin plus cyclophosphamide followed by docetaxel and trastuzumab (AC→TH) with docetaxel, carboplatin and trastuzumab (TCH) in HER2+ early breast cancer. *Cancer Res* 2016; **76:** S5–04 (abstr).

39. Piccart-Gebhart MJ, Procter M, Leyland-Jones B, et al. Trastuzumab after adjuvant chemotherapy in HER2-positive breast cancer. *N Engl J Med* 2005; **353:** 1659–72.

40. Perez EA, Romond EH, Suman VJ, et al. Four-year follow-up of trastuzumab plus adjuvant chemotherapy for operable human epidermal growth factor receptor 2-positive breast cancer: joint analysis of data from NCCTG N9831 and NSABP B-31. *J Clin Oncol* 2011; **29:** 3366–73.

41. Romond EH, Perez EA, Bryant J, et al. Trastuzumab plus adjuvant chemotherapy for operable HER2-positive breast cancer. *N Engl J Med* 2005; **353:** 1673–84.

42. Schmitz S, Adamsb R, Walsha C. Incorporating data from various trial designs into a mixed treatment comparison model. *Stat Med* 2013; **32:** 2935–49.

43. Jansen JP. Network meta-analysis of survival data with fractional polynomials. *BMC Med Res Methodol* 2011; **11:** 61.

44. Denduluri N, Somerfield MR, Eisen A, et al. Selection of optimal adjuvant chemotherapy regimens for human epidermal growth factor receptor 2 (HER2)-negative and adjuvant targeted therapy for HER2-positive breast cancers: an American Society of Clinical Oncology guideline adaptation of the Cancer Care Ontario clinical practice guideline. *J Clin Oncol* 2016; **34:** 2416–27.

45. Eisen A, Fletcher GG, Gandhi S, et al. Optimal systemic therapy for early breast cancer in women: a clinical practice guideline. *Curr Oncol* 2015; **22:** S67–81.
